# Distress, burden, and wellbeing in siblings of people with mental illness: a mixed studies systematic review and meta-analysis

**DOI:** 10.1017/S0033291723001733

**Published:** 2023-11

**Authors:** Anuradhi Jayasinghe, Anna Wrobel, Kate Filia, Linda K. Byrne, Glenn Melvin, Sean Murrihy, Carl Moller, Lesley Berk, Michael Berk, Sue Cotton

**Affiliations:** 1 School of Psychology, Deakin University, Geelong, Victoria, Australia; 2 Orygen, Parkville, Victoria, Australia; 3 Institute for Mental and Physical Health and Clinical Translation (IMPACT), School of Medicine, Deakin University, Geelong, Victoria, Australia; 4 Centre for Youth Mental Health, University of Melbourne, Parkville, Victoria, Australia; 5 Centre for Social and Early Emotional Development, Deakin University, Geelong, Victoria, Australia; 6 Centre for Educational Development Appraisal and Research, University of Warwick, Coventry, UK; 7 Melbourne School of Population and Global Health, University of Melbourne, Parkville, Victoria, Australia; 8 Florey Institute for Neuroscience and Mental Health, University of Melbourne, Melbourne, Victoria, Australia; 9 Department of Psychiatry, University of Melbourne, Royal Melbourne Hospital, Parkville, Victoria, Australia

**Keywords:** Burden, caregiver, distress, mental disorders, psychiatry, psychology, sibling, wellbeing

## Abstract

**Background:**

Family members of people with mental illness (MI) may experience a host of psychological adversities such as increased stress, burden, and reduced wellbeing. However, relatively little is known about siblings. This study aimed to characterise the experience of distress (viz. depressive and anxiety symptoms), burden, and wellbeing in siblings of people with MI.

**Methods:**

Studies reporting on quantitative measures of depression, anxiety, burden, or wellbeing in siblings; and/or qualitative findings on siblings’ experience were eligible. The literature search was conducted up until 20th October 2022.

**Results:**

Sixty-two studies comprising data from 3744 siblings were included. The pooled mean percentage of depressive symptoms fell in the mild range at 15.71 (*k* = 28, *N* = 2187, 95% CI 12.99–18.43) and anxiety symptoms fell in the minimal range at 22.45 (*k* = 16, *N* = 1122, 95% CI 17.09–27.80). Moderator analyses indicate that siblings of people with a schizophrenia spectrum disorder experience greater depressive symptoms than siblings of people with other types of MI (*β* = −16.38, *p* < 0.001). Qualitative findings suggest that individuals may be particularly vulnerable during their siblings’ illness onset and times of relapse. Limited communication, confusion about MI, and the need to compensate may contribute to siblings’ distress and/or burden. Siblings’ experience of wellbeing and caregiving were closely related.

**Conclusion:**

This review highlights the complex psychological experience of siblings and the need for greater research and clinical support for this important yet often overlooked cohort.

## Introduction

More than 12% of the global population live with a mental illness (MI; James et al., 2018; World Health Organisation, 2022, June 8). People with MI are more likely to experience physical illness, unemployment, homelessness, incarceration, and are at increased risk of suicide (World Health Organization, 2013). Deinstitutionalisation, (i.e. the replacement of long-stay psychiatric hospitals with smaller, community-based services), has occurred across most developed nations, resulting in many family members of people with MI shouldering a substantially greater burden of care (Bredewold, Hermus, & Trappenburg, 2020; Lin et al., 2018; Ohaeri, 2003). As a result, family members may experience a host of psychological adversities themselves such as stress, caregiver burden, helplessness, depression, and reduced wellbeing (Baronet, 1999; Fekadu, Mihiretu, Craig, & Fekadu, 2019; Maon, Horesh, & Gvion, 2020; Phillips, Durkin, Engward, Cable, & Iancu, 2022; Saunders, 2003). These psychological characteristics can be comprehensively conceptualised under three overarching domains: distress, burden, and wellbeing. Distress is the experience of negative psychological states characterised by symptoms of depression and anxiety (Drapeau, Marchand, & Beaulieu-Prévost, 2011; Ridner, 2004), while burden is the strain associated with providing care (Platt, 1985; Schene, 1990). Wellbeing refers to psychological health or ‘flourishing’ which comprises positive states, such as pleasure or joy, and an enduring sense of contentment with one's life (Marino, Haley, & Roth, 2017; Ryff & Keyes, 1995).

Aging parents often consider siblings to be the next-generation caregivers for their child(ren) with MI (Smith, Hatfield, & Miller, 2000). Siblings are individuals who share a parent or guardian via a biological or social relationship. Strong sibling relationships are associated with mental health benefits for both siblings (Buist, Deković, & Prinzie, 2013), and sibships are one of the most enduring types of relationship, often outlasting parental and marital bonds (Noller, 2005; Smith, Fadden, & O'Shea, 2009). As such, siblings can be an important and enduring protective factor in the lives of people with MI.

Despite their importance, relatively little is known about the psychological characteristics of siblings of people with MI. Previous narrative reviews of quantitative literature have produced mixed findings (Maon et al., 2020; Shivers & Textoris, 2021). For example, Shivers and Textoris (2021) found that some studies indicate siblings of people with MI experience significantly worse depressive symptoms and poorer quality sibships than samples of comparison individuals (Latzer, Katz, & Berger, 2015; Tschan, Ludtke, Schmid, & In-Albon, 2019), while others suggest siblings experience less emotional distress and sibling conflict (Jacoby & Heatherington, 2016; Zauszniewski & Bekhet, 2014); or no difference in internalisation, externalisation, expressed emotion, and emotionality (Hudson & Rapee, 2002; Kelvin, Goodyer, & Altham, 1996; Moulds et al., 2000). The synthesis of such broad-ranging constructs is likely to have contributed to the mixed findings in previous reviews and hinders our ability to elucidate the nature of specific psychological characteristics in siblings. A recent meta-analytic review conducted by our study team suggests that individuals with a sibling with MI experience significantly greater symptoms of depression and anxiety than those without (Jayasinghe et al., 2022). However, no review to date has synthesised quantitative findings on burden and wellbeing in siblings, and the extent of distress in siblings remains unclear. In a narrative review including qualitative literature on siblings of people with an eating disorder, Maon et al. (2020) found that reoccurring themes included worries about the future, guilt, sacrifice, and grief. Representing a clear gap, no review to date has examined qualitative findings on siblings of individuals with most other types of MI (e.g. schizophrenia spectrum, mood, personality, or trauma-related disorders).

Thus, the aim of this systematic review was to characterise distress, burden, and wellbeing in siblings of people with MI. To provide a comprehensive examination of the literature, a broad range of psychiatric illnesses were considered and both quantitative and qualitative data were synthesised using a novel sequential explanatory approach (Pluye & Hong, 2014). First, quantitative data were synthesised to estimate the extent of distress, burden, and wellbeing experienced by siblings. Subsequently, qualitative data were synthesised to contextualise the quantitative findings.

## Methods

In this study we address a subset of research questions stemming from a review of the psychological characteristics, viz., distress, burden, and wellbeing, of siblings of people with MI. The review was prospectively registered with the International Prospective Register of Systematic Reviews (CRD42020202757). The current study adheres to the Preferred Reporting Items for Systematic Reviews and Meta-Analyses (PRISMA 2020) guidelines (Page et al., 2021). PRISMA 2020 checklists can be found in Supplementary Material (online Supplementary Tables S1 and S2).

### Search strategy

The literature searches were conducted in MEDLINE Complete (EBSCOhost), PsycINFO (EBSCOhost), and Embase (EBSCOhost), with the final search occurring on 20th October 2022. The search terms included the concepts of siblings, mental illness, distress, burden, and wellbeing (online Supplementary Table S3). The search was limited to articles published in English with no restriction on publication date.

### Eligibility criteria

Studies were eligible for inclusion if they were full-text articles presenting original qualitative or quantitative findings. Studies were required to present either qualitative themes relating to the psychological experience of siblings or to report on the mean and standard deviation on a measure of distress (i.e. depressive or anxiety symptom severity – henceforth depressive or anxiety symptoms), burden, or wellbeing. Studies were also required to report on individuals aged 10 years or above who had at least one biological, step, adoptive, or foster sibling with MI. Mental illness was understood as any psychiatric disorder, including personality disorders and excluding neurodevelopmental and neurocognitive disorders. Studies were excluded if they reported on samples of fewer than 10 participants or if the available data were obtained via parent, guardian, or proband report. Online Supplementary Table S4 provides full details of the eligibility criteria.

### Study selection

AJ and AW independently screened titles and abstracts, followed by full-text articles, against the criteria specified for the broader review (online Supplementary Table S4). AJ and AW independently screened full-text articles against criteria for the current review (online Supplementary Table S4). Study authors were contacted if data on a quantitative outcome of interest were collected but not reported, and/or if further clarification was required (e.g. scoring of a measure). Ten authors (45.45% of requests) provided information. Where multiple publications reported on overlapping samples, the article reporting on the largest sample was reviewed unless the publications reported on unique outcomes of interest. Online Supplementary Table S5 provides a list of articles that fulfilled most but not all eligibility criteria.

### Data extraction

Data from each publication was extracted independently by at least two authors (AJ, AW, CM, or SM). Information on the characteristics of each study, sibling sample, and proband sample were extracted. For qualitative studies, authors extracted (sub)themes along with an illustrative quote, where possible. For studies where both themes and subthemes were provided, data was extracted only at the subtheme level to avoid the inclusion of overlapping qualitative data.

### Quality appraisal and certainty assessment

Authors (AJ, AW, SM and CM) independently used the Joanna Briggs Institute (JBI) Critical Appraisal Checklist for Analytical Cross Sectional Studies or the JBI Critical Appraisal Checklist for Qualitative Research to assess the methodological quality of included reports (Lockwood, Munn, & Porritt, 2015; Moola et al., 2017). An overall rating of ‘High’ or ‘Low’ was assigned to each publication. AJ utilised the Grading of Recommendation, Assessment, Development, and Evaluation (GRADE) framework to assess the certainty of evidence for each quantitative outcome (Guyatt et al., 2011). AW subsequently reviewed GRADE ratings.

Any disagreements during the screening, extraction, or quality appraisal phase were resolved via discussion between AJ, AW, SM, and CM, and/or with senior authors (KF, GM, LKB, and SC).

### Data analysis

**Figure 1.**
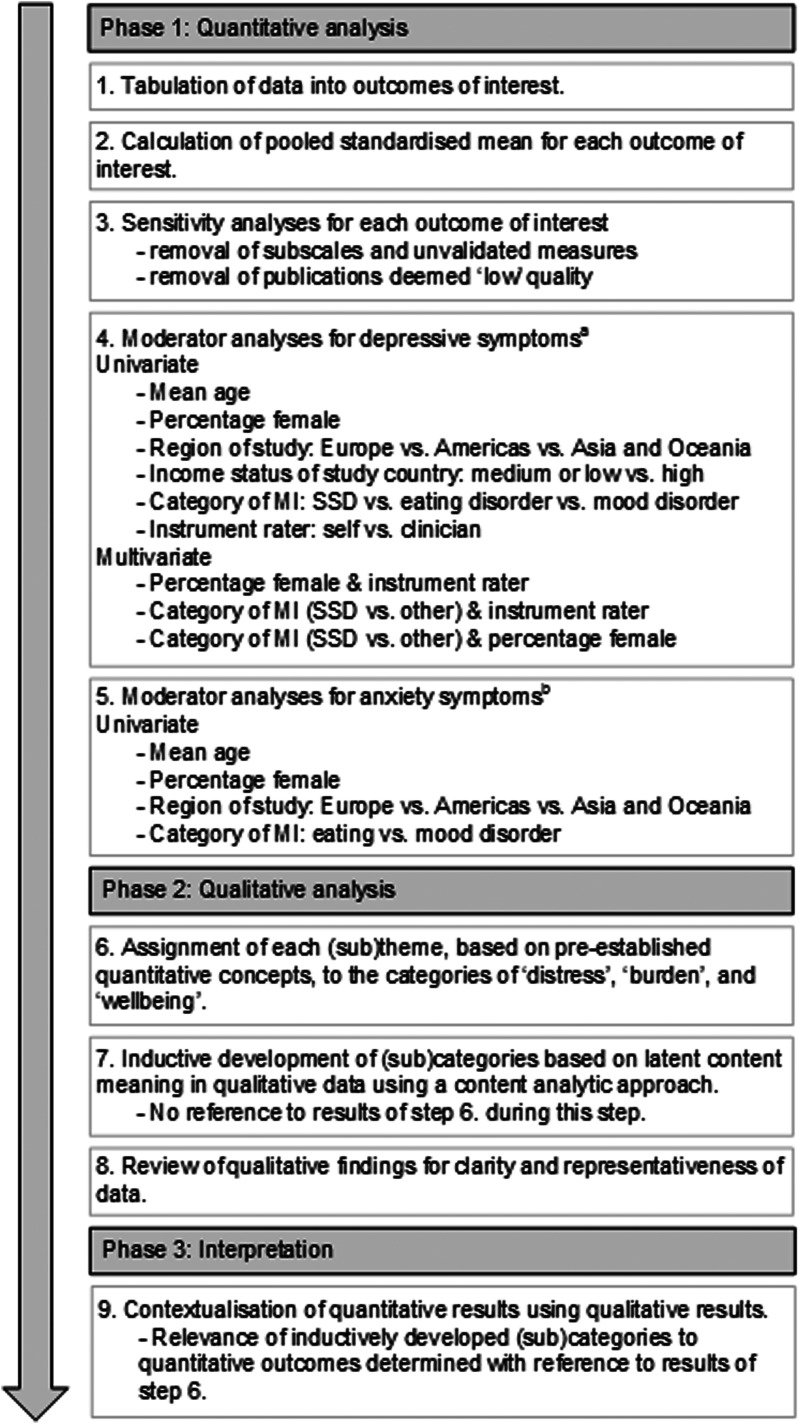
Sequential explanatory data analysis approach employed in the current review. *Note.* MI, mental illness; SSD, Schizophrenia spectrum disorder. ^a^Each analysis listed from step 2–4 was repeated for depressive symptoms with two outlying estimates of effect size removed to examine the impact of their inclusion. ^b^No further moderator analysis could be conducted for anxiety symptoms due to an insufficient number of included studies. No moderator analyses could be conducted for any burden or wellbeing outcomes due to an insufficient number of included studies.

As noted above, to characterise siblings’ experience of distress, burden, and wellbeing; we employed a sequential explanatory approach to data synthesis (Pluye & Hong, 2014). Quantitative findings were synthesised first and used to guide our analysis of qualitative findings. Results of the qualitative analysis were then used to contextualise quantitative findings. Although the contextualisation of quantitative findings using qualitative findings could be considered results of this review (Pluye, Bengoechea, Granikov, Kaur, & Tang, 2018; Pluye & Hong, 2014), this content has been presented as part of the discussion section due to the dearth of other relevant literature in this field. Figure 1 illustrates the data analysis process and online Supplementary Table S6 provides further details.

#### Quantitative analysis

Quantitative data were tabulated according to the following a priori groupings: depressive symptoms, anxiety symptoms, burden (overall), burden (positive aspects), burden (negative aspects), burden (objective), burden (subjective), wellbeing (negative affect), wellbeing (positive affect), and wellbeing (eudemonic). Burden was initially conceptualised as comprising two major components: objective and subjective burden (Hoenig & Hamilton, 1966). Objective burden refers to observable difficulties such as loss of employment while subjective burden refers to an individual's evaluation of the strain associated with caregiving (Platt, 1985). More recently, burden has been considered within a stress-appraisal-coping framework which posits that the impact of caregiving is dependent on an individual's appraisal of their experience (Lazarus & Folkman, 1984). With this shift, new measures of burden were developed to capture the negative and positive aspects of caregiving (Szmukler et al., 1996). The above listed groupings of burden were utilised given that available measures – while highly correlated – capture distinct facets (Schulze & Rössler, 2005). Similarly, two types of wellbeing are commonly recognised: hedonic and eudemonic (Marino et al., 2017; Ryff, 1989). Hedonic wellbeing refers to fleeting states that are often captured in three dimensions: the presence of positive affective states, the absence of negative affective states, and feelings of uplift typically referred to as ‘satisfaction with life’ (Marino et al., 2017). Eudemonic wellbeing, in contrast, pertains to a lasting sense of contentment (Ryff & Keyes, 1995). In line with prior reviews (e.g. van Agteren et al., 2021), the outlined groupings were selected to capture these aspects of wellbeing.

Statistical analyses were conducted using Comprehensive Meta-Analysis Software Version 3 (Borenstein, Hedges, Higgins, & Rothstein, 2013). To calculate the pooled mean, we used a random-effects model for each outcome of interest as per the above listed groupings. To allow for comparability across measures, raw scores were standardised prior to meta-analysis. Scores were standardised by calculating a fraction using the mean or standard deviation as the numerator and the possible range of scores on the relevant measure as the denominator (Bath, Deeg, & Poppelaars, 2010). The fraction was then multiplied by 100. Based on cohesiveness with the measures used in this body of literature, the Hamilton Rating Scale for Depression (HAM-D) and Anxiety (HAM-A) were selected to facilitate the interpretation of depressive and anxiety symptom data. To do so, cut-off scores on these measures were standardised as outlined above. This resulted in the following ranges: HAM-D 0–13 minimal; 14–30 mild; 31–44 moderate; 45+ severe; and HAM-A 0–29 minimal; 30–43 mild; 44+ moderate to severe (Thompson, 2015; Zimmerman, Martinez, Young, Chelminski, & Dalrymple, 2013). Where the analysis included >10 publications, we assessed small study bias visually via inspection of funnel plot asymmetry, and statistically using Egger's regression test (Higgins et al., 2022, August 4). We used Cochran's Q to assess the significance of heterogeneity across studies and Higgins *I*^2^ to examine the extent of heterogeneity with the following interpretations: *I*^2^<40% = low; 40–75% = moderate; and >75% = high variance in effect size (Higgins et al., 2022, August 4).

Several sensitivity and moderator analyses were conducted to provide a nuanced evaluation of the quantitative data (Fig. 1). A detailed description of these analyses can be found in online Supplementary Table S6.

#### Qualitative analysis

Each extracted (sub)theme was assigned a credibility rating of ‘unequivocal’, ‘credible’, or ‘not supported’ based on the (sub)theme's name, description, and illustrative quote as presented in the original publication (online Supplementary Table S7; Lockwood et al., 2020). A rating of ‘unequivocal’ was assigned where there was clear cohesion between (sub)theme name, description, and illustrative quote. ‘Credible’ was assigned where there were some doubts about the association between information captured in a (sub)theme name, description, and illustrative quote. ‘Not supported’ was assigned where there was little or no association between captured information. (Sub)themes with a rating of ‘not supported’ were excluded from the subsequent analysis. Included (sub)themes were assigned to a category based on the established quantitative concepts of distress, burden, and wellbeing.

AJ and AW synthesised (sub)themes using an inductive content meta-analysis approach (Cho & Lee, 2014; Mayring, 2000). In this approach, new categories and subcategories were inductively developed based on the latent content meaning of extracted primary data. This process involved ongoing revision of the emerging (sub)categories with the progressive incorporation of primary data. SM reviewed the preliminary results of the synthesis to assess for clarity and representativeness of primary data. Results were then tabulated and narratively reviewed. With reference to the initial classifications based on the concepts of distress, burden, and wellbeing (see online Supplementary Table S7); results of the content meta-analysis were used to contextualise quantitative meta-analytic findings (e.g. where a subcategory was primarily comprised of extracted data classified as ‘burden’, this subcategory was used to contextualise quantitative findings on burden).

To enrich our interpretation of qualitative data, we utilised repeated readings during all stages of extraction and synthesis. Any disagreements were resolved via discussions (between AJ, AW, SM, CM, KF, LKB, GM, and/or SC) that aimed to incorporate multiple interpretations of the data.

## Results

### Literature search

**Figure 2.**
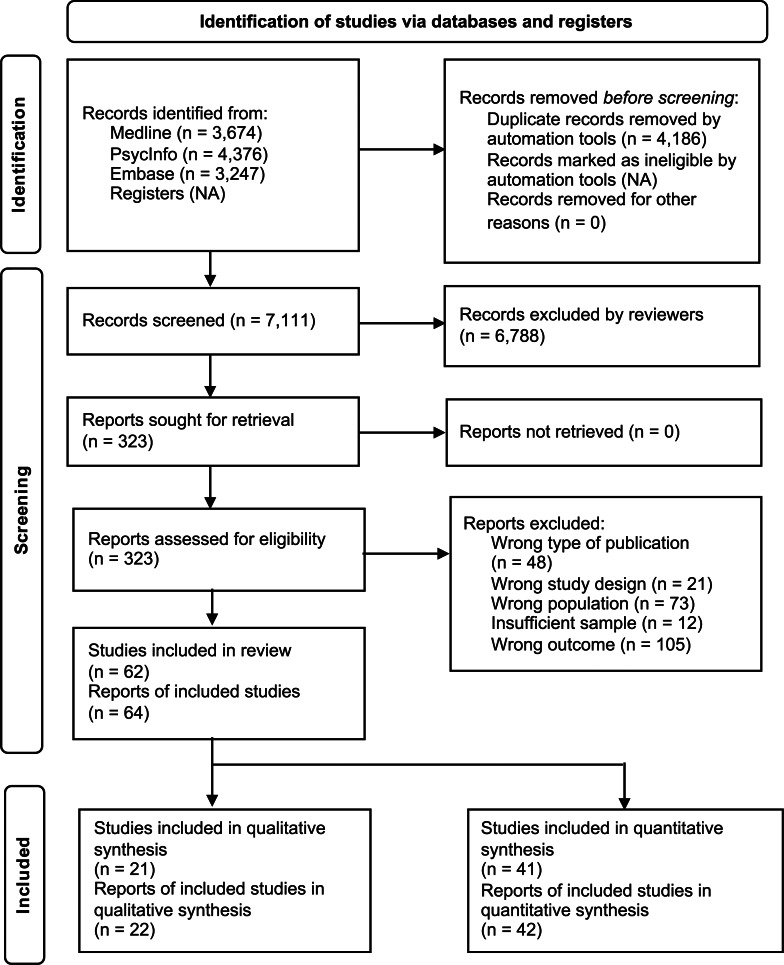
Preferred reporting items for systematic reviews and Meta-Analyses (PRISMA) flow diagram.

The study selection process resulted in the inclusion of 64 publications from 62 studies (Fig. 2). Forty-one studies provided quantitative data and 21 studies provided qualitative data. Three publications used mixed methods; however, one reported only quantitative data and two reported only qualitative data that were eligible for inclusion.

### Study characteristics

Included studies comprised data from 3744 siblings. Based on available data, siblings were on average 32.76 years old (s.d. = 16.32; *n* = 2784) and 59.70% were female (*n* = 2199). Studies predominantly considered siblings of people with a schizophrenia spectrum disorder (*k* = 22) and most were conducted in Europe (*k* = 26). Tables 1 and 2 present key characteristics for included publications and online Supplementary Tables S8–S11 detail additional information on siblings, probands, and sibships for each report.

**Table 1.** Study characteristics and outcomes for included quantitative publications

Study	Country	Sample *N*	Age *M* (s.d.)	% female	MI^a^	Diagnostic method	Outcome	Measure	Raw score *M* (s.d.)
Alzahrani et al. (2017)	Saudi Arabia	150	NR	NR	MI	Clinician appraisal	Burden (overall)	IEQ	37.10 (18.60)
Amaresha et al. (2018)	India	80	30.03 (6.00)	20.00	Schizophrenia	Structured interview	Depressive symptoms	BDI	2.51 (2.77)
							Burden (overall)	BAS	61.56 (10.21)
Amianto et al. (2011)	Italy	31	26.58 (8.30)	70.97	Anorexia Nervosa	Structured interview	Anxiety symptoms	SCL-90-R	6.88 (6.11)
								BDI	2.51 (2.77)
Avcıoğlu et al. (2019)	Turkey	103	37.14 (11.16)	42.72	Schizophrenia	NR	Burden (overall)	ZCBS	3.45 (0.88)
							Wellbeing (eudemonic)	SWS	3.81 (0.83)
Barak and Solomon (2005)	Israel	52	NR	68.00	Schizophrenia	NR	Burden (objective)	BAS	2.35 (0.63)
							Burden (subjective)	BAS	2.53 (0.51)
Barrett et al. (2004)	Australia	32	11.49 (2.96)	62.50	OCD	NR	Depressive symptoms	CDI	6.55 (5.14)
							Anxiety symptoms	MASC	43.69 (13.75)
Bowman et al. (2017)	Australia	157	21.76 (4.38)	48.40	MI: 84.60% SSD	Clinician appraisal	Burden (negative aspects)	ECI	97.66 (5.30)
							Burden (positive aspects)	ECI	30.58 (1.52)
Boyette et al. (2013)	Netherlands	281	NR	58.30	SSD	NR	Depressive symptoms	CAPE	4.24 (3.19)
Casper (1990)	USA	15	26.20 (5.20)	100	Anorexia Nervosa	Clinician appraisal	Depressive symptoms	BDI	5.00 (5.10)
							Anxiety symptoms	SCL-90-R	0.60 (0.70)
Chen and Lukens (2011)	USA	32	42.38 (10.38)	75.00	MI: 81.80% SSD	NR	Depressive symptoms	CES-D	16.84 (5.05)
Christensen et al. (2007)	Denmark	128	45.68 (13.22)	64.84	Affective Disorders	Clinician appraisal	Depressive symptoms	HAM-D^b^	3.28 (2.01)
							Anxiety symptoms	BAI-14	1.58 (2.30)
Deal and MacLean (1995)	USA	15	11.40 (2.60)	46.67	MI: 33.34% Depressive Disorder 26.66% Disruptive Disorders	NR	Depressive symptoms	CDI	6.60 (4.70)
Di Sarno et al. (2021)	Brazil	16	47.25 (8.59)	NR	Schizophrenia	Clinician appraisal	Burden (objective)	FBIS	2.53 (0.61)
							Burden (subjective)	FBIS	2.19 (0.45)
Diaz et al. (2021)	USA	24	36.08 (11.68)	70.80	Bipolar Disorder	Structured interview	Depressive symptoms	MADRS	2.33 (5.04)
Fekih-Romdhane et al. (2020)	Tunisia	60	23.00 (2.70)	37.00	Schizophrenia	NR	Depressive symptoms	DASS-21	12.70 (10.80)
							Anxiety symptoms	DASS-21	9.30 (8.90)
Fox et al. (2022)	Australia	36	10.40 (2.75)	NR	Anxiety Disorder	Structured interview	Anxiety symptoms	RCMAS	7.58 (6.30)
Geller et al. (2017)	Canada	19	22.94 (7.73)	NR	Eating Disorder	NR	Depressive symptoms	BSI	0.89 (0.61)
							Anxiety symptoms	BSI	0.84 (0.52)
							Burden (positive aspects)	ECI	6.73 (1.45)
							Burden (negative aspects)	ECI	32.92 (7.70)
Kovacs et al. (2016)	Hungary	207	15.94 (2.12)	55.10	Depressive Disorder	Structured interview	Depressive symptoms	CDI-2	8.67 (5.96)
							Wellbeing (positive affect)	PANAS^c^	29.11 (6.22)
							Wellbeing (negative affect)	PANAS^c^	17.95 (6.20)
Laporte et al. (2011)	Canada	56	30.20 (NR)	100	BPD	Structured interview	Depressive symptoms	HAM-D	1.69 (2.70)
							Anxiety symptoms	HAM-A	1.59 (2.40)
Lataster et al. (2010)	Netherlands & Belgium	47	31.10 (9.40)	60.00	SSD	Structured interview	Wellbeing (negative affect)	ESM^c^	0.99 (0.50)
Latzer et al. (2015)	Israel	30	21.67 (4.48)	100	Eating Disorder	Clinician appraisal	Depressive symptoms	BDI	8.17 (5.57)
Mahon et al. (2013)	USA	53	40.70 (11.70)	64.20	Bipolar Disorder	Structured interview	Depressive symptoms	HAM-D	3.10 (2.90)
Matthews et al. (2021)	USA	34	15.11 (2.18)	70.60	Anorexia Nervosa	Clinician appraisal	Depressive symptoms	CDI-2S	4.32 (3.53)
							Anxiety symptoms	MASC-2	67.39 (23.19)
Meneguzzo et al. (2022)	Italy	57	21.52 (6.91)	100	Eating Disorder	Clinician appraisal	Depressive symptoms	SCL-58-R	1.07 (0.81)
							Anxiety symptoms	SCL-58-R	1.03 (0.77)
Modestin et al. (2008)	Switzerland	27	38.00 (13.00)	62.96	MI: 37.00% SSD	Clinician appraisal	Depressive symptoms	SCL-90-R	0.71 (0.75)
							Anxiety symptoms	SCL-90-R	0.53 (0.77)
Panaite et al. (2019)	Hungary	198	15.92 (2.15)	53.50	Depressive Disorder	NR	Anxiety symptoms	MASC	34.07 (14.16)
Phillipou et al. (2022)	Australia	20	22.81 (2.90)	100	Anorexia Nervosa	Structured interview	Depressive symptoms	DASS-42	6.74 (8.16)
							Anxiety symptoms	DASS-42	7.45 (7.78)
Phoeun et al. (2022)	Cambodia	19	NR	NR	MI	NR	Depressive symptoms	PHQ-9	6.84 (5.98)
							Anxiety symptoms	GAD-7	6.58 (5.64)
Pignon et al. (2021)	Brazil, France, Italy, Netherland, Spain, UK	265	31.30 (9.40)	31.70	FEP	Clinician appraisal	Depressive symptoms	CAPE	4.20 (2.10)
Ragazzi et al. (2020)	Brazil	104	30.90 (11.10)	72.10	SSD	Structured interview	Depressive symptoms	CAPE	13.70 (4.80)
Reinhard and Horwitz (1995)	USA	77	36.30 (NR)	64.00	MI: 51.00% Schizophrenia	NR	Burden (overall)	BAS	16.90 (9.90)
Schick et al. (2022)	Netherlands & Belgium	79	53.26 (8.68)	63.29	SSD	NR	Wellbeing (positive affect)	ESM^c^	4.82 (1.07)
							Wellbeing (negative affect)	ESM^c^	1.44 (0.75)
Shivers et al. (2022)	USA	64	NR	NR	MI	NR	Burden	BSFC-S	18.75 (7.19)
Sin et al. (2016)	UK	90	27.52 (8.41)	85.10	SSD	NR	Burden (positive aspects)	ECI	31.97 (8.23)
							Burden (negative aspects)	ECI	101.38 (30.49)
							Wellbeing (eudemonic)	WEMWBS	46.81 (9.79)
Sletved et al. (2022)	Denmark	129	29.20 (NR)	65.50	Bipolar Disorder	Structured interview	Depressive symptoms	HAM-D	2.70 (3.42)
Smith et al. (2016)	USA	41	23.50 (4.60)	53.70	Schizophrenia	Structured interview	Depressive symptoms	CES-D	12.20 (9.00)
							Burden (objective)^d^		8.80 (4.80)
							Burden (subjective)^d^		7.30 (3.90)
Tanaka (2008)	USA & Japan	130	43.34 (13.41)	76.15	MI: 94.62% SSD	Sibling report	Burden (positive aspects)	ECI (adapted)	3.04 (0.69)
							Burden (negative aspects)	BAS (adapted)	2.13 (0.57)
Tatay-Manteiga et al. (2019)	Spain	23	41.50 (11.80)	69.60	Bipolar Disorder	Structured interview	Depressive symptoms	HAM-D	0.90 (1.30)
Taylor et al. (2008)	USA	83	63.87 (4.42)	55.40	MI: 93.40% Affective Disorders	Proband report	Depressive symptoms	CES-D	7.62 (1.13)
							Wellbeing (eudemonic)	RPB	4.96 (0.63)
van Sprang et al. (2021)	Netherlands	380	50.46 (13.25)	55.00	Depressive & anxiety disorders	Structured interview	Depressive symptoms	IDS-SR	13.23 (10.00)
							Anxiety symptoms	BAI^e^	1.97 (2.97)
Zauszniewski and Bekhet (2014)	USA	14	NR	100	MI	Sibling report	Depressive symptoms	ESC	0.57 (NR)
							Anxiety symptoms	ESC	1.21 (NR)
Zhang et al. (2022)	China	27	37.78 (11.74)	59.26	Depressive Disorder	Structured interview	Depressive symptoms	HAM-D	1.41 (1.71)

*N*, sample size; *M*, mean; s.d., standard deviation; MI, mental illness; NR, not reported; IEQ, Involvement Evaluation Questionnaire; BDI, Beck Depression Inventory; BAS, Burden Assessment Schedule; SCL-90-R, The Symptom Checklist-90 Item-Revised; ZCBS, Zarit Caregiver Burden Scale; SWS, Subjective Wellbeing Scale; OCD, Obsessive-compulsive disorder; CDI, Children's Depression Inventory; MASC, Multidimensional Anxiety Scale for Children; ECI, The Experience of Caregiving Inventory; SSD, Schizophrenia Spectrum Disorder; CAPE, Community Assessment of Psychic Experience; USA, United States of America; CES-D, Center for Epidemiological Studies Depression Scale; HAM-D, Hamilton Depression Rating Scale; BAI-14, Beck 14-item Anxiety Inventory; FBIS, Family Burden Interview Schedule; MADRS, Montgomery-Asberg Depression Rating Scale; DASS-21, 21-item Depression Anxiety Stress Scale; RCMAS, Revised Children's Manifest Anxiety Scale; BSI, Brief Symptom Inventory; CDI-2, Children's Depression Inventory Second Edition; PANAS, Positive and Negative Affect Schedule; BPD, Borderline Personality Disorder; HAM-A, Hamilton Anxiety Rating Scale; ESM, Experience Sampling Method; CDI-2S, Children's Depression Inventory Second Edition Short Form; SCL-58-R, The Symptom Checklist-58 Item-Revised; MASC-2, Multidimensional Anxiety Scale for Children Second Edition; DASS-42, 42-item Depression Anxiety Stress Scale; PHQ-9, Patient Health Questionnaire; GAD-7, Generalised Anxiety Disorder Assessment; UK, United Kingdom; FEP, first episode psychosis; BSFC-S, Burden Scale for Family Caregivers; WEMWBS, The Warwick-Edinburgh Mental Wellbeing Scale; RPB, The Ryff Scales of Psychological Wellbeing; IDS-SR, The Inventory of Depressive Symptomatology-Self-Report; ESC, Emotional Symptom Checklist.

a Studies described as ‘MI’ only provided no additional information about the composition of their sample. Full details for all studies are provided in online Supplementary Table S10.

b Authors of the reviewed study utilised multiple measures of depressive symptoms. The Hamilton Depression Rating Scale was deemed most cohesive with study aims and other eligible publication; and was therefore retained.

c Although the Positive and Negative Affect Schedule and the Experience Sampling Method are notably distinct measures of affect, results were pooled in this review due to the small number of studies measuring affect in siblings of people with MI.

d Author of the reviewed study utilised an untitled, purpose-built measure.

e Study authors utilised multiple measures of anxiety. The Beck Anxiety Inventory was deemed most cohesive with study aims and other eligible publication; and was therefore retained.

**Table 2.** Study characteristics for included qualitative studies

Study	Country	Sample *N*	Age *M* (s.d.)	% female	Mental illness^a^	Diagnostic method	Method
Amaresha et al. (2019)	India	15	33.00 (6.72)	26.67	Schizophrenia	NR	Not reported
Areemit et al. (2010)	Canada	10	13.50 (2.22)	70.00	Eating Disorder	NR	Grounded Theory
Dimitropoulos et al. (2009)	Canada	12	25.60 (7.85)	100	Anorexia Nervosa	Clinician appraisal	Ground Theory
Ewertzon et al. (2012)	Sweden	13	45.00 (NR)	84.62	SSD	NR	Content analysis
Fjermestad et al. (2020)	Norway	13	15.50 (3.00)	76.92	Anorexia Nervosa	NR	Phenomenological
Friedrich et al. (1999)	USA	30	37.00 (NR)	50.00	Schizophrenia	NR	Not reported
Gerace et al. (1993)	NR	14	35.00 (NR)	78.57	Schizophrenia	NR	Content and thematic analysis
Hutchison et al. (2022)	UK	14	14.90 (NR)	51.14	Eating Disorder	NR	Thematic analysis
Jungbauer et al. (2016)	Germany	16	24.30 (7.60)	75.00	Anorexia Nervosa	NR	Content analysis
Karlstad et al. (2021)	Norway	10	25.10 (3.90)	70.00	Eating Disorder	NR	Grounded Theory
Kovacs et al. (2019)	Israel	14	NR	50.00	Mental illness	Sibling report	Phenomenological
Kristoffersen and Mustard (2000)	Norway	16	34.00 (NR)	37.50	Schizophrenia	Clinician appraisal	Hermeneutical
Lukens et al. (2002)	USA	19	43.00 (NR)	84.21	Mental illness: 68.42% SSD	NR	Grounded Theory
Lukens et al. (2004)	USA	19	43.00 (NR)	84.21	Mental Illness: 68.42% SSD	Sibling report	Grounded Theory
Persico et al. (2021)	France	7	12.71 (3.68)	57.14	Anorexia Nervosa	NR	Phenomenological
Riebschleger (1991)	USA	20	35.00 (NR)	55.00	Mental illness: 71.43% Schizophrenia	NR	Not reported
Samuels and Chase (1979)	NR	11	33.50 (NR)	72.73	Schizophrenia	Sibling report	Not reported
Schmid et al. (2009)	Germany	37	41.60 (NR)	64.90	Schizophrenia	Clinician appraisal	Content analysis
Scutt et al. (2022)	UK	10	26.70 (NR)	NR	Anorexia Nervosa	Sibling report	Thematic analysis
Sin et al. (2008)	UK	10	22.80 (NR)	80.00	FEP	NR	Phenomenological
Sin et al. (2012)	UK	31	22.70 (6.56)	70.97	FEP	NR	Phenomenological
Stålberg et al. (2004)	Sweden	16	31.00 (NR)	50.00	SSD	NR	Grounded Theory

*N*, sample size; *M*, mean; s.d., standard deviation; NR, not reported; SSD, Schizophrenia spectrum disorder; USA, United States of America; UK, United Kingdom; FEP, First episode psychosis.

a Studies described as ‘MI’ only provided no additional information about the composition of their sample. Full details for all studies are provided in online Supplementary Table S10.

### Quality appraisal

Of the 42 publications contributing to the quantitative synthesis, 28 were deemed ‘High’ quality and 14 ‘Low’ (see online Supplementary Table S12 for full details). Most studies (*k* = 39) employed a validated measure of depressive symptoms, anxiety symptoms, burden, and/or wellbeing. In contrast, studies varied in their description of eligibility criteria, as well as their characterisation of siblings and probands. Only 15 studies (35.71%) employed a structured clinical interview to determine the presence of a MI in probands.

Of the 22 reports included in the qualitative synthesis, seven were considered High quality while the remainder were evaluated Low (online Supplementary Table S11). Most publications (*k* = 15) represented participants’ accounts well by providing illustrative quotes to support the findings of the research. However, studies varied in reporting research methodology. As a result, the congruity between research methodology and philosophical perspective, research question(s), data collection methods, and approach to data analysis in many (*k* = 9) publications was unclear. Finally, representing a major area of concern, only one report discussed the influence of the authors on the research and no study provided a statement locating the authors culturally and/or theoretically.

### Quantitative results

#### Distress

**Figure 3.**
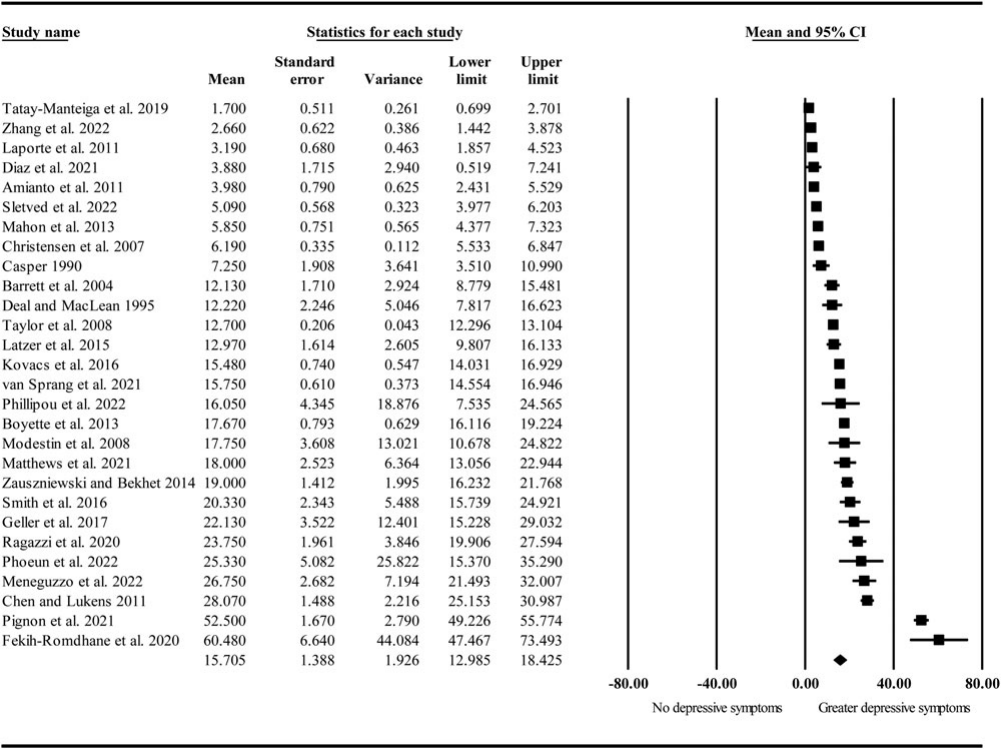
Forrest plot of standardised mean depressive symptoms in siblings of people with mental illness.

**Depressive symptoms.** After standardisation, the pooled mean for depressive symptoms was 15.71 (Table 3 and Fig. 3). In univariate meta-regression analyses (Table 4), the mean age, region of study, and income status of study country were not significant moderators of effect sizes (*p* = 0.413, *p* = 0.903, and *p* = 0.096 respectively). Estimates of effect size were significantly moderated by the proportion of female participants, category of mental illness (schizophrenia spectrum v. mood *v.* eating disorders), and instrument rater (clinician *v.* self-rated). Mean depressive symptoms were significantly higher in samples with greater proportions of male participants (i.e. male siblings of people with MI, *β* = −0.25, *p* = 0.041, *R*^2^ = 0.13). Category of mental illness accounted for 52% of variance in effect sizes, with mean depressive symptoms being significantly higher in studies considering siblings of people with a schizophrenia spectrum disorder compared to an eating disorder (*β* = 17.16, *p* = 0.0007) or a mood disorder (*β* = 22.26, *p* < 0.001). Finally, mean depressive symptoms were significantly greater in studies using a self-report measure compared to those using a clinician-rated instrument (*β* = 16.29, *p* = 0.0002, *R*^2^ = 0.36).

**Table 3.** Pooled effect sizes, and measures of heterogeneity and small study bias for quantitative outcomes of interest

Outcome	*k*	*N*	Effect size		Heterogeneity				Small study bias	
			Pooled *M* [95% CI]	*p*-value	*Q*	*df*	*p*-value	*I*^2^	Egger's t	*p*-value
Depressive symptoms	28	2187	15.71 [12.99–18.43]	<0.001	2262.31	27	<0.001	98.81	1.37	0.091
Anxiety symptoms	16	1122	22.45 [17.09–27.80]	<0.001	1647.75	15	<0.001	99.09	3.57	0.002
Burden (overall)	5	474	36.27 [25.14–47.39]	<0.001	186.46	4	<0.001	97.89		
Burden (negative aspects)	4	396	37.29 [19.78–54.80]	<0.001	1296.78	3	<0.001	99.77		
Burden (positive aspects)	4	396	49.91 [23.10–76.72]	<0.001	4855.15	3	<0.001	99.94		
Burden (objective)	3	109	40.19 [34.82–45.56]	<0.001	4.24	2	0.120	52.82		
Burden (subjective)	3	107	41.97 [31.36–52.58]	<0.001	19.78	2	<0.001	89.89		
Wellbeing (negative affect)	3	366	14.64 [8.22–21.05]	<0.001	51.18	2	<0.001	96.09		
Wellbeing (positive affect)	2	286	55.64 [40.06–71.21]	<0.001	48.63	1	<0.001	97.94		
Wellbeing (eudemonic)	3	276	69.39 [56.90–81.87]	<0.001	80.46	2	<0.001	97.51		

*k*, number of independent samples; *N*, number of participants; *M*, mean; 95% CI, 95% confidence interval; *Q*, statistic for within-studies heterogeneity; *df*, degrees of freedom; *I*^2^, statistic for between-studies heterogeneity.

**Table 4.** Moderator analyses for depressive and anxiety symptoms

Covariate	*k*	*N*	*β* [95% CI]	*p-*value	*QM*	*df*_QM_	*P_QM_-value*	*QE*	*df*_QE_	*P*_QE_-value	*I*^2^	*T*^2^	*R*^2^
Depressive symptoms (univariate analyses)													
Age	28	2187	−0.16 [−0.55 to 0.23]		0.67	1	0.413	2262.31	27	<0.001	98.81	154.91	0.02
Percentage female	28	2187	−0.25 [−0.48 to −0.01]		4.17	1	0.041	1894.13	26	<0.001	98.61	135.43	0.13
Region of study	26	2097			0.20	2	0.903	2201.77	25	<0.001	98.86	116.26	0.01
Europe			16.14 [9.41–22.86]	<0.001									
Europe vs. Americas			−1.53 [−10.65 to 7.59]	0.742									
Europe vs. Asia and Oceania			−2.71 [−15.62 to 10.19]	0.68									
Income status of study country	28	2187			2.77	1	0.096	2262.31	27	<0.001	98.81	154.91	0.07
Medium or low-income status			26.10 [13.68–38.53]	<0.001									
Medium or low vs. high-income status			−11.33 [−24.68 to 2.01]	0.096									
Category of MI	26	2112			24.96	2	<0.001	2144.47	25	<0.001	98.83	157.58	0.52
SSD			32.19 [24.87–39.52]	<0.001									
SSD vs. eating disorder			−17.16 [−27.12 to −7.20]	0.001									
SSD vs. mood disorder			−22.26 [−31.02 to −13.50]	<0.001									
Instrument rater	28	2187			13.82	1	<0.001	2262.31	27	<0.001	98.81	154.91	0.36
Self-report			20.37 [15.98–24.75]	<0.001									
Self-report vs. clinician-rated			−16.28 [−24.87 to −7.70]	<0.001									
Depressive symptoms (multivariate analyses)													
Percentage female and instrument rater	28	2187			21.96	2	<0.001	2262.31	27	<0.001	98.81	154.91	0.47
Intercept			35.98 [22.42–49.54]	<0.001									
Percentage female			−0.23 [−0.41 to −0.04]	0.018									
Instrument rater: clinician			−15.96 [−23.82 to −8.10]	<0.001									
Category of MI and instrument rater	28	2187			41.03	2	<0.001	2262.31	27	<0.001	98.81	154.91	0.63
Intercept			31.85 [25.46–38.25]	<0.001									
Category of MI: non-SSD			−16.38 [−23.92 to −8.83]	0.001									
Instrument rater: clinician			−11.40 [−18.28 to −4.51]	<0.001									
Category of MI and percentage female	28	2187			22.96	2	<0.001	2262.31	27	<0.001	98.81	154.91	0.48
Intercept			36.26 [22.77–49.75]	<0.001									
Category of MI: non-SSD			−19.23 [−28.43 to −10.02]	<0.001									
Percentage female			−0.07 [−0.27 to 0.13]	0.484									
Anxiety symptoms (univariate analyses)													
Age	16	1122	−0.80 [−1.19 to −0.41]		16.21	1	<0.001	1647.75	15	<0.001	99.09	162.74	0.54
Percentage female	16	1122	−0.17 [−0.50 to 0.16]		1.00	1	0.317	1647.75	15	<0.001	99.09	162.74	0.05
Region of study	15	1062			9.36	2	0.009	1597.81	14	<0.001	99.12	146.67	0.40
Americas			10.21 [0.66–19.76]	0.036									
Americas vs. Asia and Oceania			21.43 [7.65–35.21]	0.002									
Americas vs. Europe			11.66 [−0.33 to 23.65]	0.057									
Category of MI	13	987			0.33	1	0.567	1554.64	12	<0.001	99.23	134.69	0.03
Mood disorder			20.40 [11.76–29.06]	<0.001									
Eating disorder			3.76 [−9.11 to 16.64]	0.567									

*k*, number of independent samples; *N*, number of participants; *β*, coefficient; 95% CI, 95% confidence interval; *QM*, test of statistical significance of the moderators; *df*, degrees of freedom; *QE*, test of total between-studies variance (intercept only); *I*^2^, statistic for between-studies heterogeneity; *T*^2^, Tau squared; *R*^2^, proportion of total between-studies variance explained by the model.

In a subsequent multivariate meta-regression analysis of category of mental illness (schizophrenia spectrum disorder *v.* other) and proportion of female participants, only category of mental illness remained a significant covariate (*β* = −19.23, *p* < 0.001, *R*^2^ = 0.48). That is, depressive symptoms were significantly higher in siblings of people with a schizophrenia spectrum disorder compared to other MIs irrespective of the proportion of female participants. In an analysis of proportion of female participants and instrument rater, both covariates remained significant (*β* = −0.23, *p* = 0.018 and *β* = −15.96, *p* < 0.001 respectively; *R*^2^ = 0.47). Similarly, in an analysis of category of mental illness and instrument rater, both covariates were significant (*β* = −16.38, *p* < 0.001 and *β* = −11.40, *p* = 0.001 respectively; *R*^2^ = 0.63). Importantly, when two outlying effects (Fekih-Romdhane, Nsibi, Sassi, & Cheour, 2020; Pignon et al., 2021) were removed from the analysis, only category of mental illness and instrument rater remained significant covariates. Outlying estimates were identified using Tukey's method in SPSS (IBM Corp, 2020; Tukey, 1977). Full details of all sensitivity analyses are provided in online Supplementary material (Sections 2–5).

**Figure 4.**
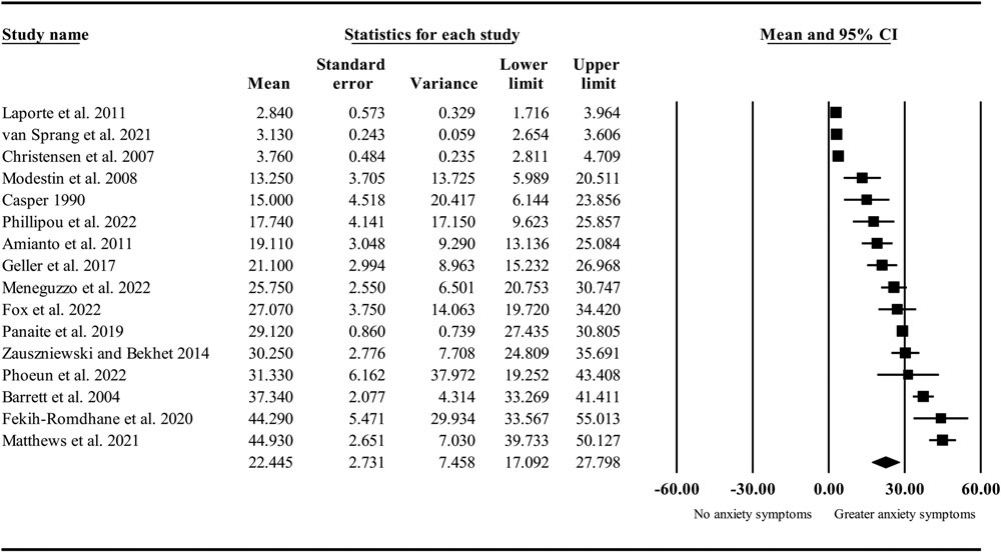
Forrest plot of standardised mean anxiety symptoms in siblings of people with mental illness.

**Anxiety symptoms.** The pooled standardised mean for anxiety symptoms was 22.45 (Table 3 and Fig. 4). A univariate meta-regression analysis indicated that sibling age accounted for 54% of variance in effect sizes, with mean anxiety symptoms significantly decreasing with mean age of siblings (*β* = −0.80, *p* < 0.001; Table 4). A further univariate analysis indicated that region of study accounted for 40% of variance in effect sizes (*p* = 0.009, Table 4). Effect sizes were significantly higher in studies conducted in Asia and Oceania compared to studies conducted in the Americas (*β* = 21.43, *p* = 0.002). No significant difference was found in studies conducted in the Americas compared to Europe (*p* = 0.057). Proportion of female participants and category of mental illness (mood *v.* eating disorders) were not significant moderators (*p* = 0.317 and *p* = 0.567).

No further meta-regression analyses were conducted across all outcomes of interest due to an insufficient number of included studies.

#### Burden and wellbeing

The pooled standardised means for burden outcomes were: 36.27 (overall burden); 37.29 (negative aspects); 49.91 (positive aspects); 40.19 (objective burden); and 41.97 (subjective burden; Table 3 and online Supplementary Section 4). For wellbeing outcomes, the pooled standardised means were: 14.64 (negative affect); 55.64 (positive affect); and 69.39 (eudemonic wellbeing; Table 3 and online Supplementary Section 5).

#### Certainty of evidence

Online Supplementary Table S14 provides a summary of certainty of evidence rating for each quantitative outcome of interest. Certainty of evidence ratings was determined in accordance with GRADE (Guyatt et al., 2011).

### Qualitative findings

**Figure 5.**
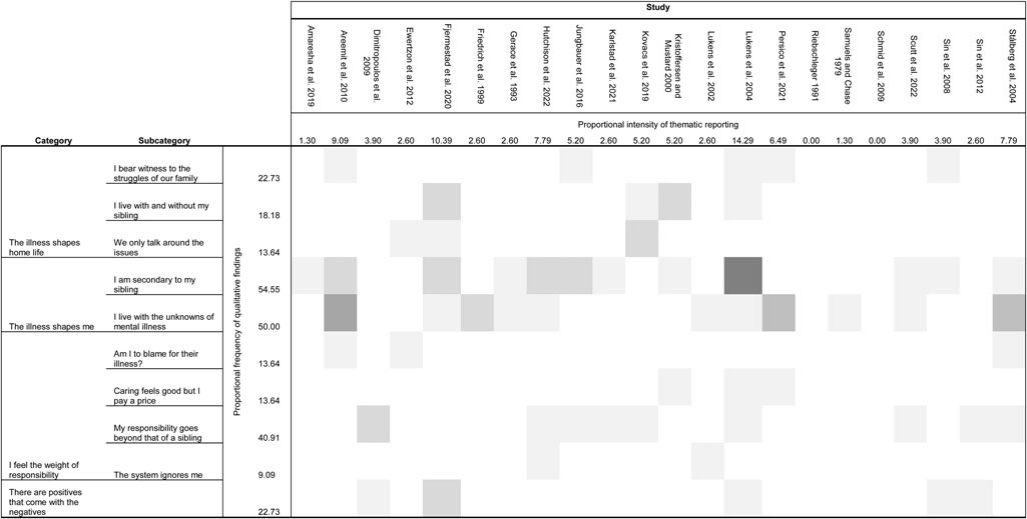
Resulting organisational structure, the proportional frequency of extracted data from included publications, and the proportional frequency of primary data comprising each subcategory. *Note.* Figure and method of analysis used to produce figure were adapted from “Positive and negative impacts of schizophrenia on family caregivers: a systematic review and qualitative meta-summary” by Shiraishi and Reilly (2019); and by Sandelowski and Barroso (2007). The proportional intensity of thematic reporting (PI-TR) was calculated by dividing the number of included (sub)themes from one study by the total number of included (sub)themes. The PI-TR represents the contribution of each study to the overall synthesis. Similarly, the proportional frequency of qualitative findings (PF-QF) was calculated by dividing the number of studies that reported on a specific subtheme by the total number of studies eligible for inclusion. The PF-QF represents the extent to which each subtheme of the current synthesis features in the qualitative studies included in this review. The figure also provides a heat map illustrating the number of extracted (sub)themes from each included study that contributed to each subtheme of the resultant thematic structure.

The content of qualitative data was organised into a structure comprising three levels (Fig. 5). We used the term ‘category’ to denote the highest level. Four categories emerged from our analysis. Our four categories encompassed nine ‘subcategories’, which constitute the second level of our structure. We used the term ‘component’ to denote our third and final level. Four components emerged from our analysis, each of which highlights a specific aspect of a subcategory.

#### Category 1: The illness shapes home life

Almost half (40.91%) of included qualitative studies suggested that siblings’ experience of home life was adversely affected by MI. Three subcategories emerged in characterising these impacts.

**Subcategory 1.1: I bear witness to the struggles of our family.** Siblings were highly aware of the challenges faced by their family members. For example, siblings observed arguments between parents and/or parent(s) and their sibling with MI. Siblings also noticed numerous failed attempts made by their parents to help their sibling with MI. Such experiences resulted in siblings feeling sad, helpless, frustrated, and/or angry.

I felt helpless, but also aggressive because she was so indifferent. Our parents sat there, crying, and she just sat there and said, ‘just leave me alone!’ I was so angry, and I screamed at her. One time I even resorted to violence. (Jungbauer, Heibach, & Urban, 2016, pg. 81–2)

One study revealed that some parents reach the point of giving up, leaving siblings feeling further disappointed and isolated. All primary data included in this subcategory were classified under ‘burden’.

**Subcategory 1.2: I live with and without my sibling.** Siblings described experiencing ongoing grief due to the loss of the connection they shared with their sibling prior to illness onset. Participants described their sibling with MI as both present and not present, which subsequently impacted their broader experience of home life. For some, their grief was compounded by the difficulties that resulted from caring for their sibling which remained in the absence of connection to their sibling. Others did not allow themselves to grieve as this was associated with a deep loss of hope: ‘it is the same as if you have pronounced the person dead.’ (Kristoffersen & Mustard, 2000, pg. 25). Primary data in this subcategory were classified as ‘burden’ and/or ‘distress’.

**Subcategory 1.3: We only talk around the issues.** Siblings shared that communication became limited and superficial in their families. This occurred in various relationships and for various reasons. For example, some parents intentionally did not discuss illness-related issues with the siblings of their child with MI. In some families, members talked often among themselves but not with the individual with MI. In others, siblings struggled to talk openly about their own challenges. Across studies, siblings felt that more open communication would be relieving: ‘It is like having gas in your stomach. You cannot keep things that way. It leads to pain.’ (Ewertzon, Cronqvist, Lutzen, & Andershed, 2012, pg. 161). Primary data in this subcategory were all classified under ‘burden’.

#### Category 2: The illness shapes me

Over seventy per cent (72.73%) of included qualitative studies reported at least one (sub)theme relating to pervasive effects on siblings’ development of self, values, and/or understanding of the world. Two subcategories captured these impacts.

**Subcategory 2.1: I am secondary to my sibling.** Many studies (54.55%) suggested that having a sibling with MI can profoundly affect an individual's sense of self, the world, and their relationships. In some instances, this occurred to the extent where individuals understood themselves solely in relation to their sibling with MI. One sibling questioned, ‘Do I exist in this world, really, or is it just her?’ (Fjermestad, Ro, Espeland, Halvorsen, & Halvorsen, 2020, pg. 87). Siblings reported feeling that their needs were less important than others, particularly their sibling with MI. Contributing to this experience, siblings reported that extended family often focused on how their sibling with MI was doing and rarely asked about them. Primary data in this subcategory were classified as ‘burden’ and/or ‘wellbeing’. Two specific components emerged within this subcategory.

***Component 2.1.1: I compensate for their illness.*** Siblings described responding to the struggles of their families in a compensatory manner. For instance, siblings reported striving to achieve, maturing faster than their peers, or even adopting certain roles: ‘it was my job to be sane, to leave the house, to go to college’ (Lukens, Thorning, & Lohrer, 2004, pg. 495). One participant recalled evading neighbours who would complain about their sibling's symptomatic behaviours: ‘it was embarrassing and uncomfortable talking to people about his condition…We were deliberately avoiding them’ (Amaresha, Venkatasubramanian, & Muralidhar, 2019, pg. 417). These compensatory responses typically resulted in siblings feeling grief, resentment, and/or burden. Primary data in this component were predominantly classified under ‘burden’.

***Component 2.1.2: My emotional landscape is intertwined with their illness.*** Siblings described an inextricable link between their day-to-day emotions and the health of their sibling with MI. For example, an inescapable consequence of loving a sibling with MI was to feel sorrow when they suffer. As one participant highlighted, the emotional impact of a sibling's ill-health can be severe:

I couldn't sleep in the night… I would be crying day and night, I think because I used to go there [hospital] and the things she was saying…. I had so many problems, I couldn't eat or sleep, and that just affected me badly. (Sin, Moone, & Harris, 2008, pg. 35)

Siblings also described ongoing guilt and/or internal conflict in deciding how much caregiving they ought to provide. Primary data in this component were predominantly classified under ‘distress’.

**Subcategory 2.2: I live with the unknowns of mental illness.** Siblings reported struggling with unanswered questions about the illness. For example, participants attempted to make sense of why their sibling developed a MI: ‘We often ask ourselves ‘why’, but there is no particular reason… We try to understand, but we don't know anything, so we feel ill at ease’ (Persico, Grandclerc, Giraud, Moro, & Blanchet, 2021, pg. 5). As a result, siblings described experiencing grief coupled with a sense of injustice and/or fear that they or their children may also become unwell. Some siblings lived with persistent fear due to the unpredictability of their sibling's illness, while others experienced confusion about the cause of unusual symptoms, hypervigilance to symptoms of MI in others, uncertainty about their own mental health, and/or powerlessness in not knowing how to help. Within this subcategory, primary data were mostly classified under ‘distress’. Two specific components emerged in characterising this subcategory.

***Component 2.2.1: I live with others’ confusion.*** An integral aspect of living with the unknowns of MI was living with other people's misunderstandings, e.g. stigma. Siblings reported that this difficulty was most pronounced during illness onset. Importantly, one study found that stigma can extend to siblings’ interactions with support services. As one sibling described:

…The hardest part was that people do not understand it is an illness. When he was violent, police would come to our house and handcuff him… then we would go to the hospital and see him shackled to a bed because he was violent. (Friedrich, Lively, & Buckwalter, 1999, pg., 16)

All primary data in this component were classified under ‘burden’.

***Component 2.2.2: What belongs to their illness?*** Participants struggled to differentiate their sibling from symptoms of the illness. As a result, participants struggled to connect with their sibling. As one sibling described:

It's hard to determine what is the illness and what is the person… What should I get mad about and what should I just let slide? … How do I deal with this in a positive way…? (Lukens, Thorning, & Lohrer, 2002, pg. 358)

Importantly, participants who attributed control over illness-related behaviours to their sibling with MI experienced frustration and anger towards their sibling. In some instances, individuals felt envious of the perceived advantages their sibling received due to their illness. Primary data in this component were mostly classified as ‘distress’.

#### Category 3: I feel the weight of responsibility

Over sixty per cent (63.64%) of included studies reported on siblings’ experience of burdensome responsibilities. Four subcategories emerged in the characterisation of this experience.

**Subcategory 3.1: Am I to blame for their illness?** Individuals questioned whether they are in some way responsible for their sibling's illness: ‘I thought and wondered if I had anything to do with why it's like this and wondered if anyone in the family caused this illness’ (Stålberg, Ekerwald, & Hultman, 2004, pg. 449). This manifested in feelings of guilt over things they had done and/or fears that they may do something to cause an escalation in their sibling's symptoms. One study indicated that, with time, siblings can move past these feelings by accepting that their actions are unrelated to their sibling's illness. Primary data in this subcategory were mixed with classifications of ‘distress’, ‘burden’, and ‘wellbeing’.

**Subcategory 3.2: Caring feels good but I pay a price.** For many siblings, the positive and negative aspects of caregiving were interconnected: ‘it makes us feel good inside, but at the same time, it destroys us’ (Persico et al., 2021, pg. 6). Siblings revealed that providing care requires sustaining hope that one's efforts are helpful and, as an unavoidable consequence, the experience of despair when their sibling relapses. Participants acknowledged that, because of having a sibling with MI, they developed positive traits such as a deep compassion for others. This was seen as ‘both a curse and a blessing’ as such traits drew others to seek out their support as well (Lukens et al., 2004, pg. 494). Primary data in this subcategory were classified as ‘burden’ and/or ‘wellbeing’.

**Subcategory 3.3: My responsibility goes beyond that of a sibling.** Siblings described needing to step into parental and/or adult roles: ‘dad was upset, my sister was upset, and I was the one comforting them’ (Karlstad, Moe, Wattum, Adelsten Stokland, & Brinchmann, 2021, pg. 5). For some, their relationship with their sibling with MI bore a closer resemblance to a parent-child relationship than a sibship. In adopting adult roles, siblings reported acting as a therapist, mediator, and/or social worker for other family members.

‘I was ‘this’ with that person, ‘this’ with the other person, ‘this’ with my brother, a lot of fragmentation and exerted energy. The family bonds and relationships get confusing because my parents have secret conversations about my brother with me. There is a struggle because I'm not their social worker [expressing frustration about being expected to fill this role within the family]. I end up playing therapist for everyone.’ (Lukens et al., 2004, pg. 495).

In response to these roles, siblings described feelings of stress, frustration, guilt, fear, and inner conflict. Primary data in this subcategory were mostly classified as ‘burden’.

**Subcategory 3.4: The system ignores me.** Two studies indicated that siblings encounter difficulties when engaging with mental health services, thereby increasing the challenges of their caregiving role. The experience involved being unheard, excluded, devalued, and/or dismissed: ‘yeah, I went, I always went to the family therapies, and it was pretty shocking because they didn't really know what to do with me’ (Hutchison et al., 2022). This data was classified as ‘burden’.

#### Category 4: There are positives that come with the negatives

Almost a quarter (22.73%) of included qualitative studies suggested that siblings recognise there are positive aspects to having a sibling with MI despite the difficulties. Some siblings reported that their family grew closer. As one sibling recounted:

It has affected … [the family] but in a way, like, we've become a really, really close family as well. Very close, so, at the same time, it's a good experience, in getting us all close. (Sin, Moone, Harris, Scully, & Wellman, 2012, pg. 56)

Others reported developing a stronger connection with their sibling with MI or new ways of seeing life that prioritised balance, happiness, compassion, meaning and/or gratitude. Primary data in this category were mostly classified as ‘wellbeing’.

## Discussion

This is the first study to systematically review quantitative and qualitative literature on distress, burden, and wellbeing in siblings of people with MI. We examined the extent of depressive symptoms, anxiety symptoms, burden, and wellbeing in siblings; as well as individuals’ experience of having a sibling with MI. Sixty-two studies involving 3744 participants from 24 countries were reviewed.

### Summary of main findings

#### Distress

The meta-analytic findings of this review suggest that siblings’ depressive symptoms fall in the mild range of the HAM-D and their anxiety symptoms fall in the minimal range of the HAM-A. Our qualitative analysis indicated that siblings may experience particularly elevated distress during specific illness stages. For example, siblings may experience heightened distress during illness onset because of needing to reconsider how they understand and relate to their sibling with MI. For siblings, this process can involve considerable grief, loss, and confusion. Lack of clarity about MI, which is likely to be more pronounced during illness onset, emerged as another contributor to sibling distress in our qualitative analysis. An important aspect of this lack of clarity was siblings’ uncertainty about how much control individuals have over their illness-related behaviours. This finding is consistent with prior reviews linking attributions of control over illness-related behaviours with greater expressed emotion (Barrowclough & Hooley, 2003) and caregiver distress (Jansen, Gleeson, & Cotton, 2015) in family members of people with MI. Finally, our qualitative findings suggest that individuals experience greater distress when their sibling with MI is acutely unwell. This finding is consistent with prior reviews associating illness severity with adverse psychological characteristics in family members of people with MI (Fekadu et al., 2019; Steele, Maruyama, & Galynker, 2010).

Our moderator analyses shed further light on factors relating to siblings’ experience of distress. Regardless of MI category, siblings reported experiencing moderate depressive symptoms while clinicians evaluate siblings as experiencing mild symptoms. Discrepancies between self-reported and clinician-rated depressive symptoms have been identified in prior literature and may be the result of variations in instrument content and/or personality factors (Enns, Larsen, & Cox, 2000; Uher et al., 2012). Given that siblings remain largely overlooked in research and clinical settings, a further possible explanation for this difference is that researchers and/or clinicians may underestimate the difficulties experienced by siblings. Of note, regardless of instrument rater, siblings of people with a schizophrenia spectrum disorder experience moderate depressive symptoms while siblings of people with other types of MI experience minimal depressive symptoms. There are many possible causes of this difference, e.g. siblings of people with a schizophrenia spectrum disorder may experience greater adversities relating to symptoms of psychosis, social exclusion, and/or stigmatisation. However, there are no prior reviews comparing sibling subgroups to shed further light on this difference. Our finding that male siblings experience greater depressive symptoms, while potentially interesting due to its discrepancy with previous reviews (Baronet, 1999; Fekadu et al., 2019; Jayasinghe et al., 2022; Shivers & Textoris, 2021), should be interpreted with caution. Gender was no longer a significant covariate after removal of two outlying studies, which both included siblings of people with a schizophrenia spectrum disorder. Thus, it is possible that our initial finding was driven by category of MI rather than gender. Anxiety symptoms for siblings living in Asia and Oceania fell in the mild range while siblings living in Europe and the Americas experienced minimal anxiety symptoms. Importantly, this analysis included only 16 publications and may, therefore, not be generalisable. If our findings were to be replicated, one possible explanation for this difference is that Asia and Oceania may include more collectivist cultures, which tend to promote greater willingness in individuals to take up informal caregiving duties (Zarzycki, Morrison, Bei, & Seddon, 2023). Cultural values influence the extent and nature of the stigmatisation of mental illness (Abdullah & Brown, 2011). Such differences might contribute to the difference seen between regions; however, the specific role of culture in individuals’ experience of mental illness stigma is likely to be nuanced and requires further clarification (Abdullah & Brown, 2011). Finally, our moderator analyses indicate that siblings’ anxiety symptoms decrease with their age. This stands in contrast with the findings of our prior review, which suggests sibling age is unrelated to the severity of their anxiety symptoms (Jayasinghe et al., 2022). One possibility for this inconsistency is that siblings’ anxiety is moderated by factors that we were unable to assess such as category of MI, stage of illness, illness severity, level of parental involvement, time spent with a sibling with an MI, and/or extent of caregiving.

#### Burden

Our meta-analytic findings across burden outcomes, i.e. overall burden, negative aspects of burden, objective, and subjective burden, were similar in that each outcome approached the mid-point of the spectrum. Siblings’ experience of positive aspects of burden fell at the middle of the spectrum. In concord with these results, a major finding of the qualitative synthesis is that siblings experience a considerable weight of responsibility. Qualitative findings revealed several factors that may contribute to siblings’ burden: for example, the need to compensate for a sibling's MI; uncertainty about whether one's actions have and/or will contribute to a sibling's illness; or the undertaking of adult roles in attempting to help one's family. Siblings who struggle with challenges in their home life – particularly constrained communication – also experience elevated burden.

#### Wellbeing

In our meta-analytic findings, sibling's positive affect appeared to be higher than their negative affect and their eudemonic wellbeing approached the higher end of the spectrum. Qualitative findings suggest that siblings’ sense of wellbeing is closely related to the nature of their caregiving role. Siblings who internalise the belief that their needs are less important than others may experience reduced wellbeing. However, when siblings acknowledge their personal gains and balance caring for themselves and others, their caregiving role can be a meaningful and enriching part of their life.

### Completeness and generalisability of evidence

We identified several important factors limiting the meaningful development of this field of research. First, the reviewed studies examined various quantitative constructs relating to burden, thereby precluding an overall synthesis of findings on burden. Second, there are currently no quantitative instruments designed to capture the unique experience of siblings. As such, the available data does not capture important aspects such as burden resulting from a need to compensate for the impacts of a sibling's MI. Third, many qualitative studies reported on broad-ranging (sub)themes that captured numerous interrelated concepts. For example, one theme titled ‘continuum of illness impacts’ comprised severe alcoholism in parents, the need to mediate between one's family and health care services, social isolation, as well as increased tolerance for peoples’ differences (Gerace, Camilleri, & Ayres, 1993). This hinders meaningful meta-synthesis of findings as important aspects of siblings’ experiences are necessarily lost in the distillation of such data. Fourth, some studies relied on sibling attestation (10.94%) to verify the presence of a MI while others did not report how diagnosis was confirmed (43.75%) thereby impeding an assessment of the reliability and generalisability of findings. Finally, there are no validated cut-off scores for the measures of burden and wellbeing. So, we are currently unable to consider subgroups of siblings who experience notably elevated burden and/or reduced wellbeing. This represents a substantial limitation to the effective development and use of mental health resources.

As a result of our review, several gaps in the current literature emerged as important areas for future research. First, there is limited quantitative data on siblings’ wellbeing. This is problematic as strong evidence suggests mental illness and wellbeing are related but distinct continuums, such that the absence of mental illness does not indicate greater wellbeing (Westerhof & Keyes, 2010). An understanding of wellbeing is crucial in providing support to siblings of people with MI: a cohort who, although they may not be psychiatrically unwell, are vulnerable to reduced wellbeing. Second, data predominantly related to siblings of people with a schizophrenia spectrum, eating, or affective disorder. So, there is a lack of information about siblings of people with other types of MIs such as personality or trauma-related disorders. Third, only two qualitative studies reported a (sub)theme relating to siblings’ interactions with mental health services. This may be due to inadequate enquiry because of researchers underestimating the importance of such interactions in the lives of siblings. Fourth, there is currently no available data on distress, burden, or wellbeing in individuals who have a deceased sibling who experienced MI. This is concerning given that people with MI have considerably reduced life expectancy largely due to increased rates of physical illness and suicide (Walker, McGee, & Druss, 2015). Fifth, few studies reported on illness characteristics (such as stage or severity of illness) precluding their inclusion in our moderator analyses. This is surprising given that illness-related variables have been consistently linked with psychological difficulties in family members of people with MI (Baronet, 1999; Saunders, 2003; Steele et al., 2010). Sixth, no studies provided data beyond binary gender constructs precluding any analysis of the experience of those who do not identify with these classifications. Seventh, only one included study was conducted in a low-income status country. This is notable as family members, including siblings, in developing nations are likely to have higher caregiving responsibilities and less access to professional support due to under-resourced mental health sectors (Jacob et al., 2007). Finally, it was beyond the scope of this study to examine differences in siblings’ experiences based on the category of mental illness in the qualitative analysis. Investigation of such nuances is an important direction for future research given that qualitative findings have linked specific illness features with impacts on siblings. For example, siblings of people with an eating disorder have highlighted declines in family dynamics at mealtimes (Fjermestad et al., 2020), while siblings of people with schizophrenia have reported increased distress in relation to positive symptoms in their family member (Sin et al., 2012).

### Strengths and limitations of the review

The novel sequential explanatory design employed in the current review represents a major strength. Quantitative data on distress, burden, and wellbeing were identified according to well-recognised operationalisations and synthesised using meta-analytic techniques. Qualitative data were synthesised using a content meta-analytic approach and findings were subsequently used to contextualise and enhance our understanding of quantitative results. In so doing, we have provided a comprehensive analysis of well-recognised psychological characteristics in siblings.

This review has four main limitations. First, there was insufficient data to investigate sources of heterogeneity in our outcomes of anxiety symptoms, burden, and wellbeing. In our analysis of anxiety symptoms, there was evidence of small study bias that could not be assessed further. Additionally, our meta-analyses relating to burden and wellbeing comprised relatively small samples and the generalisability of these findings remains unclear. Second, reviewed studies included only cross-sectional data. As a result, each quantitative outcome of interest received a ‘very low’ certainty of evidence rating according to the GRADE assessment framework, and no conclusions can be drawn about changes across developmental phases or stages of illness. Third, this review did not include studies published in other languages. We might, therefore, have introduced a bias toward data available to English-speaking authors. Fourth, only studies with ⩾10 siblings were eligible for inclusion, which resulted in the exclusion of nine potentially eligible reports that predominantly provided qualitative literature (online Supplementary Table S5). This too might have introduced bias into the findings.

### Clinical implications

The findings of this review underscore the need to consider siblings in interventions that seek to support caregivers and/or families of people with MI. Our qualitative findings revealed several important areas for intervention. For instance, siblings may benefit from support in the development of a new understanding of their sibling as an individual with MI. Siblings may also benefit from assistance in identifying ways to help their family without compromising their own wellbeing, and to recognise personal gains that may have emerged from their caregiving role. Increasing open communication within families emerged as a further area of importance for siblings. Information about MI is likely to benefit siblings. Crucially, information about the cause(s), symptoms, and prognosis of their sibling's MI, as well as information about the level of control individuals have over illness-related behaviours is recommended.

Our qualitative findings suggest that some parents, particularly those with strained resources, underestimate difficulties faced by siblings and that some siblings may themselves minimise the extent of their difficulties to prevent further burden on their family. In such instances, it is critical for clinicians to acknowledge the potential struggles of siblings to avoid further reinforcing the notion that the needs of siblings are secondary to the needs of a child with MI. This point warrants further emphasis given that our moderator analyses indicate siblings’ self-reported psychological challenges are greater than those reflected in clinician-rated instruments.

## Conclusion

The findings of this systematic review and meta-analysis underscore the psychological vulnerability of siblings of people with MI to distress and burden, as well as reduced wellbeing. Several areas were identified as potential intervention targets including communication within families, siblings’ knowledge of MI, recognition of personal gains resulting from having a sibling with MI, and balancing siblings’ needs with those of other family members. To support the development of meaningful, evidence-based interventions for this important cohort, further research is required to clarify the mechanisms underlying siblings’ experience of distress, burden, and wellbeing.

## Supporting information

Jayasinghe et al. supplementary material Jayasinghe et al. supplementary material

## Supplementary material

The supplementary material for this article can be found at https://doi.org/10.1017/S0033291723001733.
