# Supplementary material for: Distress, burden, and wellbeing in siblings of people with mental illness: a mixed studies systematic review and meta-analysis
Source: Psychol Med. 2023 Jul 25;53(15):6945–64. doi: 10.1017/S0033291723001733 (PMC10951414; doi:10.1017/S0033291723001733)
Supplement: Jayasinghe et al. supplementary material [file S0033291723001733sup001.docx]

**Supplementary Material**

**Distress, burden, and wellbeing in siblings of people with mental illness: A mixed studies systematic review and meta-analysis**

Anuradhi Jayasinghe, Anna Wrobel, Kate Filia, Linda K. Byrne, Glenn Melvin, Sean Murrihy, Carl Moller, Lesley Berk, Michael Berk, Sue Cotton

Table of Contents

[Section One: Main Tables 2](#_Toc119308612)

[**Supplementary Table 1.** PRISMA 2020 item checklist 3](#_Toc119308613)

[**Supplementary Table 2.** PRISMA 2020 for Abstract checklist 5](#_Toc119308614)

[**Supplementary Table 3.** List of search terms by database 6](#_Toc119308615)

[**Supplementary Table 4.** Eligibility criteria for the broader and current review 9](#_Toc119308616)

[**Supplementary Table 5.** List of reports meeting most inclusion criteria and reason(s) for their exclusion 10](#_Toc119308617)

[**Supplementary Table 6.** Additional methodological details for quantitative and qualitative analysis 11](#_Toc119308618)

[**Supplementary Table 7.** Results of qualitative data ratings and classifications of data according to established concepts of distress, burden, and wellbeing 12](#_Toc119308619)

[**Supplementary Table 8.** Additional characteristics of siblings of people with mental illness in reviewed studies 16](#_Toc119308620)

[**Supplementary Table 9.** Demographic characteristics of siblings with a mental illness in reviewed studies 20](#_Toc119308621)

[**Supplementary Table 10.** Illness-related characteristics for siblings with a mental illness and eligibility criteria for reviewed studies 23](#_Toc119308622)

[**Supplementary Table 11.** Characteristics of the sibling relationship in reviewed studies 31](#_Toc119308623)

[**Supplementary Table 12.** Quality appraisal of reviewed studies providing quantitative data 33](#_Toc119308624)

[**Supplementary Table 13.** Quality appraisal of reviewed studies providing qualitative data 34](#_Toc119308625)

[**Supplementary Table 14.** Summary of findings according to Grading of Recommendation, Assessment, Development and Evaluation (GRADE) guidelines 35](#_Toc119308626)

[**Supplementary Table 15.** Materials available on request 36](#_Toc119308627)

[Section Two: Quantitative Results for Depressive Symptoms 37](#_Toc119308628)

[Section Three: Quantitative Results for Anxiety Symptoms 48](#_Toc119308629)

[Section Four: Quantitative Results for Burden 52](#_Toc119308630)

[Section Five: Quantitative Results for Wellbeing 60](#_Toc119308631)

[Section Six: References 64](#_Toc119308632)

# Section One: Main Tables

## **Supplementary Table 1.** PRISMA 2020 item checklist

| **Section and Topic** | **Item #** | **Checklist item** | **Page** |
| --- | --- | --- | --- |
| *Title* |  |  |  |
| Title | 1 | Identify the report as a systematic review. | pg. 1 |
| *Abstract* |  |  |  |
| Abstract | 2 | See PRISMA 2020 for Abstracts checklist. | Supplementary Table 2 |
| *Introduction* |  |  |  |
| Rationale | 3 | Describe the rationale for the review in the context of existing knowledge. | pp. 4-5 |
| Objectives | 4 | Provide an explicit statement of the objective(s) or question(s) the review addresses. | pg. 5 |
| *Methods* |  |  |  |
| Eligibility criteria | 5 | Specify the inclusion and exclusion criteria for the review and how studies were grouped for the syntheses. | pg. 6 & pg. 8-9 |
| Information sources | 6 | Specify all databases, registers, websites, organisations, reference lists and other sources searched or consulted to identify studies. Specify the date when each source was last searched or consulted. | pg. 6 |
| Search strategy | 7 | Present the full search strategies for all databases, registers and websites, including any filters and limits used. | pg. 6 & Supplementary Table 3 |
| Selection process | 8 | Specify the methods used to decide whether a study met the inclusion criteria of the review, including how many reviewers screened each record and each report retrieved, whether they worked independently, and if applicable, details of automation tools used in the process. | pg. 6-7 |
| Data collection process | 9 | Specify the methods used to collect data from reports, including how many reviewers collected data from each report, whether they worked independently, any processes for obtaining or confirming data from study investigators, and if applicable, details of automation tools used in the process. | pg. 7 |
| Data items | 10a | List and define all outcomes for which data were sought. Specify whether all results that were compatible with each outcome domain in each study were sought (e.g. for all measures, time points, analyses), and if not, the methods used to decide which results to collect. | Supplementary Table 4 |
|  | 10b | List and define all other variables for which data were sought (e.g. participant and intervention characteristics, funding sources). Describe any assumptions made about any missing or unclear information. | pg. 7 & Supplementary Tables 4 & 6 |
| Study risk of bias assessment | 11 | Specify the methods used to assess risk of bias in the included studies, including details of the tool(s) used, how many reviewers assessed each study and whether they worked independently, and if applicable, details of automation tools used in the process. | pg. 7 |
| Effect measures | 12 | Specify for each outcome the effect measure(s) (e.g. risk ratio, mean difference) used in the synthesis or presentation of results. | pg. 8-9 & Supplementary Table 4 |
| Synthesis methods | 13a | Describe the processes used to decide which studies were eligible for each synthesis. | pg. 8-9 & Supplementary Table 4 |
|  | 13b | Describe any methods required to prepare the data for presentation or synthesis, such as handling of missing summary statistics, or data conversions. | pg. 8-9 & Supplementary Table 4 |
|  | 13c | Describe any methods used to tabulate or visually display results of individual studies and syntheses. | pg. 8-9 & Supplementary Table 4 |
|  | 13d | Describe any methods used to synthesize results and provide a rationale for the choice(s). If meta-analysis was performed, describe the model(s), method(s) to identify the presence and extent of statistical heterogeneity, and software package(s) used. | pg. 8-9 & Supplementary Table 4 |
|  | 13e | Describe any methods used to explore possible causes of heterogeneity among study results. | pg. 8-9 & Supplementary Table 4 |
|  | 13f | Describe any sensitivity analyses conducted to assess robustness of the synthesized results. | pg. 8-9 & Supplementary Table 4 |
| Reporting bias assessment | 14 | Describe any methods used to assess risk of bias due to missing results in a synthesis (arising from reporting biases). | pg. 8-9 & Supplementary Table 4 |
| Certainty assessment | 15 | Describe any methods used to assess certainty (or confidence) in the body of evidence for an outcome. | pg. 7-9 & Supplementary Table 4 |
| *Results* |  |  |  |
| Study selection | 16a | Describe the results of the search and selection process, from the number of records identified in the search to the number of studies included in the review, ideally using a flow diagram. | pg. 10 & Figure 1 |
|  | 16b | Cite studies that met many but not all inclusion criteria (‘near-misses’) and explain why they were excluded. | Supplementary Table 5 |
| Study characteristics | 17 | Cite each included study and present its characteristics. | Table 1 & 2 |
| Risk of bias in studies | 18 | Present assessments of risk of bias for each included study. | pg. 10 & Supplementary Tables 12 & 13 |
| Results of individual studies | 19 | For all outcomes, present, for each study: (a) summary statistics for each group (where appropriate) and (b) an effect estimate and its precision (e.g. confidence/credible interval), ideally using structured tables or plots. | Table 1, Figure 3, Figure 4, Supplementary Table 7 |
| Results of synthesis | 20a | For each synthesis, briefly summarise the characteristics and risk of bias among contributing studies. | Supplementary Table 15 |
|  | 20b | Present results of all statistical syntheses conducted. If meta-analysis was done, present for each the summary estimate and its precision (e.g. confidence/credible interval) and measures of statistical heterogeneity. If comparing groups, describe the direction of the effect. | pg. 11-17, Table 3 & 4, & Supplementary Sections 2-5 |
|  | 20c | Present results of all investigations of possible causes of heterogeneity among study results. | pg. 11-12, Table 3 & 4, & Supplementary Sections 2-5 |
|  | 20d | Present results of all sensitivity analyses conducted to assess the robustness of the synthesized results. | pg. 11 & Supplementary Sections 2-5 |
| Reporting biases | 21 | Present assessments of risk of bias due to missing results (arising from reporting biases) for each synthesis assessed. | Supplementary Sections 2 & 3 |
| Certainty of evidence | 22 | Present assessments of certainty (or confidence) in the body of evidence for each outcome assessed. | Supplementary Table 14 |
| *Discussion* |  |  |  |
| Discussion | 23a | Provide a general interpretation of the results in the context of other evidence. | pg. 18-20 |
|  | 23b | Discuss any limitations of the evidence included in the review. | pg. 20-21 |
|  | 23c | Discuss any limitations of the review processes used. | pg. 21-22 |
|  | 23d | Discuss implications of the results for practice, policy, and future research. | pg. 22 |
| *Other information* |  |  |  |
| Registration and protocol | 24a | Provide registration information for the review, including register name and registration number, or state that the review was not registered. | pg. 6 |
|  | 24b | Indicate where the review protocol can be accessed, or state that a protocol was not prepared. | Supplementary Table 15 |
|  | 24c | Describe and explain any amendments to information provided at registration or in the protocol. | Supplementary Table 4 |
| Support | 25 | Describe sources of financial or non-financial support for the review, and the role of the funders or sponsors in the review. | pg. 23 |
| Competing interests | 26 | Declare any competing interests of review authors. | pg. 23 |
| Availability of data, code and other materials | 27 | Report which of the following are publicly available and where they can be found: template data collection forms; data extracted from included studies; data used for all analyses; analytic code; any other materials used in the review. | Supplementary Table 15 |

## **Supplementary Table 2.** PRISMA 2020 for Abstract checklist

| **Section and Topic** | **Item #** | **Checklist item** | **Page** |
| --- | --- | --- | --- |
| *Title* |  |  |  |
| Title | 1 | Identify the report as a systematic review. | 1 |
| *Background* |  |  |  |
| Objectives | 2 | Provide an explicit statement of the main objective(s) or question(s) the review addresses. | 2 |
| *Methods* |  |  |  |
| Eligibility criteria | 3 | Specify the inclusion and exclusion criteria for the review. | 2 |
| Information sources | 4 | Specify the information sources (e.g., databases, registers) used to identify studies and the date when each was last searched. | 2 |
| Risk of bias | 5 | Specify the methods used to assess risk of bias in the included studies. |  |
| Synthesis of results | 6 | Specify the methods used to present and synthesize results. |  |
| *Results* |  |  |  |
| Included studies | 7 | Give the total number of included studies and participants and summarise relevant characteristics of studies. | 2 |
| Synthesis of results | 8 | Present results for main outcomes, preferably indicating the number of included studies and participants for each. If meta-analysis was done, report the summary estimate and confidence/credible interval. If comparing groups, indicate the direction of the effect (i.e. which group is favoured). | 2 |
| *Discussion* |  |  |  |
| Limitation of evidence | 9 | Provide a brief summary of the limitations of the evidence included in the review (e.g., study risk of bias, inconsistency and imprecision). |  |
| Interpretation | 10 | Provide a general interpretation of the results and important implications. | 2 |
| *Other* |  |  |  |
| Funding | 11 | Specify the primary source of funding for the review. |  |
| Registration | 12 | Provide the register name and registration number. |  |

##
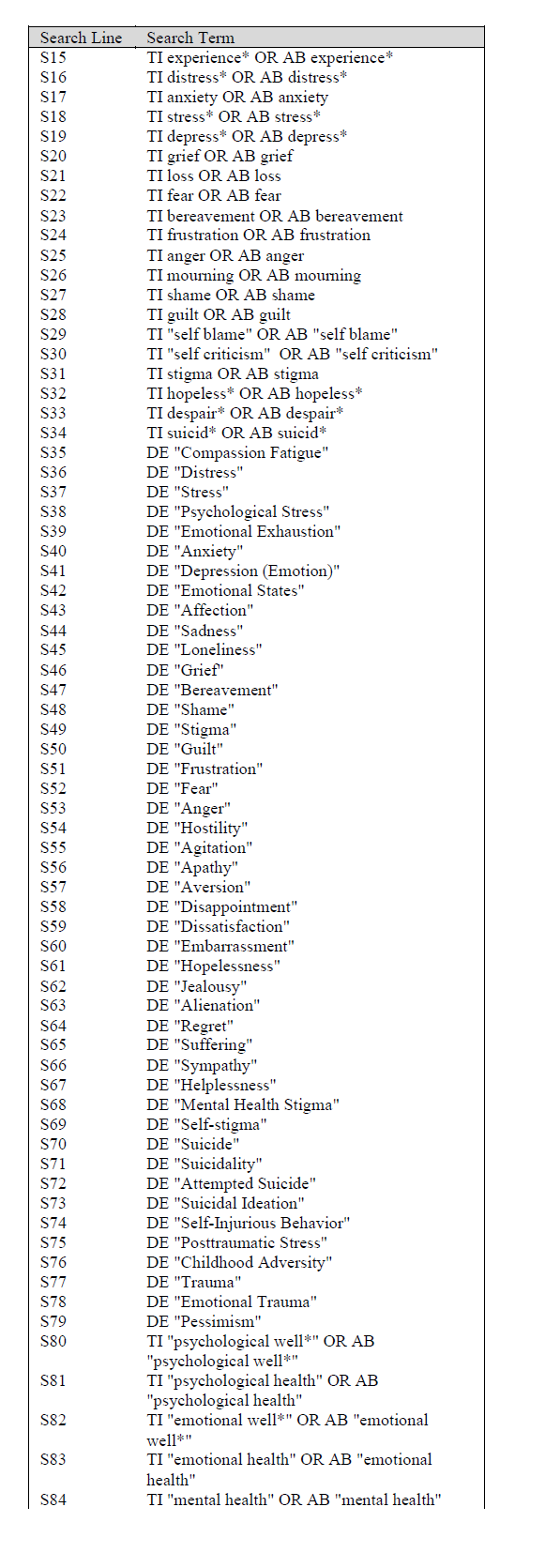

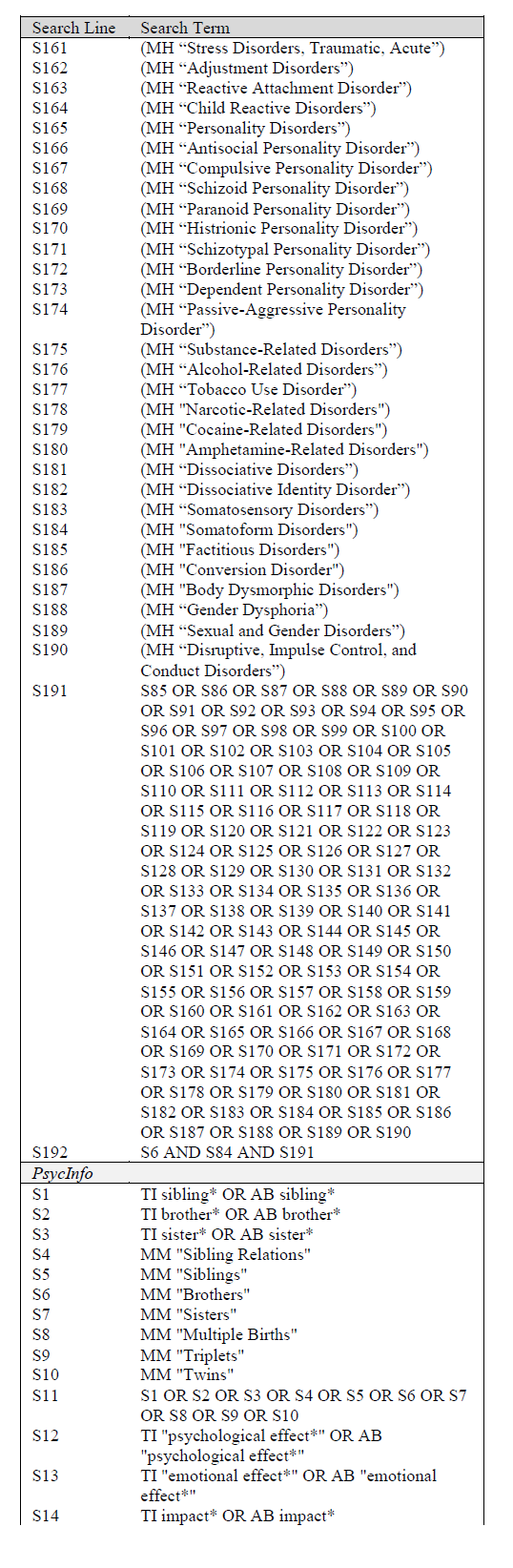

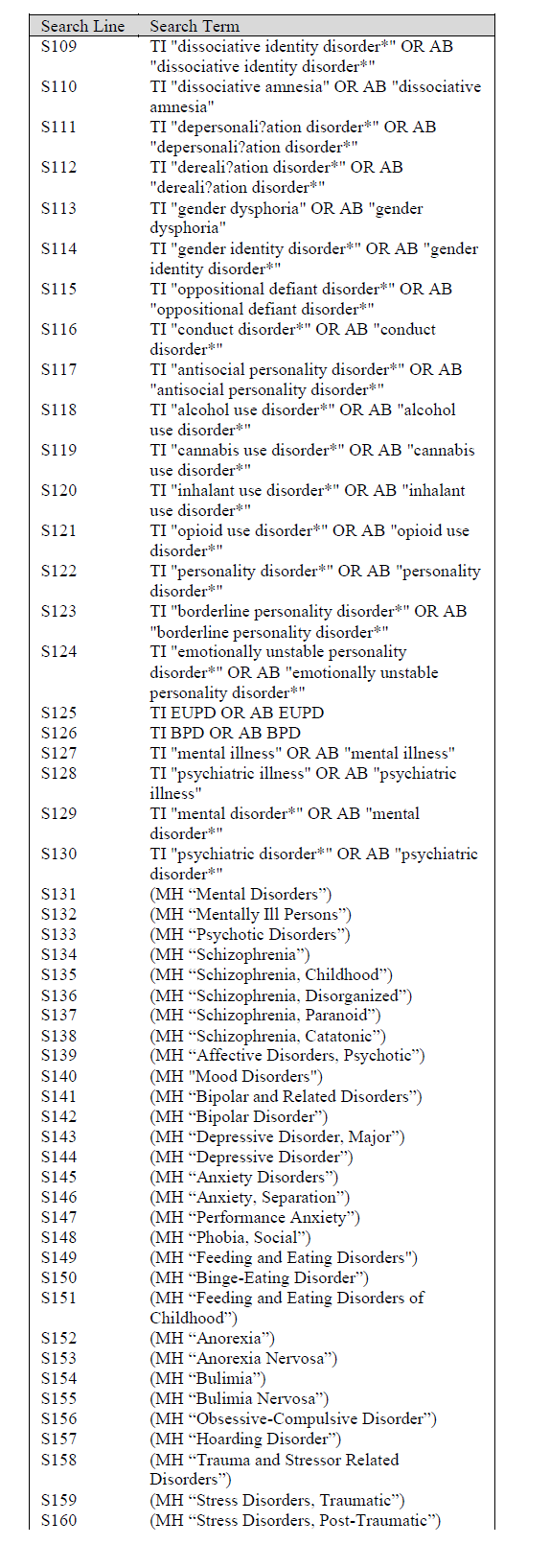

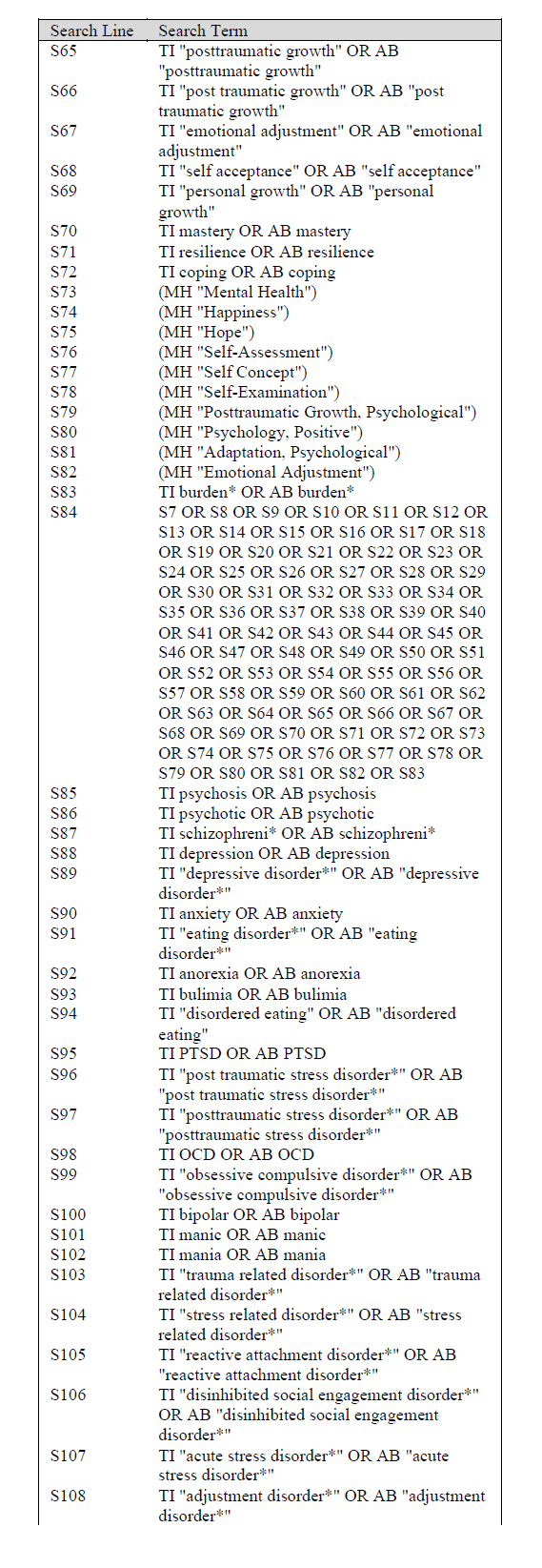

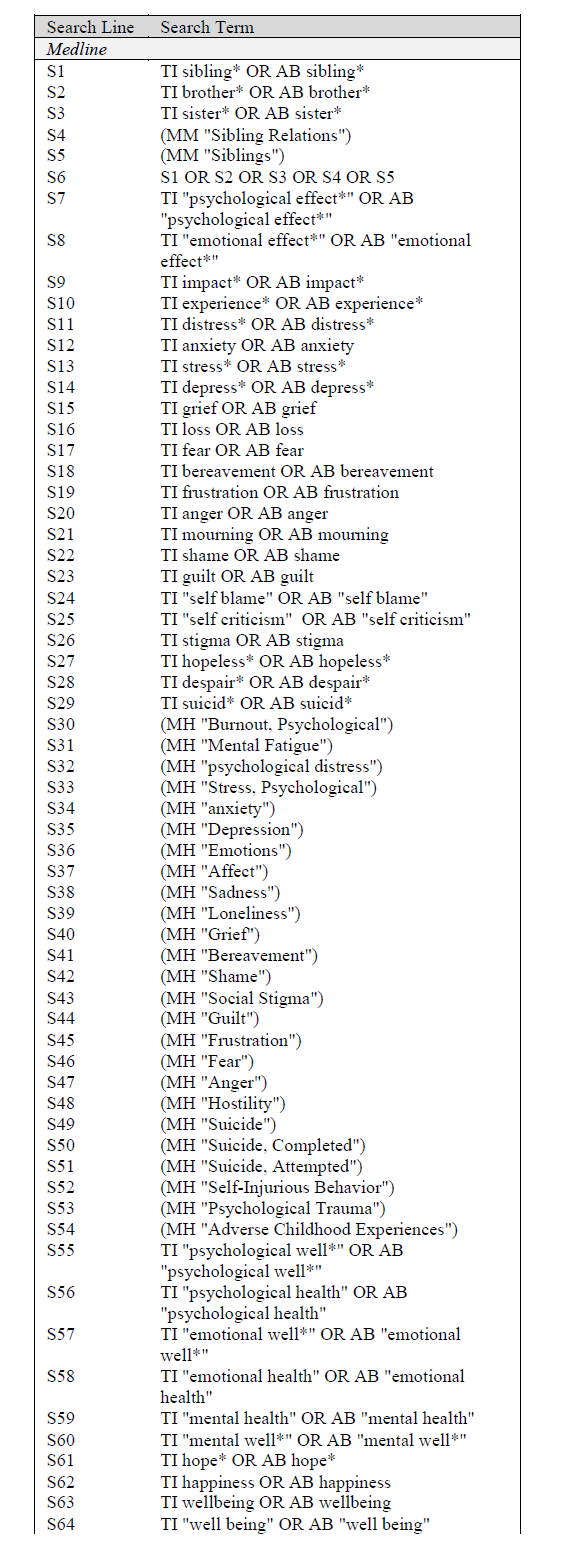
**Supplementary Table 3.** List of search terms by database


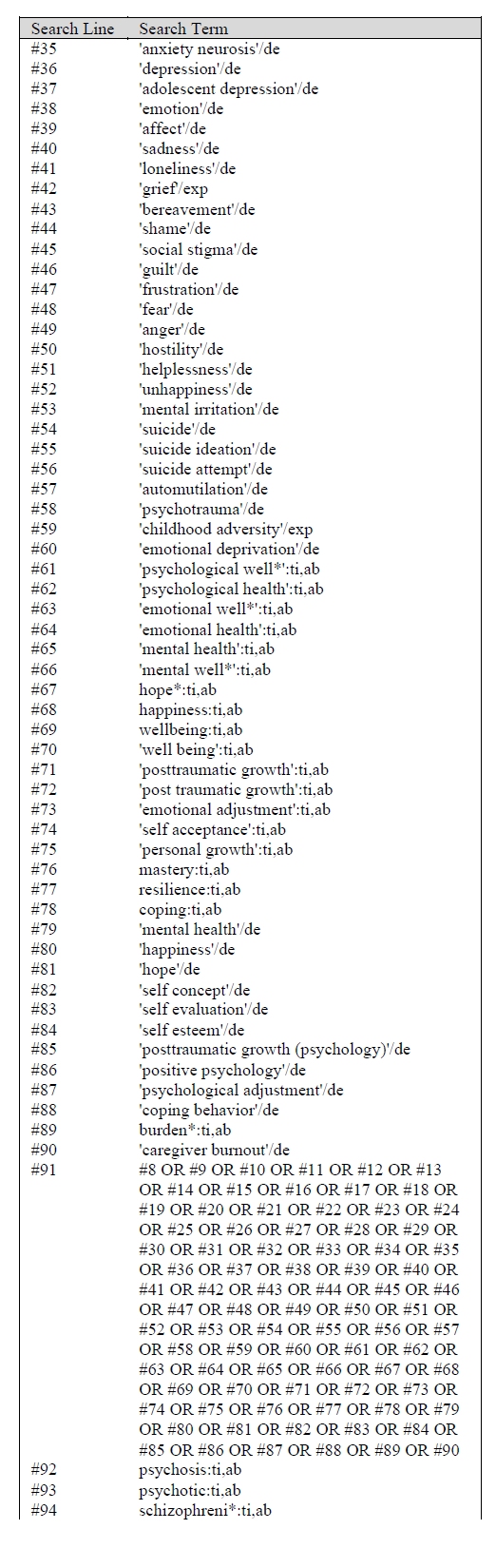

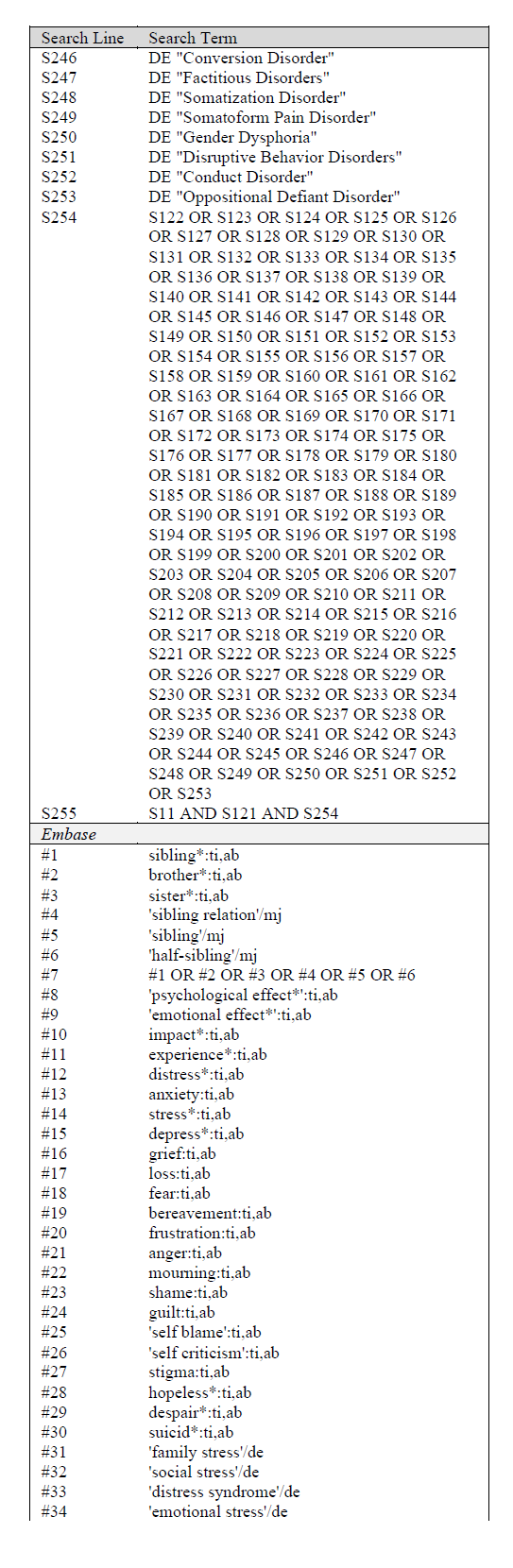

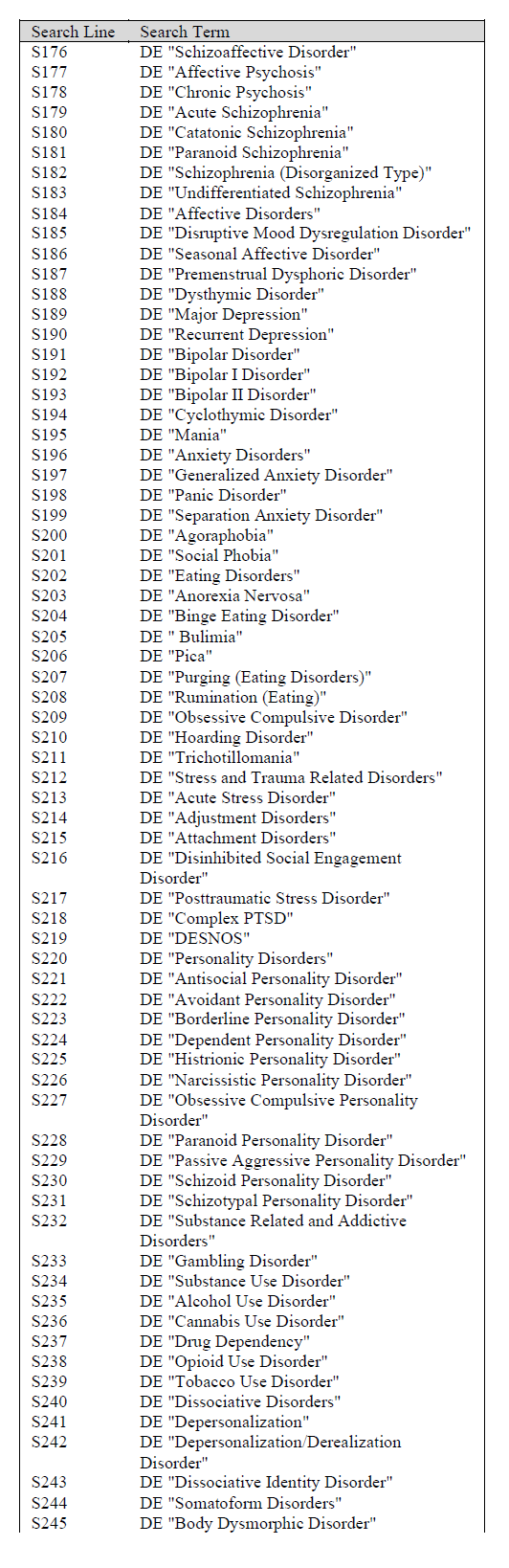

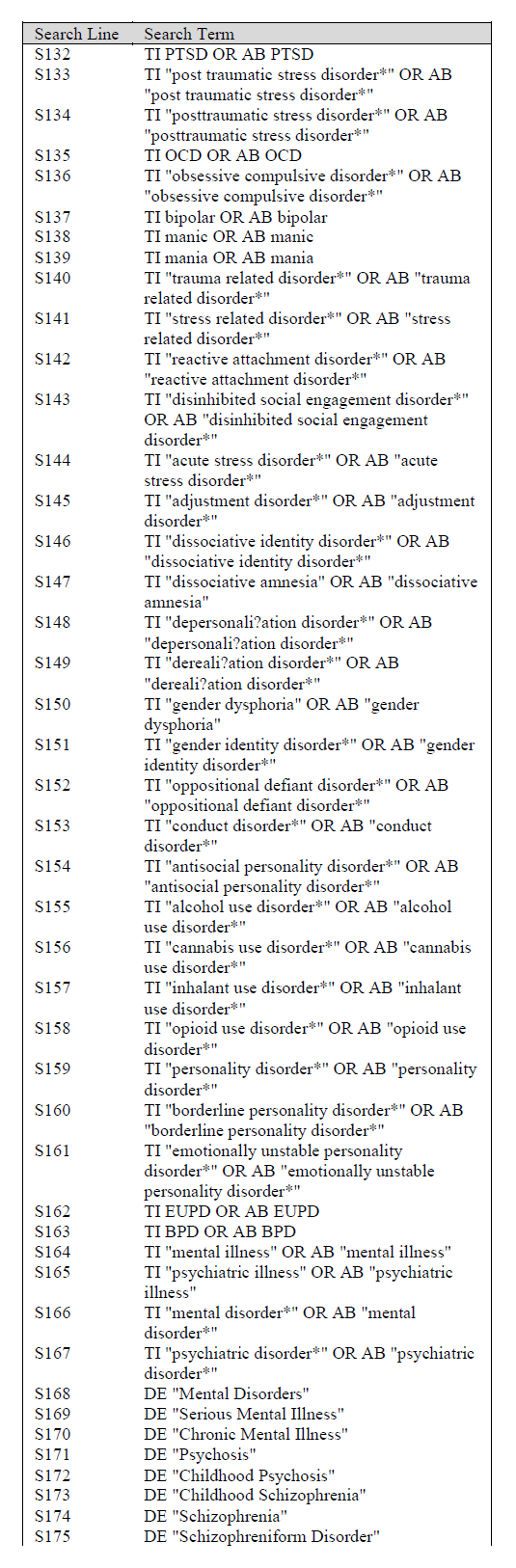

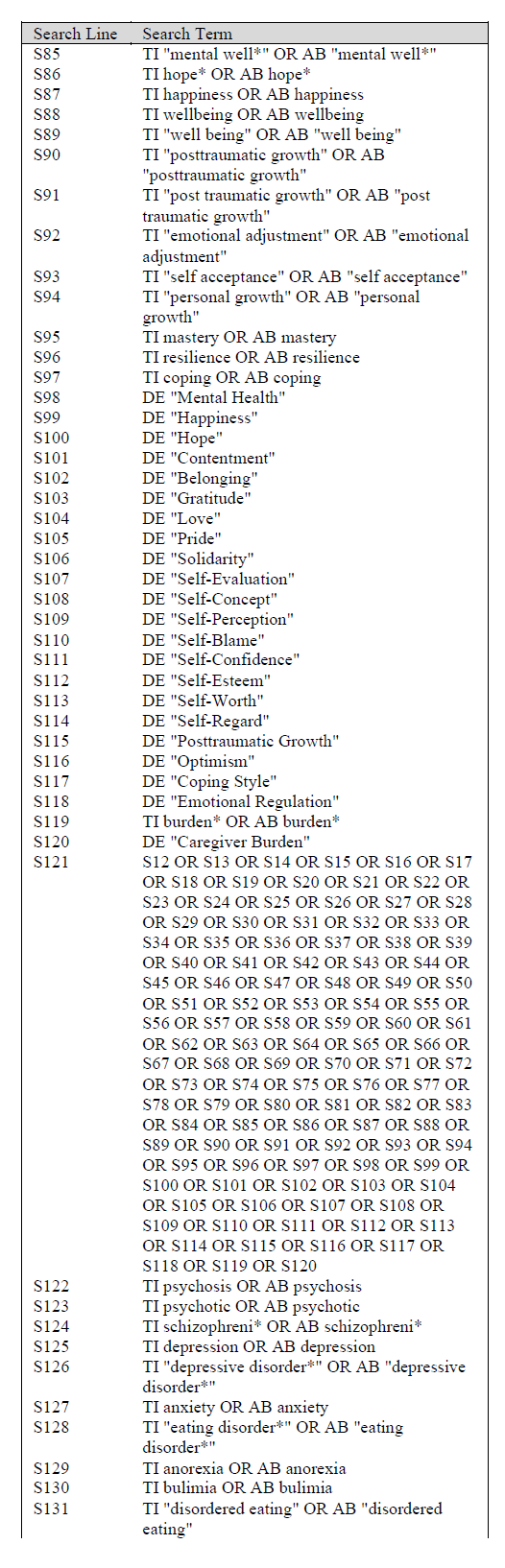


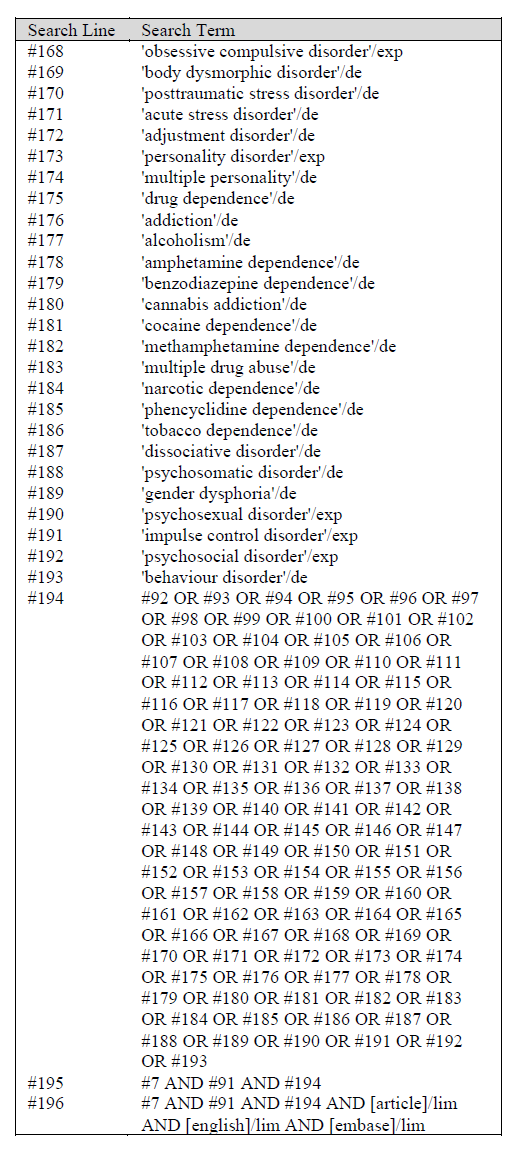

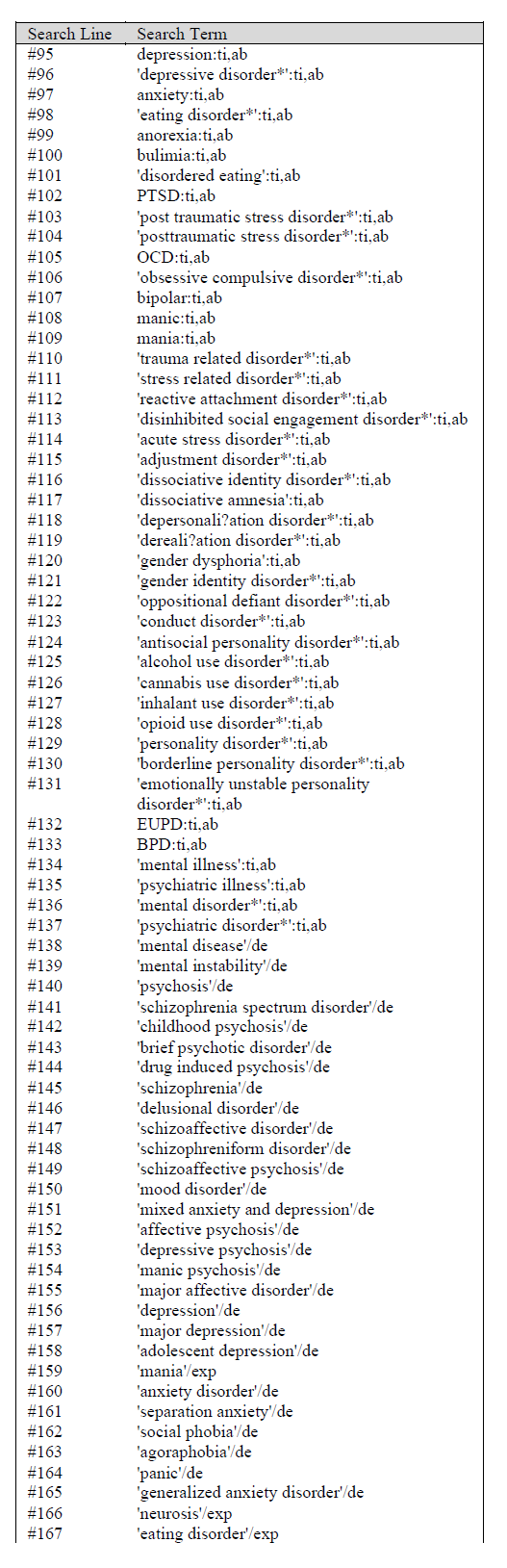


## **Supplementary Table 4.** Eligibility criteria for the broader and current review

| **Eligibility criteria for broader review** |
| --- |
| **Inclusion criteria** |
| Full text, published article, available in English. |
| Reports on original qualitative or quantitative findings. |
| Reports on participants aged ≥10 years. Given that mental illness (MI) typically emerges during adolescence (APA, 2013), only studies examining siblings of people with MI who were adolescents or adults, i.e., aged ≥ 10 years (World Health Organization, 2018), were eligible for inclusion. |
| Reports on individuals with at least one sibling with a MI.   - Siblings were required to be: biological siblings sharing two biological parents, half-siblings sharing one biological parent, stepsiblings sharing at least one parent by marriage or de-facto relationship, adoptive siblings sharing at least one parent via a government-sanctioned adoption process, or foster blah blah la siblings sharing at least one parent via a government-sanctioned foster program. No limitations were placed on the duration of the sibship or the amount of contact siblings had with one another. - Any psychiatric disorder identified in the Diagnostic and Statistical Manual of Mental Disorders (DSM) or the International Classification of Diseases (ICD) was considered to be a MI, excluding neurodevelopmental and neurocognitive disorders. Neurodevelopmental and neurocognitive disorders have considerably distinct symptom profiles to psychiatric disorders that emerge during adolescence (APA, 2013). As such, siblings of individuals with neurodevelopmental and neurocognitive disorders are likely to have different psychological experiences to siblings of people with MIs that typically emerged during adolescence or adulthood. Thus, neurodevelopmental and neurocognitive disorders were excluded from this review. The presence of an MI was identified via a structured clinical interview conforming to DSM or ICD criteria, clinical assessment, or via sibling attestation. |
| Reports on either (1) qualitative (sub)themes on the psychological experience of siblings of people with MI, (2) prevalence of distress, burden, or wellbeing in siblings of people with MI, (3) mean severity of distress, burden, or wellbeing in siblings of people with MI, or (4) an association between (a) distress, burden, or wellbeing in siblings of people with MI and (b) a demographic, sibling-related, illness-related, or relational variable. |
| **Exclusion Criteria** |
| Review or case analysis. |
| Reports on a sample of <10 participants. |
| Reports on data obtained via parent, guardian, or proband report. |
| **Other considerations** |
| Experimental and interventional studies were excluded unless they reported on relevant baseline data or provided relevant baseline data in response to a data request. |
| Studies were not required to include a comparison group. However, comparison group data was eligible for inclusion where comparison individuals were people with no sibling(s) with MI. |
| Quantitative studies reporting on a mixed sample (e.g., family members of people with mental illness) were eligible for inclusion if their study involved ≥ 10 siblings, and study authors were able to provide data on sibling participants only. No such data requests were made for qualitative studies reporting on mixed samples given that the provision of relevant qualitative data would require a new synthesis of primary data. |
| **Additional eligibility criteria for the current review** |
| **Inclusion criteria** |
| Publications reporting on the mean and standard deviation on a severity scale of depressive symptoms, anxiety symptoms, burden, or wellbeing in siblings of people with MI were eligible for inclusion in this review. |
| Publications reporting on at least one qualitative (sub)theme relating to the psychological experience of siblings of people with MI were also eligible for inclusion in the current review. |
| **Other considerations** |
| Authors were contacted to request missing data if their publication met all inclusion criteria but did not report statistics on the mean or the standard deviation on a relevant measure. Twenty-two authors were contacted. Ten authors provided missing data (Avcıoğlu, Karanci, & Soygur, 2019; Di Sarno, Napolitano, & Louzã, 2021; Diaz et al., 2021; Geller et al., 2017; Matthews et al., 2021; Panaite et al., 2019; Phoeun et al., 2022; Sletved et al., 2022; Tanaka, 2008; van Sprang et al., 2021). |
| In reports that provided statistics on two measures of the same construct (e.g., anxiety symptoms; van Sprang et al., 2021), we retained data from the measure considered most cohesive with the aims of the review and other included publications. |

## **Supplementary Table 5.** List of reports meeting most inclusion criteria and reason(s) for their exclusion

| **Study** | **Reason for exclusion** |
| --- | --- |
| Callio et al. 2016 | Comprised less than 10 sibling participants. |
| Barnable et al. 2006 | Comprised less than 10 sibling participants. |
| Begovic et al. 2017 | Overlapping sample with Kovacs et al. (2016) with no new outcomes of interest and a smaller sample size. |
| Bo et al. 2019 | Required data not provided in publication. Data request not fulfilled. |
| de Crom et al. 2021 | Overlapping sample with Boyette et al. (2013) with no new outcomes of interest and a smaller sample size. |
| de Kluiver et al. 2021 | Overlapping sample with van Sprang et al. (2021) with no new outcomes of interest. |
| Farmer et al. 2001 | Required data not provided in publication. Data request not fulfilled. |
| Garley and Johnson 1994 | Comprised less than 10 sibling participants. |
| Greenberg et al. 1997 | Required data not provided in publication. Data request not fulfilled. |
| Horwitz and Reinhard 1995 | Overlapping sample with Reinhard and Horwitz (1995). |
| Hsiao and Tsai 2014 | Required data not provided in publication. Data request not fulfilled. |
| Hsiao and Tsai 2015 | Overlapping sample with Hsiao and Tsai (2014). |
| Latzer et al. 2002 | Comprised less than 10 sibling participants. |
| Leith et al. 2018 | Total possible range on measure of burden could not be determined from the publication. A data request was made to clarify this information. However, we received no response from study authors. As such, this publication could not be included in the review. |
| Li et al. 2021 | Measure of 'burden' was based on participants' response to a single survey question. As such, this measure was not deemed cohesive with measures used by other publications (e.g., Burden Assessment Scale and Experience of Caregiving Inventory). |
| Liegghio et al. 2017 | Comprised less than 10 sibling participants. |
| Mannarini and Kleinbub 2022 | Required data not provided in publication. Data request not fulfilled. |
| Newman et al. 2011 | Comprised less than 10 sibling participants. |
| Oshodi et al. 2012 | Comprised less than 10 sibling participants. |
| Park and Lee 2017 | Comprised less than 10 sibling participants. |
| Paswan 2022 | Required data not provided in publication. Author contact details were not provided in publication and could not be obtained freely online. As such, no data request could be made. |
| Rachamim et al. 2021 | Required data not provided in publication. Data request not fulfilled. |
| Ribé et al. 2018 | Required data not provided in publication. Data request not fulfilled. |
| Scheirs et al. 2007 | Unable to determine how many sibling participants were included in this study from the publication. To best utilise limited resources, no data requests were made to clarify this information. |
| Seltzer et al. 1997 | Overlapping sample with Taylor et al. (2008) with no new outcomes of interest and a smaller sample size. |
| Shah et al. 2013 | Required data not provided in publication. Data request not fulfilled. |
| Titelman and Psyk 1991 | Comprised less than 10 sibling participants. |
| Vermeulen et al. 2019 | Overlapping sample with Boyette et al. (2013). Although Vermeulen et al. (2019) examined a larger sample size, mean and standard deviation for our outcome of interest (depressive symptoms) was not reported in Vermeulen et al. (2019). As such, we retained data from Boyette et al. (2013). |
| Vinberg et al. 2007 | Overlapping sample with Christensen et al. (2007) with no new outcomes of interest and a smaller sample size. |
| Vothknecht et al. 2013 | Overlapping sample with Boyette et al. (2013) with no new outcomes of interest and a smaller sample size. |
| Ziermans et al. 2021 | Overlapping sample with Boyette et al. (2013) with no new outcomes of interest and a smaller sample size. |

## **Supplementary Table 6.** Additional methodological details for quantitative and qualitative analysis

| Step | Further Methodological Details |
| --- | --- |
| *Data preparation* | |
| 1 | Quantitative data were tabulated according to the following a priori groupings: depressive symptoms, anxiety symptoms, burden (overall), burden (positive aspects), burden (negative aspects), burden (objective), burden (subjective), wellbeing (negative affect), wellbeing (positive affect), and wellbeing (eudemonic). |
| 2 | To allow for comparability across measures, raw scores were standardised prior to meta-analysis. Scores were standardised by calculating a fraction using the mean or standard deviation as the numerator and the possible range of scores on the relevant measure as the denominator (Bath, Deeg, & Poppelaars, 2010). The fraction was then multiplied by 100. |
| 3 | Outlying estimates of effect size for all outcomes of interest were identified via inspection of box plots using SPSS software (IBM Corp, 2020). |
| 4 | The standard deviation for depressive symptoms and anxiety symptoms was not available in one study (namely, Zauszniewski & Bekhet, 2014), and study authors were unable to provide the missing data. To retain the available data reported in this study, we pooled the standard deviations reported by all other eligible publications for depressive and anxiety symptoms. Standard deviations for depressive and anxiety symptoms were pooled in separated analyses. The pooled standard deviation for each outcome of interest was used in place of the missing standard deviations for Zauszneiwski and Bekhet’s (2014) publication. |
| 5 | Mean age or percentage female participants was missing in some publications (namely, Boyette et al., 2013; Fox, Barrett, & Shortt, 2002; Geller et al., 2017; Zauszniewski & Bekhet, 2014). Where this was the case, the values reported in other publications reporting on the same outcome of interest were pooled and the resulting value was used to replace missing data. E.g., Zauszniewski and Bekhet (2014) reported on anxiety symptoms but did not report the mean age of participants. The mean age reported in all other publications reporting on anxiety symptoms was pooled, and the resulting value was used to replace Zauszniewski and Bekhet’s (2014) missing data on mean age of participants in our analysis of anxiety symptoms. |
| 6 | Due to the number of studies and the limited information reported in studies, only five of our planned covariates were examined for our outcome of depressive symptoms: sibling mean age, proportion of female participants, region of study, income status of study country, and category of mental illness (MI).  Region of study was determined in accordance with the Standard Australian Classification of Countries (Australian Bureau of Statistics, 2016, May 19). Due to the limited number of included studies, all studies were classified into three regions: (1) Europe, (2) the Americas, and (3) Asia and Oceania. One study conducted in Isreal (Latzer, Katz, & Berger, 2015) and another conducted in Tunisia (Fekih-Romdhane, Nsibi, Sassi, & Cheour, 2020) could not be classified into these categories. Given the relatively small sample size in each study (30 and 60 respectively), both studies were omitted from our analysis of region of study as a covariate to siblings’ depressive symptoms.  Income status of study country was determined in accordance with The World Bank classifications (The World Bank, n.d.). Due to a lack of variation in data, studies were divided by ‘high’ and ‘medium’ income status.  Studies were divided into three categories of MI: mood disorders, eating disorders, and schizophrenia spectrum disorders (SSD). Mood disorders included all studies investigating siblings of people with an anxiety, depressive, bipolar and/or obsessive-compulsive disorder. Although schizoaffective disorder involves mood symptoms, this disorder was classified as an SSD. In light of prior literature, we anticipated that psychotic symptoms would have the most prominent impact on siblings. Where a study reported on various types of MI, the study was assigned to the category that captured the majority of participants. One study reported on siblings of people with borderline personality disorder (BPD; Laporte, Paris, Guttman, & Russell, 2011). Given that BPD did not fit into our three categories and the relatively small sample (56 participants) examined in this report, it was removed from our analysis of category of MI. |
| 7 | Only four of our planned analyses could be examined for our outcome of anxiety symptoms: sibling mean age, proportion of females, region of study, and category of MI. Studies were allocated to region of study in the same manner as outlined above for depressive symptoms. Income status of study country was not examined due to a lack of variation (i.e., only one study reporting on anxiety symptoms was conducted in a medium income status country). Only Fekih-Romdhane et al. (2020) and Laporte et al. (2011) reported on siblings of people with a disorder other than an eating or mood disorder. As such, these studies were removed from our analysis of category of MI for anxiety symptoms, and only eating and mood disorder categories were considered. |
| *Data analyses* | |
| 8 | Statistical analyses were conducted using Comprehensive Meta-Analysis (CMA) Software Version 3 (Borenstein, Hedges, Higgins, & Rothstein, 2013). To calculate the pooled mean, we used a random-effects model for each outcome of interest as per the groupings listed in step 1. |
| 9 | For each outcome of interest, we conducted sensitivity analyses to examine how the pooled mean was impacted by the inclusion of (1) unvalidated and/or subscale measures, and (2) publications deemed to be of ‘low’ quality. |
| 10 | For main analyses (step 8) with moderate or high heterogeneity and >10 studies, we conducted random-effects meta-regression to investigate potential effect modifiers (Higgins et al., 2022, August 4). Visual inspection of depressive symptom data indicated a possible difference in clinician versus self-report measures. As such, a post-hoc random-effects meta-regression was conducted to investigate instrument rater as a further possible modifier. Due to the limited number of studies, each potential modifier was examined separately in a univariate analysis. Where there were ≥10 studies per individually significant covariate, a random-effects multivariate meta-regression of significant covariates was conducted. Due to an insufficient number of studies, no covariates were examined for burden and wellbeing outcomes. |
| *Data interpretation* | |
| 11 | Where the analysis included >10 publications, we assessed small study bias visually via inspection of funnel plot asymmetry, and statistically using Egger’s regression test (Higgins et al., 2022, August 4). We used Cohrane’s Q to assess the significance of heterogeneity across studies and Higgins *I*^2^ to examine the extent of heterogeneity with the following interpretations: *I*^2^<40% = low; 40-75% = moderate; and >75% = high variance in effect size (Higgins et al., 2022, August 4). Results of all moderator analyses were assessed using Q, *I*^2^, and R^2^. For all analyses, *p* < .05 was considered statistically significant. |
| Reflective Statement | The qualitative data were predominantly synthesised by us, AJ and AW. In undertaking the synthesis of this data, we understood that we each interpreted the data through a unique lens. We are both female and largely have a western outlook. Additionally, neither of us have a sibling with mental illness. I, AJ, was born in Sri Lanka and grew up in Australia since early childhood. I am undertaking clinical training as part of my Doctorate in Psychology (Clinical). I, AW, grew up in Germany and have lived in Australia for several years. I am in the final phase of my Doctorate in Philosophy. To increase the validity of our interpretation of qualitative data, all disagreements were resolved via discussion between AJ and AW. Further input was sought from senior study authors, KF, LKB, GM, and/or SC, where required. This process aimed to bring together various interpretations of the data. |

## **Supplementary Table 7.** Results of qualitative data ratings and classifications of data according to established concepts of distress, burden, and wellbeing

| Study | (Sub)theme name | Illustrative quote | Credibility rating | Classification |
| --- | --- | --- | --- | --- |
| Amaresha et al. 2019 | Caregiving experience | He was doubting the people around him, thinking that they are plotting against him… He used to throw stones at people and pick up quarrels… Neighbours used to complain about him… It was embarrassing and uncomfortable talking to people about his condition…We were deliberately avoiding them [people]” | Unequivocal | Burden |
| Areemit et al. 2010 | Struggling to understand the ED | ‘‘Yeah it’s like, when people are saying ‘what is wrong with those people; why don’t they just control it’, you feel kind of defensive but you’re thinking the same thing.’’ | Credible | Burden |
| Areemit et al. 2010 | Increase in family conflicts and arguments | ‘‘My parents fight if she doesn’t eat and she doesn’t want to eat. So, it’s kind of weird because all you hear is yelling and stuff just because she’s not eating.’’ | Unequivocal | Burden |
| Areemit et al. 2010 | Compassion and concern for the AED | ‘‘It’s so easy for people to say ‘[you should] try not to think about it, but you can’t not think about it. How can you not think about it when someone who you love is just suffering?’’ | Credible | Distress |
| Areemit et al. 2010 | Overwhelming sense of responsibility for the AED | ‘‘I’ve always been like that; I’m responsible for everything. I always thought back to all the things that I ever said (sniffling) ‘am I like a bad sister?’ Which I know I have been a good sister but (crying), I blamed myself for that. I don’t know, I just thought ‘what did I say?’ or ‘what did I do that made her feel she wasn’t good enough?’. Maybe I should not have said, ‘those pants are too small’.’’ | Credible | Distress & Burden |
| Areemit et al. 2010 | Pervasiveness of the ED in all aspects of the siblings' life | ‘‘I know it’s not easy and it really affects everything in your life. Sure it does affect the way I see myself and it does affect the way I see the world; the way I see my sister; our relationship; how we used to talk and how we used to laugh. We don’t do that as much anymore.’’ | Unequivocal | Wellbeing |
| Areemit et al. 2010 | Acute awareness of ED behaviours and thoughts | ‘‘I hear [people saying] ‘I’m fat’ a lot more than I used to and I hear ‘I need to go on a diet’ . a lot more’’ | Unequivocal | Distress |
| Areemit et al. 2010 | Challenges in understanding noneating-related obsessive behaviours | ‘‘She would go like this and you’d see her twist her hands like that then she taps. When she is ready to go to school, she would tap the pencil on the table. When she’s brushing her teeth, she has to touch the tap. Once I tried to stop her from doing this and she said, ‘no I have to do it.’ Then I let go of it and she did it right away. Why do they do those things?’’ | Credible | Burden |
| Areemit et al. 2010 | Feelings of loss and sacrifice | Not reported | Not supported | Not applicable |
| Areemit et al. 2010 | Duality | Not reported | Not supported | Not applicable |
| Dimitropoulos et al. 2009 | Sibling role within the family: mediator and protector | I try to be there for everybody, I try to be like a counsellor, a mediator and let everybody come to me and I try to solve their problems | Unequivocal | Burden |
| Dimitropoulos et al. 2009 | Consequences and benefits of the eating disorder to the sibling | She is my big sister. She is my best friend. To see her so weak and vulnerable really scared me. Being home, by myself, while she was in the hospital having to sort out her feelings was very difficult for me. It was really difficult near the beginning when I wasn’t able to talk to her or even see her because of the program rules. I didn’t want her to think we had abandoned her. | Unequivocal | Distress & Burden |
| Dimitropoulos et al. 2009 | Strengthening of the sibling relationship | I think we have become more close because we know each other more and I guess you can say the darker sides even though it sounds dramatic. The sides that not everybody else sees. And we have been through things even our parents haven’t witnessed and I think that has just brought us closer but I wish it would happen in a more positive way instead of this continual negative way. | Unequivocal | Burden & wellbeing |
| Ewertzon et al. 2012 | Facing Existential Thoughts | “I blame myself, but I have come to understand that it did not matter how I behaved, she [the sibling] is ill anyway.” | Credible | Wellbeing |
| Ewertzon et al. 2012 | Facing Disparate Attitudes and Expectations | Pia: My parents have not been open about things. They wanted to keep the problem to themselves. I can see that it is not working. Per: It is like having gas in your stomach. You cannot keep things that way. It leads to pain. | Credible | Burden |
| Fjermestad et al. 2020 | Fear | I was scared to death. All the time. I was scared that… no, tonight, what if something happens. | Unequivocal | Distress |
| Fjermestad et al. 2020 | Frustration | Well, the entire illness is pretty annoying because it is completely meaningless to waste so much time on something so silly. I think what annoys me the most is the fact that my sister has wasted so much of her life on something completely meaningless, which she could have used to something meaningful. | Credible | Distress |
| Fjermestad et al. 2020 | Conflicts and disruptions at home | Meals were not so fun to bring friends to. It has turned out so that I haven’t brought so many friends home and I still hardly bring anyone home. It kind of turned out so that I prefer to be at others’ houses. I still almost only spend time at others. I hardly ever bring anyone home. | Credible | Burden |
| Fjermestad et al. 2020 | Limited and divided family life | Before, everyone used to sit around the table, like we did now. Then it became more divided. My sister ate and we sat there and my sister might be in her room. There was kind of an empty space there. | Unequivocal | Burden - loss |
| Fjermestad et al. 2020 | Less attention | So when aunties and such ask; How is your sister? Your sister, your sister… I just noticed that, now, what about me? I always became so frustrated. I always became like: Do I exist in this world, really, or is it just her? | Credible | Wellbeing |
| Fjermestad et al. 2020 | Changed dynamics among children in the family | Of course, she was the big sister and I was not the eldest literally, but I kind of became the eldest anyway. It was like she kind of disappeared a bit out of the sibling group. | Unequivocal | Burden - loss |
| Fjermestad et al. 2020 | Personal development | It is so incredibly important to live well with oneself, to do what makes you happy in a way (…) It is hard to explain, but you should just be satisfied. When you think about everything you have in life, it is incredibly much. I have gotten a very different view after this actually. | Credible | Wellbeing |
| Fjermestad et al. 2020 | Balanced view on food | You eat healthily and you can eat something unhealthy as well. You can treat yourself to a snack every once in a while. It is important with a balance, so that it does not become too much. So I think it has made me more aware of that, not in a negative way really. | Unequivocal | Wellbeing |
| Fjermestad et al. 2020 | Attention to other people's eating habits | It is like every time she is going to work out or eats a bit less than usual or she skips a meal, then of course I think: What if she still is sick? | Not supported | Not applicable |
| Friedrich et al. 1999 | Hallucinations and delusions | The most difficult part was at the beginning. We would be at parties together and he would start having these weird hallucinations, and you know being 18 years old, it was hard to deal with. You do not like to see your own sibling looked at like a freak. | Unequivocal | Burden |
| Friedrich et al. 1999 | Physical and verbal abuse | My brother had a violent tendency and he scared people. The hardest part was that people do not understand it is an illness. When he was violent, police would come to our house and handcuff him… then we would do the hospital and see him shackled to a bed because he was violent. | Credible | Burden |
| Friedrich et al. 1999 | Social isolation | He was almost catatonic; he would just sit there. I do not even know what thoughts were in his mind. He was zombied out. You felt like somebody was just pulling your guts out. That was just awful… when he can barely even function. That was the worst thing I ever saw. | Not supported | Not applicable |
| Gerace et al. 1993 | Beyond Normalization: Pivotal experiences | I think the hardest thing is coping with it [schizophrenia] initially. When you have a cut finger or you need stitches, you know how to seek out medical help, but people are just so thrown by mental illness that it's still kept in the dark. It's a mystery to people and there is such a stigma still attached to it that people are just totally bewildered and don't know what to do. People are prepared for disasters and catastrophes, but not how to deal with a relative who is mentally ill. | Credible | Burden |
| Gerace et al. 1993 | Continuum of illness impact | [His illness] had a dramatic impact on me from the standpoint that I stopped being a troublemaker. I stopped getting into fights. I turned from a mediocre student to really striving to do well in high school and I did. It was a dramatic change in the way I approached things. | Credible | Wellbeing |
| Hutchison et al. 2022 | Sibling role: helping | “She has gotten more and more violent with it, so then you kind of feel you have to take some of it, ‘cause like the parents can’t do it all the time … I guess as the older sibling you feel like you kind of have more a thing to protect them and help them” | Credible | Burden |
| Hutchison et al. 2022 | Sibling role: not troubling others | “[I] always have the feeling of not really wanting to put my problems on [parents], ‘cause obviously they’ve been so preoccupied with my sister … if it was just me and [them] … I’d have to hold some things back so that they didn’t feel too guilty." | Unequivocal | Burden |
| Hutchison et al. 2022 | Their needs above yours: in the family | “Yeah, I think it’s always their needs above yours … not that your parents [are], like, being intentionally, like, neglectful of you, but … I always understood that, that that’s how it needed to be.” | Credible | Wellbeing & Burden |
| Hutchison et al. 2022 | Their needs above yours: in services | “Yeah, I went, I always went to the family therapies, and it was pretty shocking because they didn’t really know what to do with me.” | Unequivocal | Burden |
| Hutchison et al. 2022 | Changes in family life | “If one person in the family has it then all … are affected by it … before I would use school to take my mind off it … but it started to get so bad that … almost, everything that someone would talk about seemed to relate to it.” | Not supported | Not applicable |
| Hutchison et al. 2022 | Changes in relationships | [I feel] “resentful … definitely. Very angry. It’s difficult to be angry with anorexia and not [sister].” | Credible | Distress |
| Jungbauer et al. 2016 | Relationship to the affected sister | I’ve always felt responsible for my sister. That’s why I never broke contact with her, even when it was difficult and burdensome. My sister is, then as now, like a daughter to me. | Credible | Burden |
| Jungbauer et al. 2016 | Burdens due to the eating disorder | I felt helpless, but also aggressive because she was so indifferent. Our parents sat there, crying, and she just sat there and said, ‘just leave me alone!’ I was so angry, and I screamed at her. One time I even resorted to violence. | Credible | Burden |
| Jungbauer et al. 2016 | Effects on the family life | I have the feeling that my parents see me on the same level as my sister, even though she’s three years older than me. I need to do things that she is doing at her age. You have to be much more independent than is normally expected at 15. | Credible | Burden |
| Jungbauer et al. 2016 | Positive Aspects, Learning Experiences and Meaningfulness | I can back off, I can basically stand on the sidelines and don’t always need to be the centre of attention. I learned through my experiences to treat people better. And I try to put myself in other people’s shoes. | Credible | Burden |
| Karlstad et al. 2021 | Taking a new role | “I performed the role of adult to a great extent. … dad was upset, my sister was upset, and I was the one comforting them” | Unequivocal | Burden |
| Karlstad et al. 2021 | Distancing | “She has been ill for so many years that you feel you have to distance yourself a little, or you are going to go crazy yourself” | Credible | Distress |
| Kovacs et al. 2019 | Communication: Controlled confrontation or cautious vagueness - 'We talk about it, but we don't exactly talk about it' | As far as I was concerned, this was a relief, that we can talk about it at home, but still, my family, we won't sit all together with Eli and start to talk about it. We'll always talk about things around [it]. At home, the television is on, and it's like very symbolic, I think, because there's never room for a real discussion, only like a little small talk… and everything stays at that level that is awfully, awfully superficial. We never talk…It's always that everyone knows what he needs to know, everyone keeps it to himself, the cards close to the chest. | Unequivocal | Burden |
| Kovacs et al. 2019 | Role shift: From sibling to parent | When she was in the closed unit, I was torn, it tore me, and in her case it was a forced hospitalization. And I had to also take her by force. This is horrible things. That is, that you find yourself having to actually be with aggression and violence in order to admit her. Then, as a result of this, I actually took a vow that I would do everything in my power that she wouldn't be hospitalized again. Once again, everything with “limited liability”, because I don't have control over everything. But everything that depends on me, I really will do everything. And I did it. And we succeeded in planning her life for her on a kind of basic level that we pushed away the chance of hospitalization. | Credible | Burden |
| Kovacs et al. 2019 | Role confusion | Ah…[sighs] to continue to be close… intense and supportive…, and however much I can, tell her the things really how I feel them, and not to play the double game. There is a lot of playacting here. …Still I think that the more we will be open and direct, it will be better and deeper. | Credible | Burden |
| Kovacs et al. 2019 | Longing for the lost sibling friendship and desire for closeness - 'He isn't my brother, in the sense of brother' | Oscar: Let's say it's more a connection of brother and sister for the protocol, I call it, despite that this sounds terrible. All along the way I have a commitment to her because she is my sister…but I don't have a friendship tie with her, like there is sometimes between siblings… It's so missing! A lot of times it's frustrating, because when you're an only child, so you say, “ O.K., I'm an only child.” But here…it's much worse, because here there is suddenly a burden… Here it's not from choice. Here you find, that essentially, he's not a brother, in the sense of a brother, we couldn't be brother and sister, essentially. | Unequivocal | Distress & Burden |
| Kristoffersen and Mustard 2000 | Ambiguous loss | It is like I am an only child. He is only physically present. He is present at Christmas. But he does not speak, and you don't have a genuine contact with one who is sick in his way. It is like you really don't have a brother. | Unequivocal | Distress & Burden |
| Kristoffersen and Mustard 2000 | The fluctuations | Yes, it is different. The hope becomes less for each time. In a way the feelings were equally deep, but there is another dimension to them. When she began to get sicker the despair also began, and this time it was genuine deep despair. Just to try to look things in the eye, that was what i believed was not true, that she will be healthy again. I see now that if I'm to avoid taking it so hard next time. i must accept the fact that it can very well go badly next time. You cannot have only despair or only hope; you must have both. | Credible | Wellbeing |
| Kristoffersen and Mustard 2000 | Prohibition | Researcher: "It is as if you have lost her, while in reality you have not lost her?" Nina: "Yes, you know when someone dies they die. In that situation you have a reason to be sorry at times, but you can obviously go back and think about the lost one. But here you have lost someone, but you have not lost them. Here you don't have permission to grieve for her in the same way. There is something there." Researcher: "There is no permission to grieve, but at the same time grief is there? There is no permission to experience the grief?" Nina: "No, she is there, therefore I have perhaps chosen to be courageous and believe that she will be better, instead of going the other way. For the most part, it is from yourself that you don't get permission to grieve. It is yourself. If you do that, it is the same as if you have pronounced the person dead. They are not dead. And so things can be extremely difficult. You ask yourself, what is it you are aiming for, the person is there, you see." | Credible | Distress & Burden |
| Kristoffersen and Mustard 2000 | Invalidation | But it seems to me that it is basically wrong, as some people do, especially in psychiatry, to comment the whole time that the relatives should not have guilt feelings. It could happen to anyone, it is not your fault. Obviously there is no one who has done something bad, but you will have guilt feelings regardless of how much people that you should not feel such. After all you live with yourself. There is nothing one can do about that. | Unequivocal | Wellbeing |
| Lukens et al. 1995 | Strategies for coping and attempts to intervene | It’s hard to determine what is the illness and what is the person, what she has control over and what she doesn’t. What should I get mad about and what should I just let slide? What’s acceptable and what’s not acceptable? How do I deal with this in a positive way and how do I encourage him slowly, not too fast? | Credible | Distress & Burden |
| Lukens et al. 1995 | Efforts to obtain services and contend with the system | Everybody—that whole system of psychiatrists, psychologists, social workers, case managers—just pushed me off, no one kept me up to speed, no one listened to me when I said “My brother’s medication is too low.” They just released him and that was it. | Credible | Burden |
| Lukens et al. 2004 | Anger and guilt | “How could I go and do something that is good for me when I know what he is going through?” | Unequivocal | Distress |
| Lukens et al. 2004 | Positive impact | “I have such a wonderful life with my family—with my brother; I would not have given that up for anything.” | Credible | Wellbeing |
| Lukens et al. 2004 | Self in relationship to partners and friends | “I might as well just get a shirt and embroider it ‘MIF’ in big red letters for ‘mentally ill family.’ I mean, what guy in his right mind is going to say, ‘Yeah, your family sounds just like the family I want to marry into.’” | Unequivocal | Burden |
| Lukens et al. 2004 | Mourning and loss (self) | “I had some fun with my brother when we were growing up and that disappeared so it was like I didn’t have a brother anymore. That was very sad and frustrating.” | Unequivocal | Distress & Burden |
| Lukens et al. 2004 | Fear | “When she was very young, 7 or 8, I was home baby-sitting and she came after me with a butcher knife. I went into the bathroom and locked the door and she tried to get the door open. I am 51 years old and I am remembering very clearly from many years ago. It was a terrifying experience. I just kept it quiet. I wouldn’t tell on her.” | Unequivocal | Distress |
| Lukens et al. 2004 | Anticipated burden | “I know I will have to take care of him and of course I will do that because he is my brother. So I have grown up being very serious when other people would be spending money going here and going there. I have just been very hard on myself.” | Unequivocal | Burden |
| Lukens et al. 2004 | Positive impact on personality | “I can really tune in with people’s pain—this is both a curse and a blessing. I’ve developed a deep compassion for people and [learned] not to make quick judgments. Wherever I work people will congregate in my office—I think they feel safe with me.” | Credible | Wellbeing |
| Lukens et al. 2004 | Mourning and loss (family) | “When I was an adolescent and my sister was first hospitalized my parents spent all their time at the hospital. I said ‘I need you too.’ They responded, ‘I know you need [us], but your sister needs [us] more.’” | Credible | Burden |
| Lukens et al. 2004 | Loss of boundaries/role confusion. | “It was hard playing the balancing act. I was ‘this’ with that person, ‘this’ with the other person, ‘this’ with my brother, a lot of fragmentation and exerted energy. The family bonds and relationships get confusing because my parents have secret conversations about my brother with me. There is a struggle because I’m not their social worker [expressing frustration about being expected to fill this role within the family]. I end up playing therapist for everything.” | Unequivocal | Burden |
| Lukens et al. 2004 | Response to family dynamic | “It was my job to be sane, to leave the house, to go to college.” “I subjugate my needs; do everything to not upset my parents.” | Credible | Burden |
| Lukens et al. 2004 | Current attitude towards family | “I’m disappointed that my parents have gotten to a point where they have just given up.” | Credible | Burden |
| Persico et al. 2021 | We Do Not Understand Why | “We often ask ourselves “why”, but there is no particular reason. It’s not like an illness where you’ve got this or that germ. . . It’s complicated. There are many different factors. . .We try to understand, but we don’t know anything, so we feel ill at ease.” | Unequivocal | Distress |
| Persico et al. 2021 | We Are Powerless | “You can’t do anything to cure them, apart from talking to them. . . we are powerless because we can’t talk to them. . . you can’t say to them “why can’t you do it? What’s the matter?” | Unequivocal | Distress |
| Persico et al. 2021 | We Feel Angry | “You can’t talk to them, you feel angry and sometimes you swear at them. . . You say that maybe it’s their fault. . . Sometimes you get angry at yourself.” | Unequivocal | Distress |
| Persico et al. 2021 | It Is Hard for the Parents | “it’s really hard when he says “no, no” and he pushes people away. . . for example: “do you want to go for a walk?”. . . “No, I don’t feel like it” and it’s always the same. . . “Have you eaten your watermelon?”. . . “No, I haven’t”. . . There it is. . . he refuses everything. . . they (the parents) are a little bit all over the place, they try to, they really do try to free the child from anorexia, to help the child. Er. . . and it’s really hard because. . . the anorexic child refuses. So, they don’t know how they can help. . . ” | Unequivocal | Burden |
| Persico et al. 2021 | We Try to Help | “We act as if it was our duty to help our sister. . . it makes us feel good inside, but at the same time, it destroys us. . .With my sister, you can never talk to her after dinner because she’s inside her own bubble. . . she blames herself and she gets angry.” | Credible | Burden |
| Riebschleger 1991 | Mixed Negative Emotions | NR | Not supported | Not applicable |
| Riebschleger 1991 | The Special Difficulties of Adult-Phase Onset | "go from the promising young man to this ghost--this shell of a person--in 18 months." | Not supported | Not applicable |
| Riebschleger 1991 | The Cyclical Illness Pattern | Siblings reported "feeling crazy" or "wondering if I'm crazy" around the time period of client deterioration, particularly just before client inpatient psychiatric hospitalization. | Not supported | Not applicable |
| Riebschleger 1991 | Mixed Messages from the Mental Health System | Siblings received conflicting messages such as "stay involved--go away," "we see you but you don't exist," "professionals know what's best," and "there are few resources professionals can provide." "it is and is not your fault." | Not supported | Not applicable |
| Samuels and Chase 1979 | Sibling relationships, guilt, and fear of madness | “It’s like having my shadow come to life. How can I talk about her, as if she’s over there and is crazy, and I’m over here and I’m healthy, when really-the things she’s acting out are the things I’m struggling with too." | Credible | Burden |
| Samuels and Chase 1979 | A new perspective on family entanglement | “I had this fantasy my omnipotent presence would change the situation. But once again the Doctor said he needed to be hospitalized, and I said, ‘He’s the Doctor.’ So I became instrumental in the whole thing, leaving him on the ward and him screaming, ‘Get me out of here!’ I had an irresponsible, lazy faith in the institutions.” | Not supported | Not applicable |
| Schmid et al. 2009 | Burden arising in the daily contact with the sibling | Not reported | Not supported | Not applicable |
| Schmid et al. 2009 | Burden with respect to the health sibling's privacy | Not reported | Not supported | Not applicable |
| Schmid et al. 2009 | Burden with respect to the contact with the family | Not reported | Not supported | Not applicable |
| Schmid et al. 2009 | Burden with respect to the contact with institutions and professionals | Not reported | Not supported | Not applicable |
| Schmid et al. 2009 | Burden with respect to the siblings' own social contacts (friends/public) | Not reported | Not supported | Not applicable |
| Schmid et al. 2009 | Positive experiences subsequent to siblings' illness | Not reported | Not supported | Not applicable |
| Scutt et al. 2022 | Feeling responsible for a sibling with anorexia nervosa | “I wouldn’t say I parented as such, but I definitely felt very responsible, felt very guilty, I felt like it was my job to help and stuff which you know I didn’t mind helping, but I do think the stress again played a big part in later mental health issues.” | Unequivocal | Burden |
| Scutt et al. 2022 | Being the well sibling | “Everyone’s like rallying around to look after this person and you feel like you kind of have to be the mature one and, like not get into much trouble and just make sure that you’re looking after things because you don’t want to put extra stress on what is already going on.” | Unequivocal | Burden |
| Scutt et al. 2022 | Difficulties getting support | “I just thought I’d be in trouble I didn’t know what was okay, and what wasn’t okay, in terms of the mental health spectrum.” | Unequivocal | Distress |
| Sin et al. 2008 | Emotional Impact | I couldn’t sleep in the night.... I would be crying day and night, I think because I used to go there [hospital] and the things she was saying…. I had so many problems, I couldn’t eat or sleep, and that just affected me badly. | Credible | Distress |
| Sin et al. 2008 | Relationships in the family | One sister talked about her concerns related her other older brother: He decides to distance himself from it. He’s the opposite to me. I [would] rather know, but he [would] rather not know; he cannot deal with it. He’s split up with his girlfriend, and one of the reasons is that he didn’t want children. While talking about her own position, she said, “I certainly don’t want a child like my brother.” | Credible | Burden |
| Sin et al. 2008 | Positive out of negatives | “It’s made me grow up quicker; it has some good effect as well because it’s made me… open up to how people feel and be more aware.” | Credible | Wellbeing |
| Sin et al. 2012 | Siblings’ roles and involvement | I get in touch with the EIPS, making sure I am there for his appointments . . . I make sure he takes his medication, gets to sleep on time and I wake him up for his appointments, even if he does not do these things I have tried my best’. | Unequivocal | Burden |
| Sin et al. 2012 | Impact on relationships | ‘It has affected . . . [the family] but in a way, like, we’ve become a really, really close family as well. Very close, so, at the same time, it’s a good experience, in getting us all close’. | Unequivocal | Wellbeing |
| Sin et al. 2012 | Diverse emotional responses | ‘I am old enough to understand, but you still want a certain amount of attention from your parents. I don’t see Mum that often, and when I see her, it’s working around my brother’ | Not supported | Not applicable |
| Stålberg et al. 2004 | Love and sorrow | "It's the sorrow that is the hardest—that someone you love so much has to suffer so much." | Unequivocal | Distress |
| Stålberg et al. 2004 | Anger and envy | "Another advantage is this thing with bills. If he doesn't bother to pay the rent no one accuses him, and he doesn't care. He doesn't feel bad about this. There are no 'have to' situations in his life." | Credible | Distress |
| Stålberg et al. 2004 | Guilt and shame | "I thought and wondered if I had anything to do with why it's like this and wondered if anyone in the family caused this illness." | Unequivocal | Burden |
| Stålberg et al. 2004 | Grieving | "It sort of hurts on the inside. Why should it happen to him? I was very sad, and there was actually a lot of crying there for a while." | Credible | Distress & Burden |
| Stålberg et al. 2004 | Fear of becoming mentally ill | "I don't know if the diagnosis had been made then, but it was tough when he was hospitalized. I felt a strong concern about getting ill myself and I was fairly paranoid about it." | Unequivocal | Distress |
| Stålberg et al. 2004 | Reflections about "bad genes" | "We kept my Mum out of it and I don't know when she found out but it was a long time later. She's ill and shouldn't have to . . . Take on that too." | Credible | Burden |

## **Supplementary Table 8.** Additional characteristics of siblings of people with mental illness in reviewed studies

| Study | Education | Marital Status | Socio-economic status | Race and/or Ethnicity | Mental health characteristics |
| --- | --- | --- | --- | --- | --- |
| *Quantitative* | | | | | |
| Alzahrani et al. 2017 | NR | NR | NR | NR | NR |
| Amaresha et al. 2018 | Mean = 12.20 years  (*SD* = 3.82) | Single: 57.50% Married: 42.50% | Upper Middle: 36.25% Middle/Lower Middle: 52.50% Lower/Upper Lower: 11.25%  Measure measured using the Kuppuswamy's revised scale. | NR | NR |
| Amianto et al. 2011 | Mean = 12.76 years  (*SD* = 4.8) | NR | Low: 6.50% Medium: 70.90% High: 22.60%  Measure not reported. | NR | No eating disorders: 100% No other relevant Axis I or II disorders: 100% |
| Avcıoğlu et al. 2019 | Primary school: 17.50% High school: 34.00% University and above: 48.50% | Single: 51.50% Married: 39.80% Divorced or widowed: 2.90% | NR | NR | NR |
| Barak and Solomon 2005 | NR | Married: > 40.00% Divorced: approximately 10.00% | NR | NR | No diagnosed mental disorder: 100% |
| Barrett et al. 2004 | NR | NR | NR | NR | NR |
| Bowman et al. 2017 | Completed year 12: 73.20% Completed a tertiary degree: 10.00% | NR | NR | NR | NR |
| Boyette et al. 2013 | NR | NR | NR | NR | NR |
| Casper 1990 | NR | NR | NR | NR | NR |
| Chen and Lukens 2011 | NR | Ever married: 75.00% Never married: 25.00% | NR | White: 35.50% Black: 19.40% Hispanic: 35.50% Other & native: 9.70% | NR |
| Christensen et al. 2007 | Mean = 12.03 years  (*SD* = 3.24) | Married or widowed: 60.94% Unmarried or divorced: 39.06% | NR | NR | Lifetime minor psychiatric diagnosis, e.g., alcohol abuse, phobia, eating disorder, etc: 28.13% |
| Deal and MacLean 1995 | Attending school: 100% | NR | Mean = 60.15 (*SD* = 12.50) Measured by the Revised Duncan Index | Caucasian: 86.67% African American: 13.30% | No history of mental illness: 100% |
| Di Sarno et al. 2021 | Mean = 12.19 years  (*SD* = 4.82) | NR | NR | NR | No psychiatric disorders: 100% |
| Diaz et al. 2021 | Mean = 14.71 years  (*SD* = 2.09) | Married: 33.3% Widowed: 4.2% Separated: 4.2% Divorced: 8.3% Never married: 25% Missing: 25% | NR | Race: Non-Hispanic White or Caucasian: 25% Hispanic or Latino: 37.5% Black or African American: 29.2% More than one race: 4.2% Unknown or not reported: 4.2%  Ethnicity: Hispanic or Latino: 41.7% Non-Hispanic or Latino: 58.3% | NR |
| Fekih-Romdhane et al. 2020 | NR | Married: 1.00% Single: 73.00% Divorced/widowed: 1.00% | NR | NR | NR |
| Fox et al. 2022 | NR | NR | NR | Asian: 2.78% Caucasian: 97.22% | Three siblings received diagnoses of subclinical oppositional defiant disorder or ADHD. |
| Geller et al. 2017 | NR | NR | NR | Caucasian: 88.90% | Past Eating Disorder: 27.80% |
| Kovacs et al. 2016 | NR | NR | NR | Caucasian: 95.00% Roma: 3.00% Multiracial/other: 2.00% | Never been depressed |
| Laporte et al. 2011 | Mean = 14.3 years  (*SD* = NR) | NR | NR | NR | BPD: 5.36% Other personality disorders: 0.00% |
| Lataster et al. 2010 | Elementary school: 1.00% Secondary school: 2.00% Higher education: 97.00% | Married or living together: 53.00% Divorced: 1.00% Never married: 46.00% | NR | NR | MDD: 23.40% No dx on Axis I: 76.60% No dx on Axis II: 100% |
| Latzer et al. 2015 | Graduated from high school: 73.00% Completed junior high school: 17.00% Completed or in the process of higher education: 10.00% | NR | NR | NR | NR |
| Mahon et al. 2013 | NR | NR | NR | White: 56.60% Other: 43.40% | Siblings were free from any major Axis I mood or psychotic disorder. Eight of the siblings were diagnosed with Depressive Disorder NOS and an additional three were diagnosed with an anxiety disorder. All participants denied substance abuse or dependence in the three months prior to their participation. |
| Matthews et al. 2021 | NR | NR | NR | Caucasian: 100% Non-hispanic: 100% | No chronic mental health conditions: 100% |
| Meneguzzo et al. 2022 | Mean = 12.77 years (*SD*= 1.84) | NR | NR | NR | NR |
| Modestin et al. 2008 | NR | Married: 44.00% Divorced/separated: 15.00% | Lower social class: 16% Measure not reported. | NR | Never been psychiatric in- or out-patients: 100% |
| Panaite et al. 2019 | NR | NR | NR | Hungarian: 100% | NR |
| Phillipou et al. 2022 | NR | NR | NR | NR | NR |
| Phoeun et al. 2022 | NR | NR | NR | NR | NR |
| Pignon et al. 2021 | NR | NR | NR | Asian: 1.10% Black: 4.90% North African: 2.30% White: 75.90% Mixed: 15.5% Other: 3.80% | No personal history of psychosis. |
| Ragazzi et al. 2020 | ≤ 9 years of education: 33.70% | Single: 46.20% | NR | NR | No history of psychosis: 100% |
| Reinhard and Horwitz 1995 | Attended some college: 57.00% | Married: 48.00% | NR | White: 62.00% Black: 29.00% Hispanic: 6.00% Asian: 3.00% | NR |
| Schick et al. 2022 | NR | NR | NR | NR | NR |
| Shivers et al. 2022 | NR | NR | NR | NR | 60.94% of participants had been diagnosed with their own mental illness. |
| Sin et al. 2016 | Completed secondary school or trade training: 41.10% Completed a tertiary degree or beyond: 58.90% | Single: 53.30% | NR | Caucasian: 66.60% Black: 12.20% Asian: 6.70% Mixed race: 14.40% | NR |
| Sletved et al. 2022 | Mean = 14.60 years  (*SD* = 2.50) | In a relationship: 63.80%  Married: 21.60%  Divorced or widowed: 7.20%  Never married: 71.20% | NR | NR | Of the 129 included sibling participants, 19 (14.7%) fulfilled ICD-10 criteria for one or several psychiatric disorders, comprising 5.4% with anxiety or OCD, 3.1% with depression, 3.1% with autism, 0.8% with acute stress disorder, 0.8% with eating disorder, 0.8% with ADHD and 0.8% with tics. Further 16 siblings (12.4%) had previously fulfilled ICD-10 criteria for a psychiatric disorder, comprising 8.5% with depression, 1.6% with anxiety, 0.8% with acute stress disorder, 0.8% with psychosis due to cannabis intake and 0.8% with substance abuse. In total 26 (20.2%) of the 129 siblings fulfilled presently or previously criteria for a psychiatric disorder. |
| Smith et al. 2016 | NR | NR | NR | Caucasian: 58.50% | NR |
| Tanaka 2008 | NR | NR | NR | American: 52.31% Japanese: 47.69% | NR |
| Tatay-Manteiga et al. 2019 | Primary: 26.10% Secondary: 43.50% University: 30.40% | Single: 39.10% Married: 52.20% Widowed: 0.00% Separated: 8.70% | NR | NR | No axis I disorders: 100% |
| Taylor et al. 2008 | Mean = 14.32 years  (*SD* = 2.32) | NR | NR | Caucasian: 100% | No serious mental illness: 100% |
| van Sprang et al. 2021 | Mean = 13.17 years  (*SD* = 3.22) | NR | NR | NR | A lifetime depressive or anxiety disorder: 50.26% |
| Zauszniewski and Bekhet 2014 | NR | NR | NR | NR | NR |
| Zhang et al. 2022 | Mean = 12.78 years  (*SD* = 4.34) | NR | NR | NR | No neurological or psychiatric disorder. |
| *Qualitative* | | | | | |
| Amaresha et al. 2019 | 11.00 years  (*SD* = 4.67) | Unmarried: 40.00% Married: 60.00% Separated: 0% | Above poverty line family: 60.00% Below poverty line family: 40.00% | NR | No mental health conditions |
| Areemit et al. 2010 | NR | NR | NR | NR | NR |
| Dimitropoulos et al. 2009 | NR | Single: 66.67% Married or in common-law relationships: 33.33% | NR | European Caucasian: 100% | NR |
| Ewertzon et al. 2012 | NR | NR | NR | NR | NR |
| Fjermestad et al. 2020 | NR | NR | NR | NR | NR |
| Friedrich et al. 1999 | High school diploma: 0% Some college: 26.67% Undergraduate degree: 40.00% Graduate degree: 33.33% | Married: 56.67% Single: 43.33% | NR | NR | NR |
| Gerace et al. 1993 | High school graduate: 7.14% Some college: 35.71% Undergraduate degree: 14.29% In graduate school: 21.43% Graduate degree: 21.43% | NR | NR | Caucasian: 92.86% African-American: 7.14% | NR |
| Hutchison et al. 2022 | NR | NR | NR | NR | None were known to have an eating disorder, but this was not formally assessed. However, this is unlikely as the recruiting service managed all eating disorder referrals in the catchment area, and all participants were seen at least once in family/multi-family therapy, without concerns being raised. |
| Jungbauer et al. 2016 | At school: 25.00% Completing a post-secondary qualification: 43.75% Working: 25.00% | Married or living with a partner: 31.25% Single: 37.50% | NR | NR | NR |
| Karlstad et al. 2021 | NR | NR | NR | NR | NR |
| Kovacs et al. 2019 | NR | Married with children: 28.57% Cohabiting with a partner: 21.43% Single: 28.57% | NR | NR | NR |
| Kristoffersen and Mustard 2000 | NR | NR | NR | NR | NR |
| Lukens et al. 1995 | College degrees: 78.95% High school graduates: 10.53% Less than a high school degree: 10.53% | NR | NR | White: 84.21% African-American: 10.53% Asian American: 5.26% | NR |
| Lukens et al. 2004 | College graduate: 78.95% High school graduate: 10.53% Less than high school degree: 10.52% | Never been married: 52.53% Currently married: 15.79% | NR | Whites: 84.21% Blacks: 10.53% Asian American: 5.26% | All participants described themselves as mentally healthy. |
| Persico et al. 2021 | NR | NR | NR | NR | NR |
| Riebschleger 1991 | NR | NR | NR | NR | NR |
| Samuels and Chase 1979 | NR | NR | NR | NR | Had been in psychotherapy as an adult: 81.82% |
| Schmid et al. 2009 | Second level primary school: 54.10% Intermediate School/Secondary/Grammar school: 40.50% Unclear/Not known: 5.40% | Single/Divorced/Widowed: 45.90% Married: 54.10% | NR | NR | NR |
| Scutt et al. 2022 | NR | NR | NR | White: 100% | Two participants had received a formal diagnosis of anorexia nervosa, eight had received no eating disorder diagnosis. Four reported previous episodes of depression, one had previously received a bipolar type 2 diagnoses and one an obsessive–compulsive disorder diagnosis. |
| Sin et al. 2008 | NR | NR | NR | White, British: 70.00% Asian, Pakistani: 10.00% Black, African: 10.00% Mixed race: 10.00% | NR |
| Sin et al. 2012 | NR | NR | NR | White British: 58.06% Black African: 6.45% Asian: 16.13% Mixed race: 19.35% | NR |
| Stålberg et al. 2004 | Mean 13 years  (Range = 10-18 years) | NR | NR | NR | Has sought psychiatric help: 43.75% Has not sought psychiatric help: 56.25% |

NR = not reported; *SD* = standard deviation; ADHD = attention deficit hyperactivity disorder; BPD = borderline personality disorder; MDD = major depressive disorder; NOS = not otherwise specified.

## **Supplementary Table 9.** Demographic characteristics of siblings with a mental illness in reviewed studies

| Study | *N* | Age  *M* (*SD*) | % Female | Education | Marital status | Socio-economic status | Race and/or Ethnicity | |
| --- | --- | --- | --- | --- | --- | --- | --- | --- |
| *Quantitative* | | | | | | | |  |
| Alzahrani et al. 2017 | NR | NR | NR | NR | NR | NR | NR | |
| Amaresha et al. 2018 | 80 | 30.86 (6.62) | 45 | Mean 10.41 years (*SD* = 4.69) | Single: 70% Married: 16.25% Separated: 7.5% Divorced: 1.25% Widowed: 5% | Upper Middle: 10.00% Middle/Lower Middle: 57.50% Lower/Upper Lower: 26.00% | NR | |
| Amianto et al. 2011 | 38 | 26.31 (7.40) | 81.58 | Mean = 12.41 years (*SD* = 3.30) | NR | Low: 7.90% Medium: 71.00% High: 21.10% | NR | |
| Avcıoğlu et al. 2019 | NR | NR | NR | NR | NR | NR | NR | |
| Barak and Solomon 2005 | NR | NR | NR | NR | NR | NR | NR | |
| Barrett et al. 2004 | 77 | 11.87 (2.74) | 51.00 | NR | NR | NR | NR | |
| Bowman et al. 2017 | 123 | 21.45 (3.51) | 29.30 | At school or university: 15.40% | NR | NR | Of Australian nationality: 61.70% | |
| Boyette et al. 2013 | 217 | 30.50 (7.60) | 17.50 | NR | NR | NR | Caucasian: 79.70% | |
| Casper 1990 | 15 | 25.70 (3.10) | 100 | NR | NR | NR | NR | |
| Chen and Lukens 2011 | 137 | 34.90 (9.22) | 40.10 | NR | Ever married: 18.20% Never married: 81.80% | NR | White: 53.00% Black: 21.60% Hispanic: 19.40% Other & native: 6.00% | |
| Christensen et al. 2007 | 128 | NR | NR | NR | NR | NR | NR | |
| Deal and MacLean 1995 | 15 | 15.20 (1.90) | NR | NR | NR | NR | NR | |
| Di Sarno et al. 2021 | 16 | 46.69 (5.49) | NR | Mean 10.13 years (*SD* = 4.50) | NR | NR | NR | |
| Diaz et al. 2021 | 56 | 36.77 (10.72) | 67.90 | Grade 6 or less: 1.80% Grade 7 to 12: 1.80% Graduated high school: 19.60% Part college: 32.10% Graduated 2 years college: 3.60% Graduated 4 years college: 28.60% Part graduate/professional school: 5.40% Completed graduate/professional school: 7.10% | Married: 7.1%  Remarried: 3.6%  Separated: 1.8%  Divorced: 5.4%  Never married: 23.2%  Missing: 58.9% | NR | non-Hispanic, White or Caucasian: 37.50% Hispanic or Latino: 21.40% Black or African American: 32.10% Asian:1.80% Hawaiian or Pacific Islander: 1.80% More than one race: 3.60% Unknown or not reported: 1.80% | |
| Fekih-Romdhane et al. 2020 | NR | NR | NR | NR | NR | NR | NR | |
| Fox et al. 2022 | 36 | 10.10 (1.77) | NR | NR | NR | NR | Asian: 2.78% Caucasian: 97.22% | |
| Geller et al. 2017 | NR | NR | NR | NR | NR | NR | NR | |
| Kovacs et al. 2016 | 218 | 17.00 (1.40) | 35.80 | NR | NR | NR | Caucasian: 95.00% Roma: 3.00% Multiracial/other: 2.00% | |
| Laporte et al. 2011 | 56 | 28.70 (NR) | 100 | Mean = 12.8 years (*SD* = NR) | NR | NR | NR | |
| Lataster et al. 2010 | 40 | 30.50 (9.40) | 40.00 | Elementary school: 6.00% Secondary school: 65.00% Higher education: 29.00% | Married or living together: 16.00% Divorced: 5.00% Never married: 79.00% | NR | NR | |
| Latzer et al. 2015 | 30 | NR | 100 | NR | NR | NR | NR | |
| Mahon et al. 2013 | 55 | 39.40 (12.20) | 65.45 | NR | NR | NR | White: 56.40% Other: 43.60% | |
| Matthews et al. 2021 | 34 | 16.10 (1.70) | 93.50 | NR | NR | NR | NR | |
| Meneguzzo et al. 2022 | 91 | 21.10 (6.49) | 100 | Mean = 12.56 years (*SD* = 2.77) | NR | NR | NR | |
| Modestin et al. 2008 | 27 | 38.00 (13.00) | 62.96 | NR | Married: 22.00% Divorced/separated: 30.00% | Lower social class: 39.00% | NR | |
| Panaite et al. 2019 | 186 | 17.11 (1.35) | 35.50 | NR | NR | Indexed by parental education: mean = 2.78 (*SD* = 1.17) | NR | |
| Phillipou et al. 2022 | 40 | 22.50 (2.91) | 100 | NR | NR | NR | NR | |
| Phoeun et al. 2022 | NR | NR | NR | NR | NR | NR | NR | |
| Pignon et al. 2021 | NR | NR | NR | NR | NR | NR | NR | |
| Ragazzi et al. 2020 | 217 | 30.70 (12.00) | 41.00 | ≤ 9 years of education: 54.8% | Single: 72.8% | NR | NR | |
| Reinhard and Horwitz 1995 | 200 | NR | 41.00 | NR | NR | NR | White: 54.00% Blacks: 37.00% Hispanics: 6.50% Asians: 2.50% | |
| Schick et al. 2022 | 96 | 33.45 (7.54) | 33.38 | NR | NR | NR | NR | |
| Shivers et al. 2022 | NR | NR | NR | NR | NR | NR | NR | |
| Sin et al. 2016 | 90 | 26.46 (7.90) | 37.80 | NR | NR | NR | NR | |
| Sletved et al. 2022 | 382 | 29.20 (NR) | 65.50 | 0–9 years: 0.5%  9–12 years: 10.50%  12–13 years: 13.60%  13–14 years: 12.30%  >14 years of education: 63.00%  Mean = 14.20 years (*SD* = 2.60) | In a relationship: 51.1%  Married: 15.20%  Divorced or widowed: 11.50%  Never married: 73.3% | NR | NR | |
| Smith et al. 2016 | NR | NR | NR | NR | NR | NR | NR | |
| Tanaka 2008 | 130 | 43.91 (13.10) | 66.92 | NR | Never married: 82.31% | NR | American: 52.31% Japanese: 47.69% | |
| Tatay-Manteiga et al. 2019 – early stage bipolar sample | 25 | 43.40 (10.30) | 52.00 | Primary: 40.00% Secondary: 36.00% University: 24.00% | Single: 44% Married: 28% Widowed: 0% Separated: 28% | NR | NR | |
| Tatay-Manteiga et al. 2019 – late stage bipolar sample | 23 | 45.10 (9.80) | 52.17 | Primary: 39.10% Secondary: 43.50% University: 17.40% | Single: 34.8% Married: 52.2% Widowed: 0% Separated: 13% | NR | NR | |
| Taylor et al. 2008 | NR | 62.18 (4.95) | 66.30 | NR | Currently married: 56.60% | NR | Caucasian: 100% | |
| van Sprang et al. 2021 | 256 | 48.52 (13.10) | 73.40 | Mean 13.42 years (*SD* = 2.99) | NR | NR | NR | |
| Zauszniewski and Bekhet 2014 | 60 | 37.75 (13.96) | NR | NR | NR | NR | NR | |
| Zhang et al. 2022 | 29 | 34.86 (12.02) | 79.31 | Mena 14.21 years (*SD* = 3.79) | NR | NR | NR | |
| *Qualitative* |  |  |  |  |  |  |  | |
| Amaresha et al. 2019 | 15 | 30.40 (5.42) | 20.00 | Mean 10.26 years (*SD* = 4.92) | Unmarried: 86.70% Married: 6.70% Separated:6.70% | Above the poverty line family: 53.33% Below the poverty line family: 46.66% | NR | |
| Areemit et al. 2010 | NR | NR | NR | NR | NR | NR | NR | |
| Dimitropoulos et al. 2009 | 12 | 25.40 (7.34) | 100 | NR | Single: 91.67% Common-law relationship: 8.33% | NR | European Caucasian: 100% | |
| Ewertzon et al. 2012 | NR | NR | NR | NR | NR | NR | NR | |
| Fjermestad et al. 2020 | NR | NR | 90.00 | NR | NR | NR | NR | |
| Friedrich et al. 1999 | 22 | 37.00 (NR) | 31.82 | High school diploma: 31.82% Some college: 50.00% Undergraduate degree: 18.18% Graduate degree: 0% | Married: 4.55% Single: 95.45% | NR | NR | |
| Gerace et al. 1993 | 14 | 33.00 (NR) | 50.00 | NR | NR | NR | NR | |
| Hutchison et al. 2022 | 14 | 15.4 (NR) | 100 | NR | NR | NR | NR | |
| Jungbauer et al. 2016 | NR | 21.90 (6.80) | 100 | NR | NR | NR | NR | |
| Karlstad et al. 2021 | 8 | NR | 100 | NR | NR | NR | NR | |
| Kovacs et al. 2019 | NR | NR | NR | NR | NR | NR | NR | |
| Kristoffersen and Mustard 2000 | NR | NR | NR | NR | NR | NR | NR | |
| Lukens et al. 1995 | 19 | NR | 26.32 | NR | NR | NR | NR | |
| Lukens et al. 2004 | 19 | NR | 26.32 | NR | NR | NR | NR | |
| Persico et al. 2021 | NR | NR | NR | NR | NR | NR | NR | |
| Riebschleger 1991 | 14 | 29.00 (NR) | 21.43 | NR | NR | NR | NR | |
| Samuels and Chase 1979 | 11 | NR | 81.82 | NR | NR | NR | NR | |
| Schmid et al. 2009 | 37 | 39.90 (NR) | 37.80 | NR | Single/Divorced/Widowed: 94.6% Married: 5.4% | NR | NR | |
| Scutt et al. 2022 | NR | NR | 100 | NR | NR | NR | NR | |
| Sin et al. 2008 | 9 | 24.20 (NR) | 11.11 | NR | NR | NR | White, British: 66.67% Asian, Pakistani: 11.11% Black, African: 11.11% Mixed race: 11.11% | |
| Sin et al. 2012 | 25 | 23.50 (4.45) | 20.00 | NR | NR | NR | White British: 60.00% Black African: 8.00% Asian: 16.00% Mixed race: 16.00% | |
| Stålberg et al. 2004 | 14 | 32.00 (NR) | 35.71 | Mean 12 years (*SD* = NR) | NR | NR | NR | |

*N* = sample size; *M* = mean; *SD* = standard deviation; NR = not reported.

## **Supplementary Table 10.** Illness-related characteristics for siblings with a mental illness and eligibility criteria for reviewed studies

| Study | Diagnoses | Diagnostic framework | Method of diagnostic assessment | Duration of illness *M* (*SD*) | Additional details of illness | Comorbidities | Setting | In/exclusion criteria |
| --- | --- | --- | --- | --- | --- | --- | --- | --- |
| *Quantitative* |  |  |  |  |  |  |  |  |
| Alzahrani et al. 2017 | SMI: 100% | NR | Clinician appraisal | NR | NR | NR | NR | NR |
| Amaresha et al. 2018 | Schizophrenia:  100% | DSM-IV | Structured interview: MINI | 5.06 (4.76) | Age at onset: *n*=79, *M*=25.68 (*SD*=5.96) Duration of untreated psychosis: *n*=79, *M*=2.50 (*SD*=3.18) Total psychopathology: *n*=80, *M*=53.70 (*SD*=32.56) Age at treatment: *n*=79, *M*=28.19 (*SD*=6.24) Total years of treatment: *n*=79, *M*=2.54 (*SD*=3.28) | No axis 1 psychiatric co-morbidity | NR | - no axis 1 psychiatric co-morbidity - no clinical emergency (e.g., suicidality, aggression, catatonia, etc) |
| Amianto et al. 2011 | Anorexia nervosa: 100% | DSM-IV | Structured interview: SCID | NR | NR | NR | NR | - Admitted to the Regional Referral Pilot Centre for Eating Disorders of Torino University between December 2005 and August 2008  - presence of a full (and stable at least for 1 year) diagnosis of anorexia nervosa (either restricting or binge-purging type)  - at least 16 years old  - had at least one sibling aged 14 years or older living with them in the same family. |
| Avcıoğlu et al. 2019 | Schizophrenia: 100% | NR | NR | Mean = 14.59 (*SD* = 9.05) | NR | NR | NR | NR |
| Barak and Solomon 2005 | Schizophrenia: 100% | NR | NR | NR | NR | NR | NR | NR |
| Barrett et al. 2004 | Obsessive Compulsive Disorder: 100% | DSM-IV | NR | NR | Most of the sample presented with three or more compulsions, and two or more obsessions. The most common compulsions included washing/cleaning rituals (57% of sample) and checking for reassurance (57% of entire sample). The most common obsessions included fears of contamination/illness or disease (75% of sample) and aggressive obsessions, including fears of harm to self and others (62% of sample). | 79% of the sample presented with a secondary comorbid diagnosis, with 59% of the sample presenting with more than one comorbid diagnosis. The most common comorbid diagnosis was GAD (n= 46), followed by specific phobia (n= 27), then social phobia (n= 15), separation anxiety (n= 13), dysthymia (n= 4), and MDD (n= 2). | Out-patients: 100% | - aged 7-17  - meets criteria for OCD  - at least one parent willing to attend weekly sessions  - no primary major depression or another primary anxiety disorder, primary externalizing disorder (including ADHD, ODD, or conduct disorder), Tourette’s syndrome, ASD, schizophrenia, organic mental disorder, or mental retardation  - not receiving concurrent psychotherapy  - not receiving concurrent pharmacotherapy for less than 3 months  - IQ suspected to be within normal range. |
| Bowman et al. 2017 | Schizophrenia: 41.50% Schizophreniform: 38.20% Schizoaffective: 4.90% Bipolar affective disorder: 10.60% Post traumatic stress disorder: 4.10% Post partum psychosis: 0.80% Persisting psychosis: 52.00% | NR | Clinician appraisal | NR | Has required a hospital admission due to SMI: 78.30% Length of duration of untreated psychosis as stated by sibling: 1–6 months: 26.70% 7–12 months: 38.80% ≥13 months: 34.30% | NR | NR | Client at EPPIC with FEP. It was an ethical requirement to obtain consent from the young person experiencing FEP for their sibling to participate in the study. |
| Boyette et al. 2013 | Schizophrenia: 73.70% Schizophreniform: 1.80% Schizoaffective: 12.40% Delusional Disorder: 0.90% Psychotic Disorder NOS: 11.10% | DSM-IV | NR | NR | Age of onset for FEP: M = 22.80 years (*SD* = 7.10) No. of episodes in past 3 years: 0: 73.30% 1: 17.50% 2-5: 4.10% Unknown: 5.10% | NR | NR | - aged 18 to 50 - meets DSM-IV criteria for a non-affective psychotic disorder; schizophrenia, schizophreniform disorder, schizoaffective disorder, delusional disorder, or psychotic disorder NOS - maximum duration of illness of 10 years - fluent in Dutch - participating in NEO-FFI assessment |
| Casper 1990 | Anorexia Nervosa: 100% | Feighner et al.'s (1972) diagnostic criteria | Clinician appraisal | NR | Fully recovered: *n* = 18 Good outcome with weight preoccupation: *n* = 6 Mean recovery period was 5.9 years (+/- 2.4 years) | NR | NR | - evaluated and treated for AN between 1975 and 1978 at the Illinois State Psychiatric Institute  - qualified for 'good outcome' in accordance with Morgan & Russell criteria. |
| Chen and Lukens 2011 | Schizophrenia: 78.10% Schizoaffective disorder: 3.70% Other affective disorder: 18.20% | DSM-III | NR | Mean = 13.47 years (*SD* = 8.39) | NR | NR | In-patients: 100% | - DSM-III-R diagnosis of schizophrenia or affective disorder  - aged 18 to 65 |
| Christensen et al. 2007 | Depression, Recurrent depression, a first diagnosis of manic mixed episode, or bipolar affective disorder. | ICD-8 or ICD-10 | Clinician appraisal | NR | NR | NR | NR | Probands were identified as twins who on their first admission, in the period between 1968 and 2005, were discharged from a psychiatric hospital with a diagnosis of depression or recurrent depression; a first diagnosis of manic mixed episode; or bipolar affective disorder. |
| Deal and MacLean 1995 | Major Depression: 26.67% Major Depression/Poly Substance Use Disorder: 20.00% Intermittent Explosive Disorder: 13.33% Oppositional Defiant Disorder: 13.33% Major Depression/PTSD: 6.67% Dysthymia: 6.67% Conduct Disorder: 6.67% Adjustment Disorder with mixed disturbance of mood and conduct: 6.67% | DSM-III | NR | NR | NR | NR | NR | - had been psychiatrically hospitalised |
| Di Sarno et al. 2021 | Schizophrenia: 100% | DSM-5 | Clinician appraisal | Mean = 22.94 years (*SD* = 9.66) | Number of hospitalisations: *M* = 4.56 (*SD* = 4.53) Age at disease onset: *M* = 23.06 (*SD* = 7.15) | NR | Outpatients: 100% | - outpatients diagnosed with schizophrenia by a psychiatrist according to DSM-5 - aged from 18 to 60 years - currently receiving medication for schizophrenia - stable condition for at least 6 months |
| Diaz et al. 2021 | BD-I: 61.20% BD-II: 8.80% | DSM-IV | Structured interview: SCID | NR | Age at first diagnosed with BD: *M* = 27.73 (*SD* = 9.93) | NR | NR | Exclusion criteria included a history of diabetes, hypertension, liver insufficiency, kidney insufficiency, cardiovascular problems, systemic infections, cancer, hypothyroidism, auto-immune diseases, and brain disorders as seizure disorder, stroke, dementia, and neurodegenerative diseases. The local Institutional Review Board approved the study protocol. Informed consent was obtained from all the participants. |
| Fekih-Romdhane et al. 2020 | Schizoprhenia: 100% | DSM-V | NR | NR | NR | NR | NR | NR |
| Fox et al. 2022 | GAD: 55.60% Separation Anxiety: 25.00% Social Phobia: 13.90% Specific Phobia: 5.60% | DSM-IV | Structured interview: DISCAP | NR | NR | 63.9% had comorbid diagnoses: GAD 19.4%, social phobia 16.7%, specific phobia 16.7%, separation anxiety disorder 11.1%, panic disorder 2.8%. Seven SMI children received diagnoses of subclinical externalising disorder (ODD or ADHD). | NR | To be included in the anxious group, SMI child needed to meet diagnostic criteria for a current DSM-IV anxiety disorder with a clinical level of severity. |
| Geller et al. 2017 | Eating disorder: 100% | NR | NR | NR | NR | NR | NR | NR |
| Kovacs et al. 2016 | Depression: 100% Remitted: 85.32% Currently depressed: 14.68% | DSM-IV | Structured interview: DISCAP | NR | Remitted: *n* = 186 Depressed: *n* = 32 Age of onset for total proband sample: *M* = 9.07 years (*SD*: 1.90) Fifty-six percent had 1 MDE, 31.2% had 2 MDEs, and 12.9% had 3 or more MDEs. Thirty-two probands, 14.7%, were in a depressive episode while 186, 85.3%, had recovered from their last MDE. | Anxiety related disorder: 39.00% Behavioural Disorder (e.g., ADHD, ODD): 36.24% 1 or more comorbid major psychiatric disorder: 70.20% | NR | - had a current or recent DSM-IV diagnosis of depressive disorder  - were 7 to 14 years old at initial screen  - were not mentally retarded and had no major systemic medical disorder  - had at least one biological parent who could participate  - had at least one full biological 7-to-18-year-old sibling (within ±3 years of age) |
| Laporte et al. 2011 | Borderline Personality Disorder: 100% | DSM-IV & DIB-R | Structured interview: SCID | NR | NR | NR | NR | - participants were required to meet DSM-IV-TR criteria for BPD and to score 8/10 on the Diagnostic Interview for BPD, Revised  - the only exclusion criteria for the sample were organic brain syndromes and mental retardation. |
| Lataster et al. 2010 | Schizophrenia: 67.50% Schizoaffective Disorder: 15.00% Psychotic Disorder NOS: 7.50% Brief Psychotic Disorder: 7.50% Delusional Disorder: 2.50% | DSM-IV | Structured interview: CASH | NR | CASH delusions lifetime: *M* = 4.20 (*SD* = 2.20), range: 1–8 CASH hallucinations lifetime: *M* = 2.1 (*SD* = 1.6), range: 0–5 CASH positive symptoms: *M* = 6.4 (*SD* = 2.9), range: 1–11 Age at first psychotic episode (years): *M* = 23.4 (*SD* = 7.3), range: 13–44 Number of episodes: *M* = 2.4, range: 1–6 | NR | NR | - lifetime occurrence of psychotic symptoms in the context of a non-affective psychotic disorder, according to DSM-IV criteria  - age 16–60 years  - sufficient command of the Dutch language. |
| Latzer et al. 2015 | Anorexia nervosa: 33.33% Bulimia nervosa: 33.33% ED NOS: 33.33% | DSM-IV | Clinician appraisal | NR | All sisters with EDs were in the active stage of the illness. | NR | NR | NR |
| Mahon et al. 2013 | BD-I: 85.45% BD-II: 9.09% BD NOS: 5.46% | DSM-IV | Structured interview: SCID | NR | None of the patients were related to one another and all were clinically stable at the time of the assessment. | NR | Out-patients: 100% | NR |
| Matthews et al. 2021 | Anorexia nervosa: 100% | NR | Clinician appraisal | Mean = 1.00 years (*SD* = 1.10) | Has received at least one medical hospitalisation for eating disorder related complications: 61.8% Has a history of inpatient psychiatric hospitalisation for AN: 50% Has received residential treatment for AN: 44.1% | NR | NR | NR |
| Meneguzzo et al. 2022 | Anorexia nervosa: 52.75%  Bulimia nervosa: 26.37%  Binge-eating disorder: 8.79%  Other specified eating disorders: 12.09% | DSM-5 | Clinician appraisal | NR | NR | NR | Out-patients: 100% | The inclusion criteria for all participants were as follows: they identify as women; be between 14 and 40 years old, which is the usual range of ages of patients treated at the ED unit; and have no history of psychotic symptoms or severe medical conditions. Patients with EDs fulfilled the DSM-5 criteria for EDs as evaluated in person by a trained psychiatrist. |
| Modestin et al. 2008 | Schizophrenia Spectrum Disorder: 37.00% Affective Disorder: 33.00% Mostly Neurotic Disorders: 30.00% | ICD-10 | Clinician appraisal | NR | NR | NR | NR | NR |
| Panaite et al. 2019 | childhood onset Depressive Disorder: 100% | DSM-IV | NR | NR | Depressive symptoms on the CDI-2: *M* = 9.28 (*SD* = 6.34) Anxiety symptoms on the MASC: *M* = 31.79 (*SD* = 13.41) Baseline Positive Affect levels: *M* = 3.27 (*SD* = 1.56) | NR | NR | - history of a depression disorder, based on DSM–IV criteria  - aged 7–14 years; absence of mental retardation or major medical disorder  - has one full biological sibling aged 7–18 years  - has one biological parent available to participate. |
| Phillipou et al. 2022 | Anorexia Nervosa: 100% | DSM-5 | Structured interview: MINI | Mean = 5.04 years (*SD* = 3.52) | Age of onset:  Mean 14.96 years (2.30) | NR | NR | All participants were required to be female, English speaking and over 18 years of age. Probands were required to have a previous diagnosis of AN by a health professional (psychologist or psychiatrist) and to have maintained a body mass index of 18.5 or over for the previous 12 months. |
| Phoeun et al. 2022 | NR | NR | NR | NR | NR | NR | NR | NR |
| Pignon et al. 2021 | FEP: 100% | ICD-10 | Clinical appraisal | NR | NR | NR | NR | - presence of at least one positive psychotic symptom for at least 1 day duration or two negative psychotic symptoms (for at least 6 months duration) within the timeframe of the study  - aged between 18 and 64 years (inclusive)  - resident within a clearly defined catchment area at the time of their first presentation. Residence was defined as a minimum of a one night stay at a residential address within the catchment areas  - no previous contact with specialist mental health services for psychotic symptoms outside of the study period at each site  - no evidence of psychotic symptoms precipitated by an organic cause (ICD-10: F09  - no transient psychotic symptoms resulting from acute intoxication (F1X.5)  - no severe learning disabilities, defined by an IQ less than 50 or diagnosis of intellectual disability (F70–F79) |
| Ragazzi et al. 2020 | Schizophrenia spectrum disorder: 100% | DSM-IV | Structured interview: SCID | NR | NR | NR | NR | - residing in the Ribeirao Preto epidemiologic catchment area - made first contact with a mental health service due to the manifestation of psychotic symptoms during the 3-year period of the STREAM study - probands whose psychotic symptoms were due psychoactive substance use or another medical condition were excluded |
| Reinhard and Horwitz 1995 | Schizophrenia: 51.00% Major Depression: 22.00% Bipolar Disorder: 9.00% | NR | NR | NR | NR | NR | In- and out-patients | NR |
| Schick et al. 2022 | Non-affective psychosis: 100% | DSM-IV | NR | NR | NR | NR | NR | - age between 16 and 50 years  - meeting full DSM-IV criteria for a non-affective psychotic disorder  - estimated level of intelligence above 70 |
| Shivers et al. 2022 | The diagnoses included a broad range of disorders such as: depression, bipolar, and eating disorders | NR | NR | NR | NR | NR | NR | The criteria for inclusion in the study required that both siblings were at least 18 years of age and at least one had been diagnosed with MI. |
| Sin et al. 2016 | First Episode Psychosis: 100% | NR | NR | NR | NR | NR | NR | NR |
| Sletved et al. 2022 | Bipolar disorder (type I): 31.90%  Bipolar disorder (type II): 68.10% | ICD-10 & DSM | Structured interview: SCAN | 10.50 years (*SD* = NR) | Time with diagnosis (yrs): 0.3 (SD = NR)  Untreated bipolar years (yrs): 4.8 (SD = NR)  Number of affective episodes: 12 (SD = NR)  Percentage of patients with one or more previous admissions to psychiatric hospital: 44.10% | NR | NR | All patients referred to the clinic with newly diagnosed BD or a diagnosis of a single manic episode were invited to participate in the BIO study. Inclusion criteria for participation in the BIO study were an ICD-10 diagnosis of BD set within the preceding two years, chosen as our definition of a newly diagnosis with BD. |
| Smith et al. 2016 | Schizophrenia: 100% | DSM-IV | Structured interview: SCID | NR | Age of onset: Mean 16.8 years (*SD* = 4.5) | NR | NR | NR |
| Tanaka 2008 | Schizophrenia spectrum disorders: 94.62% | DSM-IV & ICD-10 | Sibling report | NR | Regularly taking medication at the time of study: 58.60% | NR | Inpatients: 10% | NR |
| Tatay-Manteiga et al. 2019 | Bipolar disorder: 100% | DSM-IV | Structured interview: SCID | Mean = 20.10 months (*SD* = 10.9) | Age of onset (years): *M* = 23.00 (*SD* = 7.00) No of episodes: *M* = 24.80 (*SD* = 49.90) | NR | Out-patient or living in residence. | - aged 18–60 years old  - diagnosed with DSM-IV-TR BD type I  - outpatient or living in a residence  - clinically euthymic confirmed with psychometric criteria (HRSD < 8 and YMRS < 7) for at least two months  - receiving a stable regimen of medication for at least 4 weeks  - able to understand the study procedures and to provide written informed consent  - not currently hospitalised  - no cognitive impairment (intellectual disability or dementia); physical, visual or hearing disabilities that would prevent from understanding the protocol  - ability to read or understand Spanish |
|  | Bipolar disorder: 100% | DSM-IV | Structured interview: SCID | Mean = 18.00 years (SD = 9.70) | Age of onset (years): M = 26.90 (SD = 7.90) No of episodes: M = 11.00 (SD = 8.90) | NR | Out-patient or living in residence. | Inclusion: 18–60 years old; diagnosed with DSM-IV-TR BD type I; outpatient or living in a residence; clinically euthymic confirmed with psychometric criteria (Hamilton Rating Scale for Depression, HRSD < 8 and Young Mania Rating Scale, YMRS < 7) for at least two months; receiving a stable regimen of medication for at least 4 weeks; and able to understand the study procedures and to provide written informed consent.  Exclusion: current hospitalization; cognitive impairment (intellectual disability or dementia); physical, visual or hearing disabilities that would prevent from understanding the protocol; and inability to read or understand Spanish. |
| Taylor et al. 2008 | Depressive disorder, anxiety disorder, or both: 85.00% Episodic mood disorder: 8.40% Schizophrenic disorder: 3.60% Alcohol dependence syndrome: 2.40% | NR | Self-report | NR | NR | NR | NR | We excluded from this analysis any respondents with mild ID who also reported an MI or a major depressive episode. The sibling of interest must have completed both the telephone interview and mail-back survey in 2004 –2005. This resulted in 791 siblings in the comparison group. In all groups, cases were excluded if either member of the selected sibling pair had died prior to the 2004 –2005 interview. |
| van Sprang et al. 2021 | Depressive and/or Anxiety Disorder: 100% | DSM-IV | Structured interview: CIDI - lifetime version 2.1 | NR | NR | NR | NR | - a depressive and/or anxiety disorder diagnosis assessed with the CIDI on at least two NESDA waves - 100% the same biological parents as their siblings - participated in at least three out of four NESDA face-to-face interviews prior to the 9-year follow-up (i.e., from baseline to 6-year follow-up) - availability of genetic data - provided approval of contacting siblings for research purposes - participated at the 9-year follow-up face-to-face interview |
| Zauszniewski and Bekhet 2014 | Schizophrenia: 45.00% Bipolar disorder: 45.00% Major Depressive Disorder: 8.00% Panic disorder: 2.00% | NR | Sibling report | Mean = 11.21 years (*SD* = 9.65) | NR | NR | NR | SMI defined as SCZ, BD, MDD or an anxiety disorder. |
| Zhang et al. 2022 | Major Depressive Disorder: 100% | DSM-IV | Structured interview: SCID | Mean = 7.70 (*SD* = 5.70) | Number of depressive episodes: *M* = 2.24 (*SD* = 1.12) | NR | NR | Inclusion criteria for remitted MDD patients were: (1) aged between 18 and 60 years; (2) had no other psychiatric disorders apart from MDD and had no history of neurological illness; (3) able to undergo an MRI scan. |
| *Qualitative* |  |  |  |  |  |  |  |  |
| Amaresha et al. 2019 | Schizophrenia: 100% | DSM-IV | NR | NR | NR | NR | NR | - a biological sibling of a person diagnosed with schizophrenia (DSM-IV) - aged between 18 and 60 years - able to speak English, Kannada, or Telugu - no mental illness according to M.I.N.I 5.0 International Neuropsychiatric Interview - found not to be suffering from any medical illness according to a qualified medical professional |
| Areemit et al. 2010 | Anorexia Nervosa: 60% EDNOS: 40% | NR | NR | Mean 1.97 years (*SD* = 1.36) | NR | NR | NR | has ED diagnosed for >3 months |
| Dimitropoulos et al. 2009 | Anorexia Nervosa: 100% | NR | Clinician appraisal based on caregiver report. | Mean 8.2 years (*SD* = 4.63) | Age at which symptoms were first noticed:  *M* = 17.6 years (*SD* = 4.40) | NR | NR | - the family member and their relative with AN were 16 years of age or older at the time of the study - the ill family member’s diagnosis of AN was established by a health professional according to caregiver report - the ill person’s estimated BMI was 18.5 or less - the caregiver knew of the illness for at least 1 year - the caregiver was biologically or legally (e.g., common-law or marriage) related to the family member with AN.  - no significant developmental delay in the person with AN - no psychotic disorder in the person with AN |
| Ewertzon et al. 2012 | Psychotic illness: 100% | NR | NR | Mean 17 Years (*SD*  = NR) | Range of illness duration: 1-45 years Nine had been ill for >10 years | NR | NR | NR |
| Fjermestad et al. 2020 | Anorexia Nervosa: 100% | NR | NR | NR | Patients had lived with anorexia nervosa for a mean of 3 years at the time of admission. At the time of the survey, three patients still had an eating disorder. | NR | NR | Patients had been assigned and used a bed at the inpatient eating disorder unit. |
| Friedrich et al. 1999 | Schizophrenia: 100% | NR | NR | Mean 18 years (*SD* = NR) | The majority had been hospitalised at least six times and 25% had spent more than 3 years in a hospital. | NR | NR | NR |
| Gerace et al. 1993 | Schizophrenia: 100% | NR | NR | NR | NR | NR | NR | - has a history of at least three psychiatric hospitalizations for positive symptoms of schizophrenia (hallucinations, delusions, incoherence or loose association, inappropriate affect) - evidence of chronic impairment (decreased level of functioning, need for supervised living arrangements) - receives ongoing psychiatric care (periodic assessments, continuing neuroleptic pharmacotherapy) |
| Hutchison et al. 2022 | Anorexia nervosa or a similar restrictive eating disorder. | NR | NR | 2.90 years (*SD* = NR) | NR | NR | NR | NR |
| Jungbauer et al. 2016 | Anorexia Nervosa: 100% | ICD-10 | NR | Mean 6.2 years (*SD* = 5.3) | Age of onset: 15.8 years (*SD* = 4.5) Completed at least one inpatient treatment: 62.50% | NR | NR | - has an ICD-10 diagnosis for ‘‘anorexia nervosa’’ (F 50.0) - has had diagnosis for a least 1 year - siblings have contact with their each other, or at least have lived together as a children or teenagers. |
| Karlstad et al. 2021 | Anorexia Nervosa: 75% Bulimia Nervosa: 25% | NR | NR | Long duration (± 10 years) of illness was common for the majority of the group, while a minority had been ill for a shorter term. | For the majority of the siblings affected by ED, onset of the illness took place in their early teens. A few were in their early twenties. All had at some point received treatment at inpatient or outpatient clinics and some were still being treated. None of the siblings were referred to as fully recovered from the ED. Most fluctuated between better and worse periods. | NR | NR | Participants were the brothers and sisters of adult women (over the age of 18 years) with AN or BN. |
| Kovacs et al. 2019 | The diagnoses are varied—schizophrenia, bi‐polar disorder, O.C.D., depression, anxiety and eating disorder. | NR | Sibling report | The duration of the sibling's illness is between four and 30 years. | NR | NR | NR | - being a sibling of a person who underwent psychiatric treatment for at least 3 years for a severe mental health problem (as defined by participants). |
| Kristoffersen and Mustard 2000 | Schizophrenia: 100% | NR | Clinician appraisal | Mean 13 years (*SD* = NR) | NR | NR | Inpatients: 100% | NR |
| Lukens et al. 1995 | Schizophrenia or schizoaffective disorder: 68.42% Bipolar disorder: 21.05% Major depression: 10.53% | NR | NR | Range: 5 to >40 years | NR | NR | NR | NR |
| Lukens et al. 2004 | Schizophrenia or schizoaffective disorder: 68.42% Bipolar disorder: 21.05% Major depression: 10.53% | NR | Sibling report | Most (*n* = 17) reported that the sibling had been ill since childhood or adolescence so that the well sibling had had to cope with the illness during his or her own childhood. | NR | NR | NR | NR |
| Persico et al. 2021 | Anorexia Nervosa: 100% | NR | NR | NR | NR | NR | NR | NR |
| Riebschleger 1991 | Schizophrenia: 71.43% Bipolar affective disorders: 21.43% Organic impairment resulting in mental illness: 7.14% | NR | NR | NR | Every client had a history of multiple inpatient psychiatric hospitalizations and adult-phase onset of the psychiatric disorder. | NR | Outpatients: 100% | NR |
| Samuels and Chase 1979 | Schizophrenia: 100% | NR | Sibling report | NR | NR | NR | NR |  |
| Schmid et al. 2009 | Schizophrenia: 100% | ICD-10 | Clinician appraisal | Up to, and including, 5 years: 27% 6-10 years: 13.5% More than 10 years: 59.5% | Current degree of severity of illness (Clinical Global Impression) Slightly to moderately ill: 29.70% Notably ill: 43.20% Seriously ill: 27.00% Extremely ill: 0%  Number of inpatient treatments so far, including current admission 1st stay: 5.40% 2-5 stays: 40.50% 6-10 stays: 21.60% 11-15 stays: 18.90% More than 15 stays: 8.10% Unclear/Not known: 5.40%  Outpatient support for the patient provided by (multiple choices possible) Psychiatrist: 78.40% Other (general practitioner, psychotherapist, ergotherapist): 29.70% | NR | Inpatient: 100% | Inpatients with a schizophrenic disorder (according to ICD-10) were asked for their permission to invite their sibling who was emotionally closest to them to an interview. Adoptive siblings and siblings who had a psychiatric disorder themselves (e.g., oligophrenia, dementia) were not eligible for inclusion. |
| Scutt et al. 2022 | Anorexia nervosa: 100% | NR | Sibling report | NR | NR | NR | NR | NR |
| Sin et al. 2008 | First-episode psychosis: 100% | NR | NR | Mean 21 months; range 1-3 years | All were "receiving a service from the local EIPS" but no other details reported. | NR | Outpatients: 100% | NR |
| Sin et al. 2012 | First-episode psychosis: 100% | NR | NR | Mean = 18 months; range: 6 months to 4 years | NR | NR | NR | NR |
| Stålberg et al. 2004 | Schizophrenia spectrum disorder: 100% | DSM-IV | NR | NR | Estimated age of onset of schizophrenia:  Mean 22 years  Range: 15-31 years Currently on psychopharmacologic medication: *n* = 12 | NR | NR | Siblings of patients with a schizophrenia or schizophrenia spectrum diagnosis were eligible. Siblings with psychiatric symptoms or earlier psychiatric contact were not excluded. |

*M* = mean; *SD* = standard deviation; NR = not reported; SMI = serious mental illness; DSM = Diagnostic and Statistical Manual of Mental Disorders; MINI = Mini-International Neuropsychiatric Interview; SCID = Structured Clinical Interview for DSM; GAD = generalised anxiety disorder; OCD = obsessive-compulsive disorder; ADHD = attention deficit hyperactivity disorder; ASD = autism spectrum disorder; MDD = major depressive disorder; IQ = intelligence quotient; FEP = first episode psychosis; NOS = not otherwise specified; NEO-FFI = NEO Five-Factor Inventory; AN = anorexia nervosa; ICD = International Classification of Diseases; BD = Bipolar Disorder; DISCAP = Diagnostic Interview Schedule for Children Adolescents and Parents; MDE = major depressive episode; ODD = oppositional defiance disorder; DIB-R = Diagnostic Interview for Borderline Personality Disorder – Revised; BPD = Borderline Personality Disorder; CASH = Comprehensive Assessment of Symptoms and History; ED = eating disorder; CIDI = Composite International Diagnostic Interview; NESDA = Netherlands Study of Depression and Anxiety; SCZ = schizophrenia; BN = bulimia nervosa.

## **Supplementary Table 11.** Characteristics of the sibling relationship in reviewed studies

| Study | Living arrangements | Type of relationship | Biological status |
| --- | --- | --- | --- |
| *Quantitative* | | | |
| Alzahrani et al. 2017 | Not reported | Not reported | Not reported |
| Amaresha et al. 2018 | Together: 66.25% Apart: 33.75% | Sibling involvement in care: M = 20.96 (*SD* = 3.99) according to a 16-item schedule prepared for the purpose of the study. Scores ranged from 0-32 with higher scores indicating better involvement. | Biological: 100% |
| Amianto et al. 2011 | Together: 100% | Not reported | Not reported |
| Avcıoğlu et al. 2019 | Together: 48.50% Apart: 51.50% | Not reported | Not reported |
| Barak and Solomon 2005 | Not reported | Not reported | Not reported |
| Barrett et al. 2004 | Not reported | Not reported | Not reported |
| Bowman et al. 2017 | Together: 63.10% | Not reported | Biological: 98.10% |
| Boyette et al. 2013 | Not reported | Not reported | Not reported |
| Casper 1990 | Not reported | Not reported | Not reported |
| Chen and Lukens 2011 | Together: 15.60% | Not reported | Not reported |
| Christensen et al. 2007 | Not reported | Not reported | Biological (full): 100% |
| Deal and MacLean 1995 | Together: 100% | Both siblings dependent on parent(s) or guardian(s): 100% | Not reported |
| Di Sarno et al. 2021 | Together: 100% | Primary caregiver: 100% Time spent with proband per week: Mean = 80.50 hours (*SD* = 44.98) | Not reported |
| Diaz et al. 2021 | Not reported | Not reported | Not reported |
| Fekih-Romdhane et al. 2020 | Not reported | Not reported | Biological: 100% |
| Fox et al. 2022 | Not reported | Not reported | Not reported |
| Geller et al. 2017 | Not reported | Caregiver: 100% | Not reported |
| Kovacs et al. 2016 | Not reported | Not reported | Biological (full): 100% |
| Laporte et al. 2011 | Not reported | Not reported | Biological (full): 100% |
| Lataster et al. 2010 | Together: 21.28% | Not reported | Not reported |
| Latzer et al. 2015 | Together: 100% | Not reported | Not reported |
| Mahon et al. 2013 | Not reported | Not reported | Not reported |
| Matthews et al. 2021 | Together: 100% | Dependent on parent(s) or guardian(s): 100% | Not reported |
| Meneguzzo et al. 2022 | Not reported | Not reported | Not reported |
| Modestin et al. 2008 | Not reported | Not reported | Not reported |
| Panaite et al. 2019 | Not reported | Not reported | Biological (full): 100% |
| Phillipou et al. 2022 | Not reported | Not reported | Biological: 100% |
| Phoeun et al. 2022 | Not reported | Primary caregiver: 100% | Not reported |
| Pignon et al. 2021 | Not reported | Not reported | Not reported |
| Ragazzi et al. 2020 | Not reported | Not reported | Biological: 100% |
| Reinhard and Horwitz 1995 | Together: 21.00% | Little or no caregiving invovlement: 40.00% | Not reported |
| Schick et al. 2022 | Not reported | Not reported | Not reported |
| Shivers et al. 2022 | Not reported | Not reported | Not reported |
| Sin et al. 2016 | Together: 30.00% Apart: 70.00% | Not reported | Full, half, step, or adoptive: 100% |
| Sletved et al. 2022 | Not reported | Not reported | Biological (full): 100% |
| Smith et al. 2016 | Not reported | Not reported | Not reported |
| Tanaka 2008 | Not reported | Caregiver: 28.46% (approx.) | Biological: 100% |
| Tatay-Manteiga et al. 2019 | Not reported | Not reported | Not reported |
| Taylor et al. 2008 | Not reported | Not reported | Not reported |
| van Sprang et al. 2021 | Not reported | Not reported | Biological (full): 100% |
| Zauszniewski and Bekhet 2014 | Not reported | Not reported | Not reported |
| Zhang et al. 2022 | Not reported | Not reported | Not reported |
| *Qualitative* | | | |
| Amaresha et al. 2019 | Together: 60.00% Apart: 40.00% | Caregiver: 66.70% | Biological: 100% |
| Areemit et al. 2010 | Together: 100% | Not reported | Not reported |
| Dimitropoulos et al. 2009 | Not reported | Caregiver: 100% | Biological (full): 100% |
| Ewertzon et al. 2012 | Not reported | Not reported | Not reported |
| Fjermestad et al. 2020 | Not reported | Not reported | Not reported |
| Friedrich et al. 1999 | Apart: 100% | Not reported | Not reported |
| Gerace et al. 1993 | Not reported | Not reported | Not reported |
| Hutchison et al. 2022 | Together: 100% | Not reported | Not reported |
| Jungbauer et al. 2016 | Together: 18.75% Apart: 81.25% | Not reported | Biological (full): 100% |
| Karlstad et al. 2021 | Apart: 90.00% Together: 10.00% | Not reported | Not reported |
| Kovacs et al. 2019 | Not reported | Primary caregiver: 7.14% | Not reported |
| Kristoffersen and Mustard 2000 | Not reported | Not reported | Not reported |
| Lukens et al. 1995 | Apart: 100% | Primary caregivers: 52.63% Shared responsibility with a parent: 42.11% Shared responsibility with another sibling: 5.26% | Not reported |
| Lukens et al. 2004 | Apart: 94.74% Together: 5.26% | Not reported | Not reported |
| Persico et al. 2021 | Not reported | Not reported | Not reported |
| Riebschleger 1991 | Not reported | Primary caregiver: 40.00% | Not reported |
| Samuels and Chase 1979 | Not reported | Not reported | Not reported |
| Schmid et al. 2009 | Living together: 13.50% Living apart: 86.50% | Primary caregiver: 13.50% | Not reported |
| Scutt et al. 2022 | Not reported | Not reported | Not reported |
| Sin et al. 2008 | Co-residing: 10.00% | Not reported | Not reported |
| Sin et al. 2012 | Not reported | Not reported | Not reported |
| Stålberg et al. 2004 | Not reported | Not reported | Not reported |

## **Supplementary Table 12.** Quality appraisal of reviewed studies providing quantitative data

| Study | Q1 | Q2 | Q3 | Q4 | Q5 | Q6 | Q7 | Q8 | Overall Appraisal |
| --- | --- | --- | --- | --- | --- | --- | --- | --- | --- |
| Alzahrani et al. 2017 | N | N | N | NA | NA | NA | Y | NA | Low |
| Amaresha et al. 2018 | Y | Y | Y | NA | NA | NA | Y | NA | High |
| Amianto et al. 2011 | Y | Y | Y | NA | NA | NA | Y | NA | High |
| Avcıoğlu et al. 2019 | N | Y | N | NA | NA | NA | Y | NA | Low |
| Barak and Solomon 2005 | N | N | N | NA | NA | NA | Y | NA | Low |
| Barrett et al. 2004 | Y | N | N | NA | NA | NA | Y | NA | High |
| Bowman et al. 2017 | Y | Y | Y | NA | NA | NA | Y | NA | High |
| Boyette et al. 2013 | N | Y | N | NA | NA | NA | Y | NA | High |
| Casper 1990 | N | N | Y | NA | NA | NA | Y | NA | Low |
| Chen and Lukens 2011 | Y | Y | N | NA | NA | NA | N | NA | Low |
| Christensen et al. 2007 | Y | N | Y | NA | NA | NA | Y | NA | High |
| Deal and MacLean 1995 | N | N | N | NA | NA | NA | Y | NA | Low |
| Di Sarno et al. 2021 | Y | Y | Y | NA | NA | NA | Y | NA | High |
| Diaz et al. 2021 | Y | Y | Y | NA | NA | NA | Y | NA | High |
| Fekih-Romdhane et al. 2020 | Y | N | N | NA | NA | NA | Y | NA | High |
| Fox et al. 2022 | Y | N | Y | NA | NA | NA | Y | NA | High |
| Geller et al. 2017 | N | N | N | NA | NA | NA | Y | NA | Low |
| Kovacs et al. 2016 | Y | Y | Y | NA | NA | NA | Y | NA | High |
| Laporte et al. 2011 | Y | N | Y | NA | NA | NA | Y | NA | High |
| Lataster et al. 2010 | Y | Y | Y | NA | NA | NA | Y | NA | High |
| Latzer et al. 2015 | N | N | N | NA | NA | NA | Y | NA | Low |
| Mahon et al. 2013 | Y | Y | Y | NA | NA | NA | Y | NA | High |
| Matthews et al. 2021 | Y | N | Y | NA | NA | NA | Y | NA | High |
| Meneguzzo et al. 2022 | Y | Y | Y | NA | NA | NA | Y | NA | High |
| Modestin et al. 2008 | N | Y | Y | NA | NA | NA | Y | NA | High |
| Panaite et al. 2019 | Y | N | N | NA | NA | NA | Y | NA | Low |
| Phillipou et al. 2022 | Y | N | Y | NA | NA | NA | Y | NA | High |
| Phoeun et al. 2022 | Y | N | N | NA | NA | NA | Y | NA | Low |
| Pignon et al. 2021 | N | N | Y | NA | NA | NA | Y | NA | High |
| Ragazzi et al. 2020 | Y | Y | Y | NA | NA | NA | Y | NA | High |
| Reinhard and Horwitz 1995 | N | Y | N | NA | NA | NA | Y | NA | Low |
| Schick et al. 2022 | Y | N | Y | NA | NA | NA | Y | NA | High |
| Shivers et al. 2022 | Y | N | N | NA | NA | NA | U | NA | Low |
| Sin et al. 2016 | Y | Y | N | NA | NA | NA | Y | NA | High |
| Sletved et al. 2022 | Y | Y | Y | NA | NA | NA | Y | NA | High |
| Smith et al. 2016 | Y | N | Y | NA | NA | NA | N | NA | High |
| Tanaka 2008 | N | Y | N | NA | NA | NA | N | NA | Low |
| Tatay-Manteiga et al. 2019 | Y | Y | Y | NA | NA | NA | Y | NA | High |
| Taylor et al. 2008 | Y | N | N | NA | NA | NA | Y | NA | High |
| van Sprang et al. 2021 | Y | N | Y | NA | NA | NA | Y | NA | High |
| Zauszniewski and Bekhet 2014 | N | Y | N | NA | NA | NA | Y | NA | Low |
| Zhang et al. 2022 | Y | Y | Y | NA | NA | NA | Y | NA | High |

N = ‘no’ did not meet criteria; Y = ‘yes’ did meet criteria, NA = not applicable.

## **Supplementary Table 13.** Quality appraisal of reviewed studies providing qualitative data

| Study | Q1 | Q2 | Q3 | Q4 | Q5 | Q6 | Q7 | Q8 | Q9 | Q10 | Overall Appraisal |
| --- | --- | --- | --- | --- | --- | --- | --- | --- | --- | --- | --- |
| Amaresha et al. 2019 | N | N | N | N | N | N | N | Y | Y | Y | Low |
| Areemit et al. 2010 | N | Y | Y | N | N | N | N | N | Y | N | Low |
| Dimitropoulos et al. 2009 | Y | N | Y | N | N | N | N | Y | Y | Y | Low |
| Ewertzon et al. 2012 | N | N | N | N | N | N | N | Y | Y | N | Low |
| Fjermestad et al. 2020 | N | N | N | N | N | N | N | Y | Y | Y | Low |
| Friedrich et al. 1999 | N | N | N | N | N | N | N | Y | N | N | Low |
| Gerace et al. 1993 | N | N | N | N | N | N | N | Y | Y | Y | Low |
| Hutchison et al. 2022 | Y | Y | Y | Y | Y | N | N | N | Y | Y | High |
| Jungbauer et al. 2016 | N | N | N | N | N | N | N | Y | Y | N | Low |
| Karlstad et al. 2021 | Y | Y | Y | Y | Y | N | Y | Y | Y | Y | High |
| Kovacs et al. 2019 | Y | Y | Y | Y | Y | N | N | Y | Y | Y | High |
| Kristoffersen and Mustard 2000 | Y | Y | Y | Y | Y | N | N | Y | Y | Y | High |
| Lukens et al. 1995 | Y | N | Y | N | N | N | N | Y | Y | N | Low |
| Lukens et al. 2004 | N | N | Y | N | N | N | N | Y | Y | Y | Low |
| Persico et al. 2021 | Y | Y | Y | Y | Y | N | N | Y | Y | Y | High |
| Riebschleger 1991 | N | N | N | N | N | N | N | N | N | N | Low |
| Samuels and Chase 1979 | N | N | N | N | N | N | N | N | N | N | Low |
| Scutt et al. 2022 | Y | Y | Y | Y | Y | Y | Y | Y | Y | Y | High |
| Schmid et al. 2009 | N | N | N | N | N | N | N | N | N | N | Low |
| Sin et al. 2008 | Y | Y | Y | N | Y | N | N | N | Y | N | High |
| Sin et al. 2012 | N | Y | Y | Y | N | N | N | N | Y | N | Low |
| Stålberg et al. 2004 | Y | N | Y | Y | Y | N | N | Y | N | N | Low |

N = ‘no’ did not meet criteria; Y = ‘yes’ did meet criteria, NA = not applicable.

## **Supplementary Table 14.** Summary of findings according to Grading of Recommendation, Assessment, Development and Evaluation (GRADE) guidelines

| Outcome | Participants (studies) | Quality of evidence (GRADE) | Absolute effect (95% CI) | Relative effect (95% CI) |
| --- | --- | --- | --- | --- |
| Depressive symptoms | 2,187 participants (28 studies) | ⨁ Very Low due to risk of bias^a,b^, publication bias, and inconsistency^c^ | Not applicable | 15.71 [12.99-18.43] |
| Anxiety symptoms | 1,122 participants (16 studies) | ⨁ Very Low due to risk of bias^b^ and publication bias | Not applicable | 22.45 [17.09-27.80] |
| Burden (overall) | 474 participants (five studies) | ⨁ Very Low due to risk of bias^a,b^, publication bias, imprecision^d^, and inconsistency^c^ | Not applicable | 36.27 [25.14-47.39] |
| Burden (negative) | 396 participants (four studies) | ⨁ Very Low due to risk of bias^b^, publication bias, and imprecision^d^ | Not applicable | 37.29 [19.78-54.80] |
| Burden (positive) | 396 participants (four studies) | ⨁ Very Low due to risk of bias^b^, publication bias, imprecision^d^, and inconsistency^c^ | Not applicable | 49.91 [23.10-76.72] |
| Burden (objective) | 109 participants (three studies) | ⨁ Very Low due to publication bias | Not applicable | 40.19 [34.82-45.56] |
| Burden (subjective) | 107 participants (three studies) | ⨁ Very Low due to publication bias and imprecision^d^ | Not applicable | 41.97 [31.36-52.58] |
| Hedonic wellbeing (negative affect) | 366 participants (three studies) | ⨁ Very Low due to publication bias and inconsistency^c^ | Not applicable | 14.64 [8.22-21.05] |
| Hedonic wellbeing (positive affect) | 286 participants (two studies) | ⨁ Very Low due to publication bias, imprecision^d^, and inconsistency | Not applicable | 55.64 [40.06-71.21] |
| Eudemonic wellbeing | 276 participants (three studies) | ⨁ Very Low due to risk of bias^b^, publication bias, imprecision^d^, and inconsistency^c^ | Not applicable | 69.39 [56.90-81.87] |

GRADE = Grading of Recommendation, Assessment, Development, and Evaluation; CI = confidence interval

^a^Risk of bias due to failure to develop and apply appropriate eligibility criteria.

^b^Risk of bias due to flawed measurement of exposure.

^c^Inconsistency due to unexplained heterogeneity.

^d^Imprecision due to large confidence interval.

## **Supplementary Table 15.** Materials available on request

| **Section** | **Available material** |
| --- | --- |
| Introduction | Nil |
| Methods | Template of data extraction form |
| Results | Extracted data from included studies in .xml format |
|  | Data included in meta-analyses in .cma format |
| Discussion | Nil |
| Other | Unpublished protocol for overarching systematic review |

*Note:* To request material, please contact Professor Sue Cotton at Orygen Youth Health, 35 Poplar Road, Parkville VIC 3052. Ph: (+613) 9966 9448. E: [Sue.Cotton@orygen.org.au](mailto:Sue.Cotton@orygen.org.au)

# Section Two: Quantitative Results for Depressive Symptoms

2.1 Main analysis


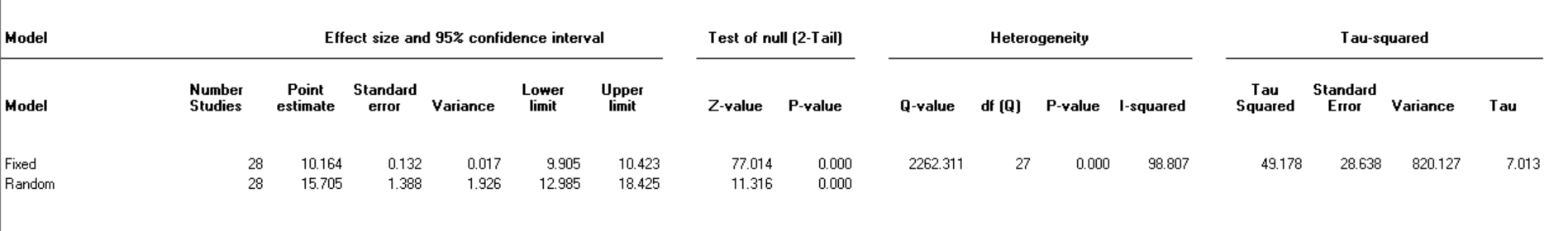


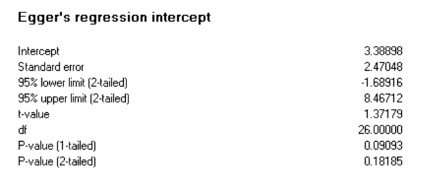


2.2 Sensitivity analysis 1: subscales and unvalidated measures removed


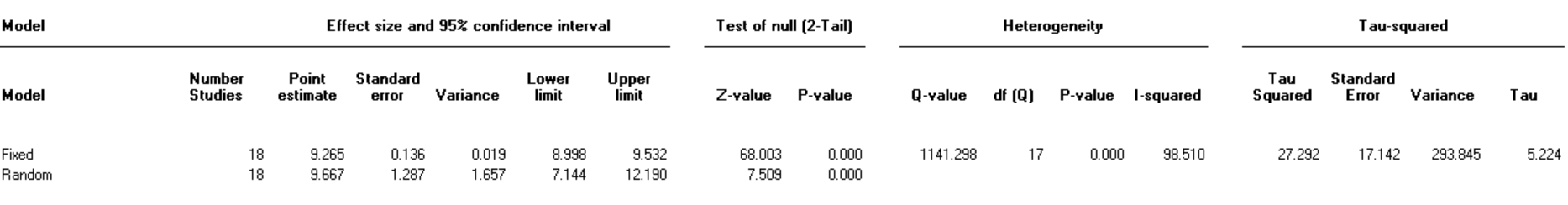


2.3 Sensitivity analysis 2: studies evaluated as ‘Low’ quality removed


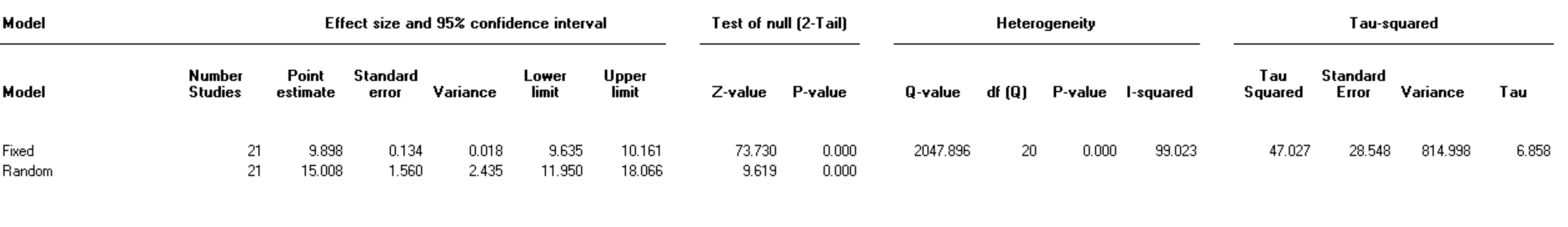


2.4 Age


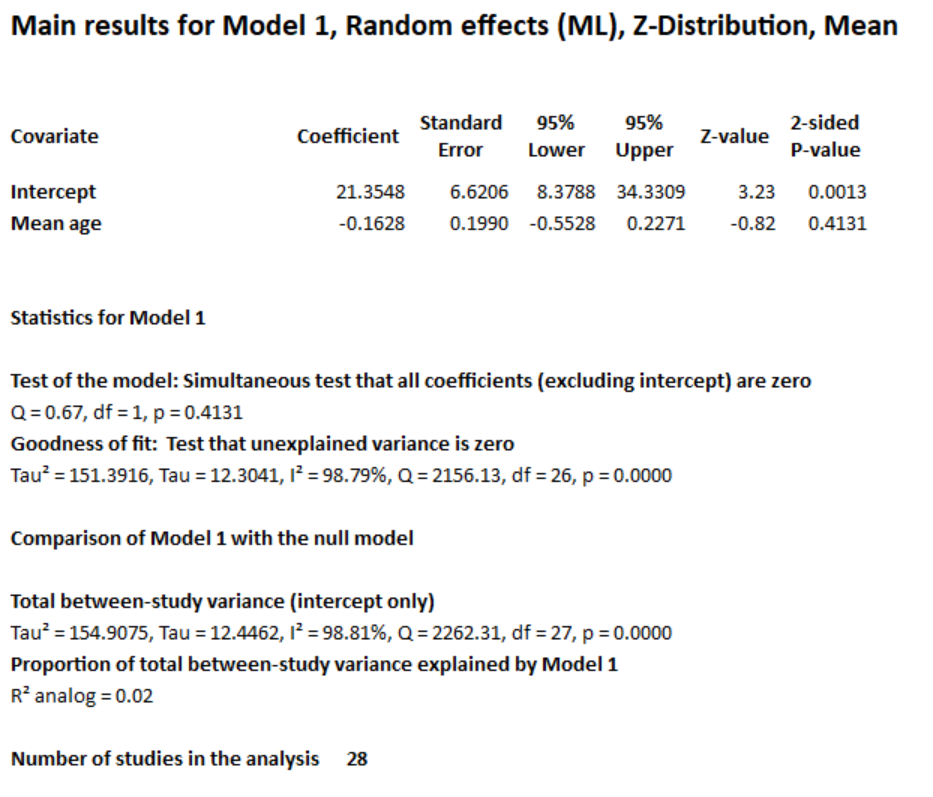


2.5 Percentage of female participants


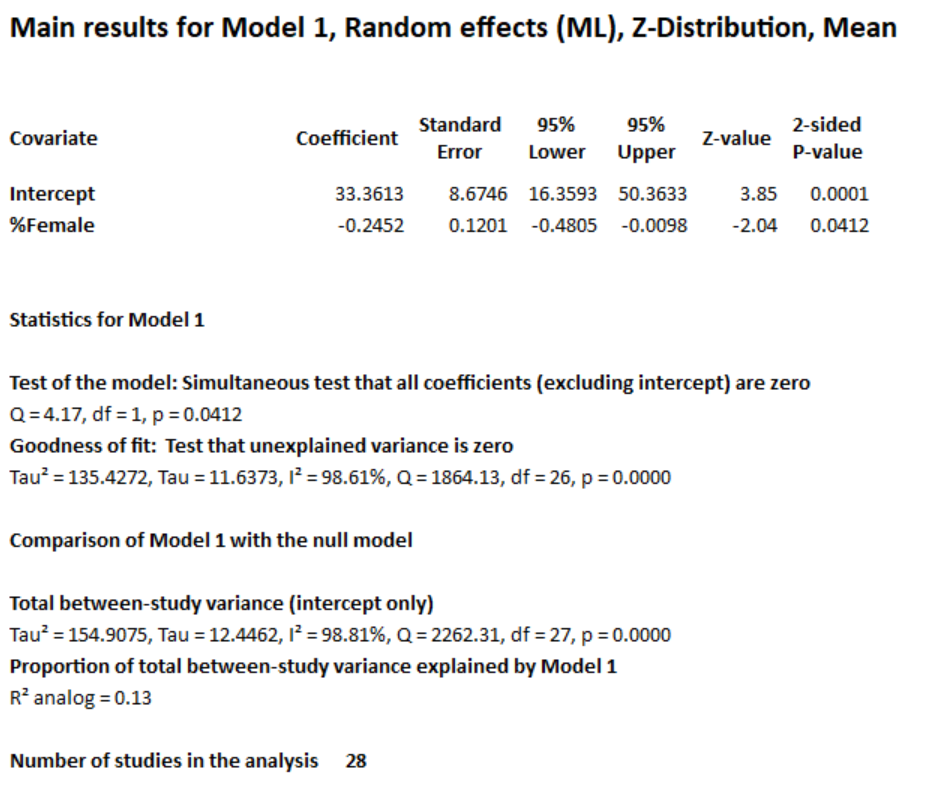

2.6 Region of study: Americas vs. Asia and Oceania vs. Europe


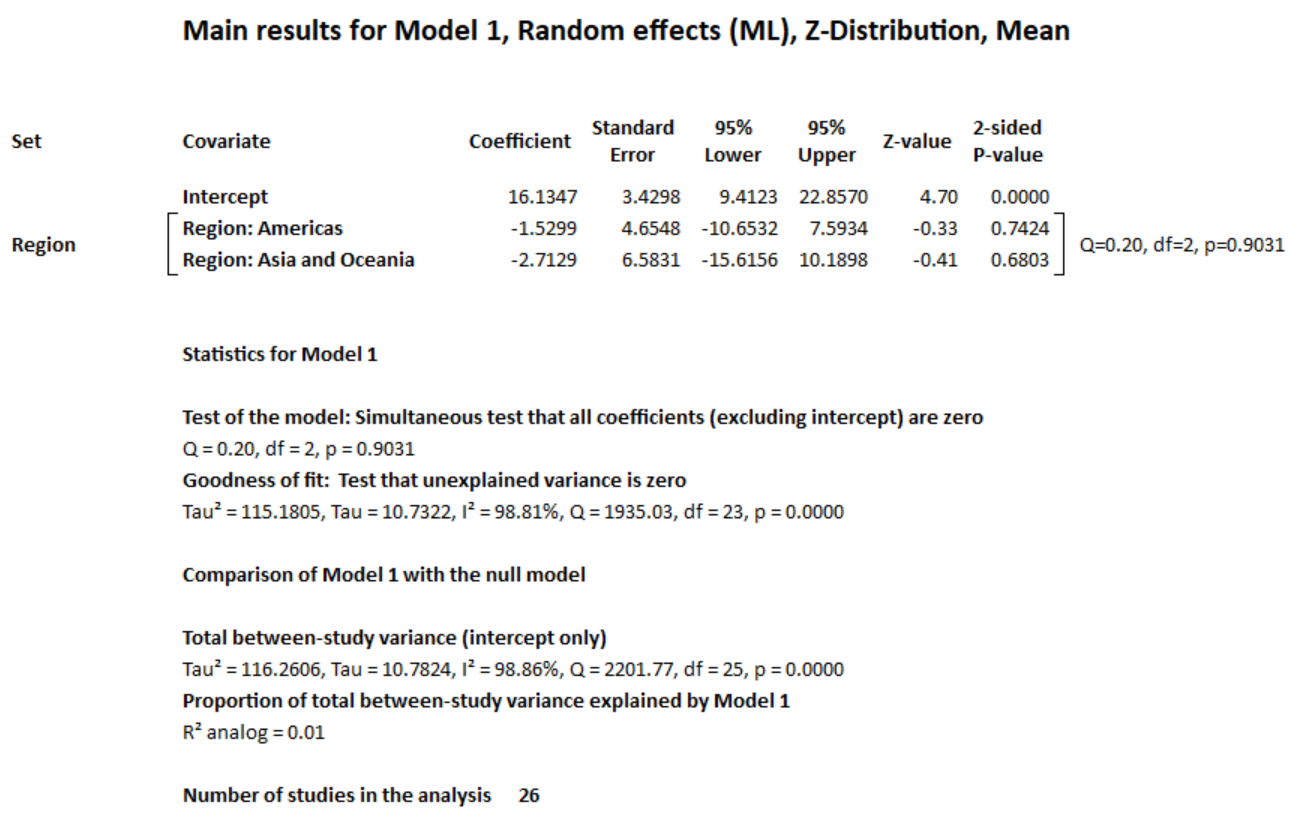


2.7 Income status of study country: high vs. not-high


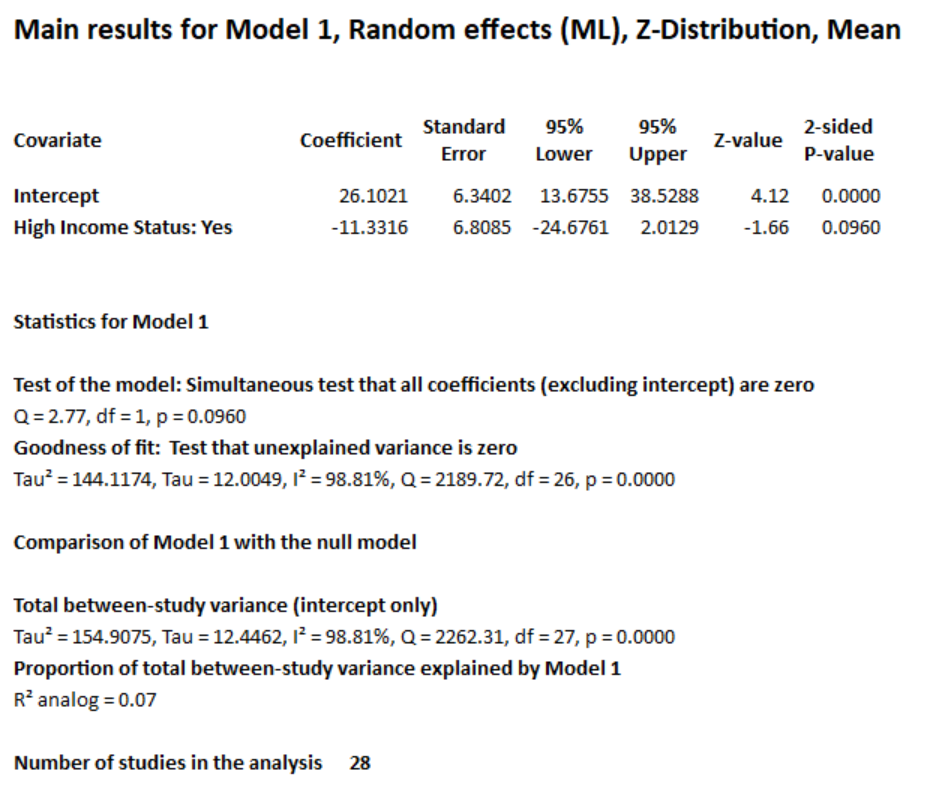


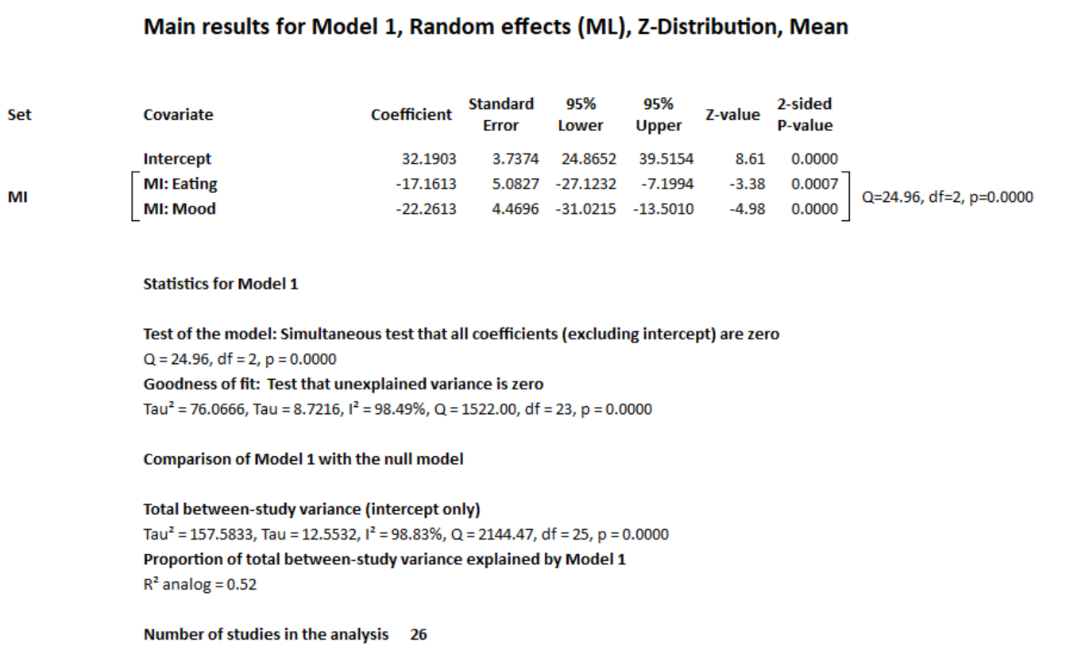
2.8 Category of mental illness: eating vs mood vs schizophrenia spectrum disorder

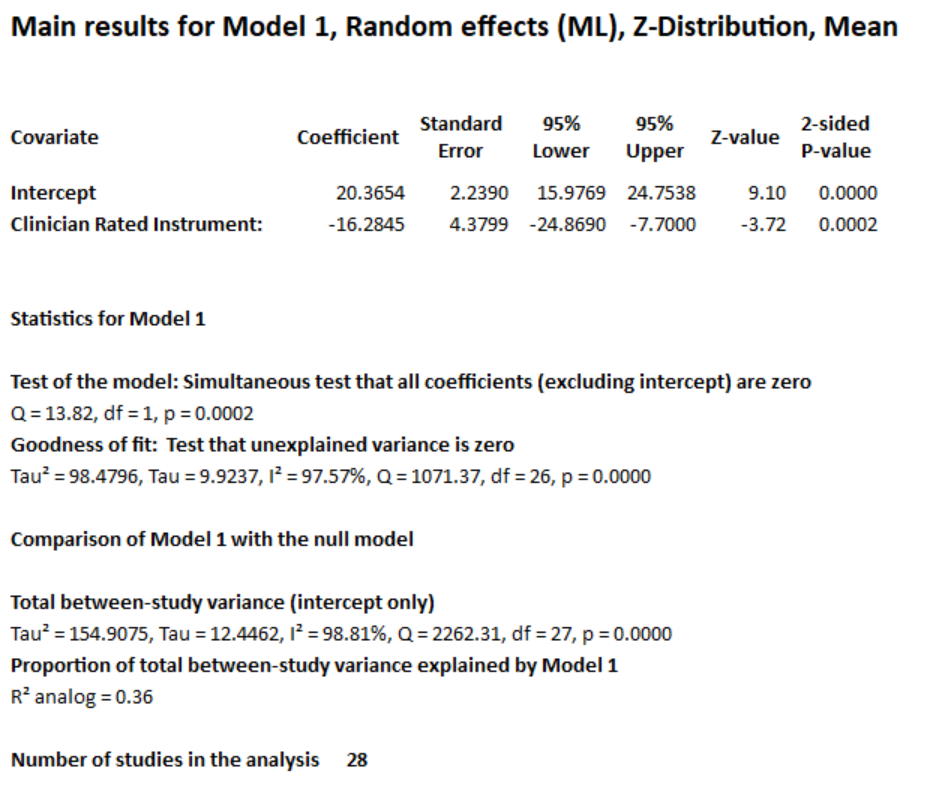
2.9 Instrument rater: clinician vs. self-rated

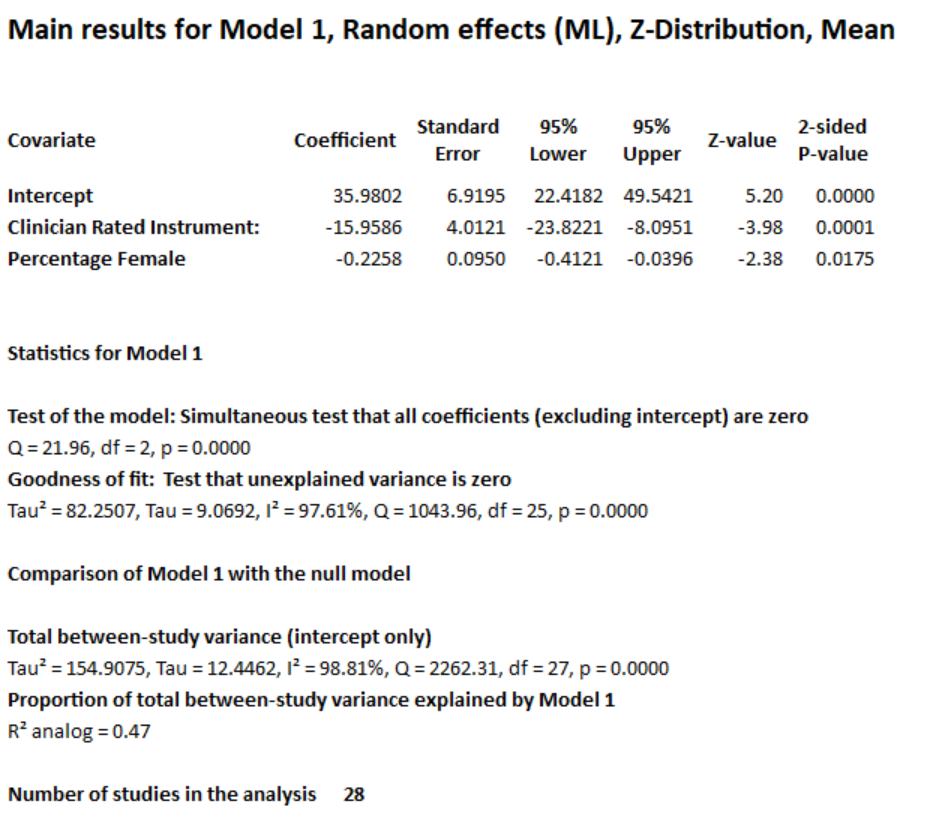
2.10 Percentage of female participants and instrument rater

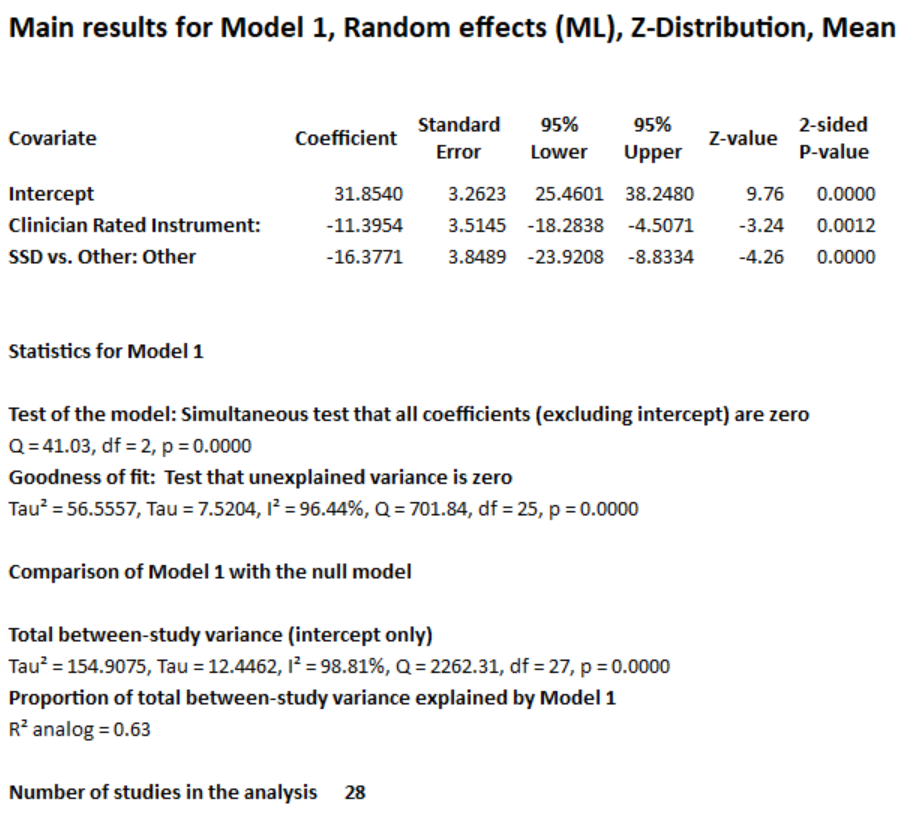
2.11 Category of mental illness (SSD vs other) and instrument rater

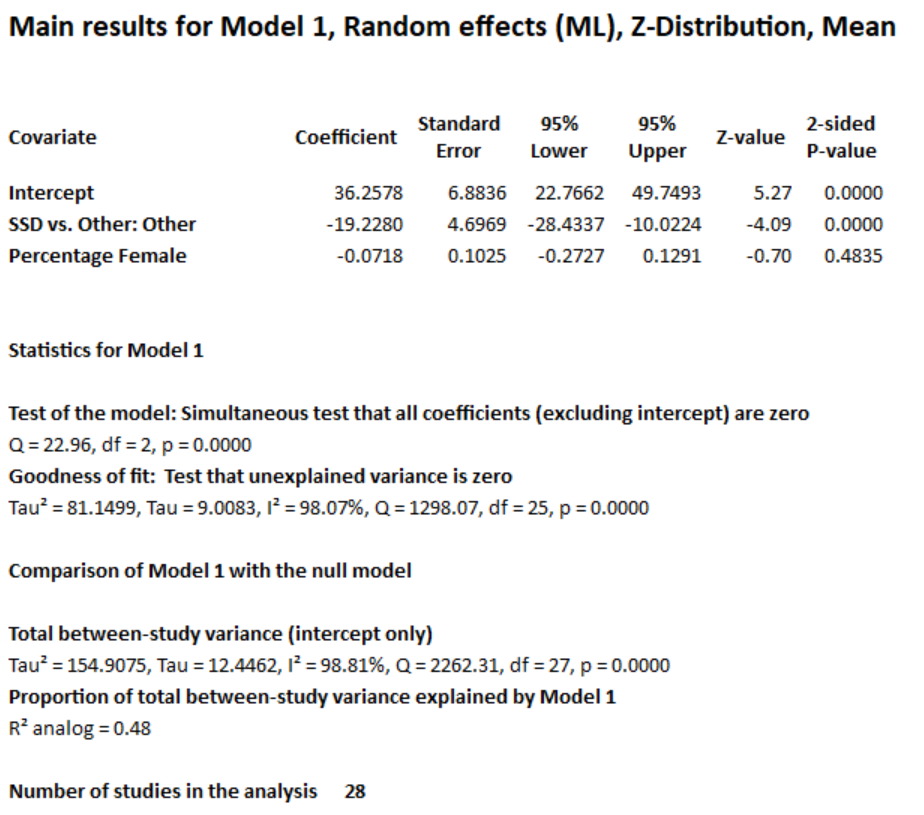
2.12 Category of mental illness and percentage female participants

2.13 Main analysis with two outlying studies removed


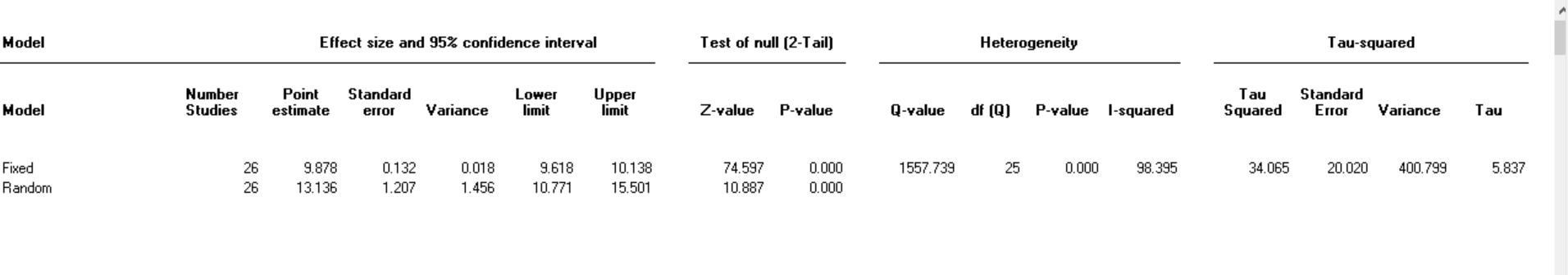


2.14 Age with two outlying studies removed


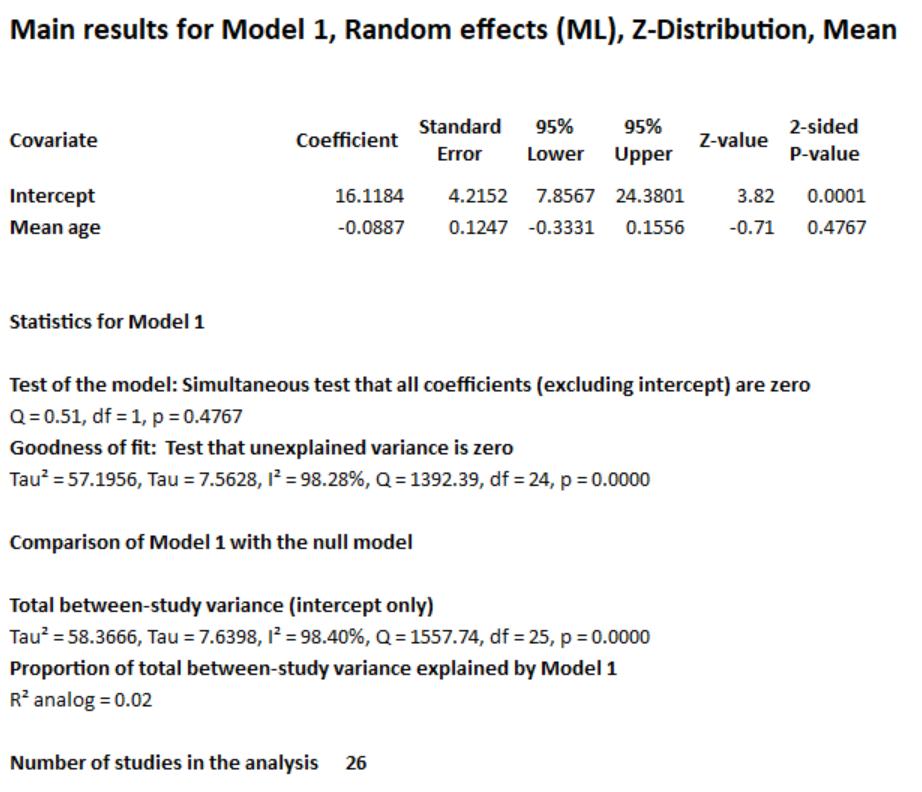


2.15 Percentage of female participants with two outlying studies removed


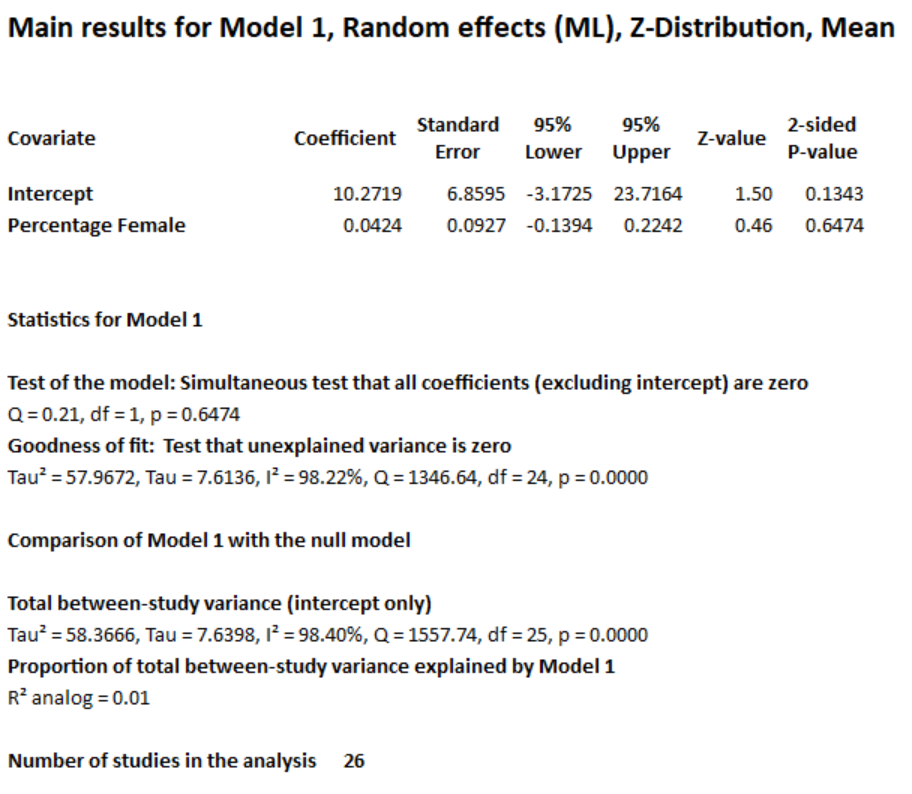


2.16 Region of study (Americas vs. Asia and Oceania vs. Europe) with two outlying studies removed


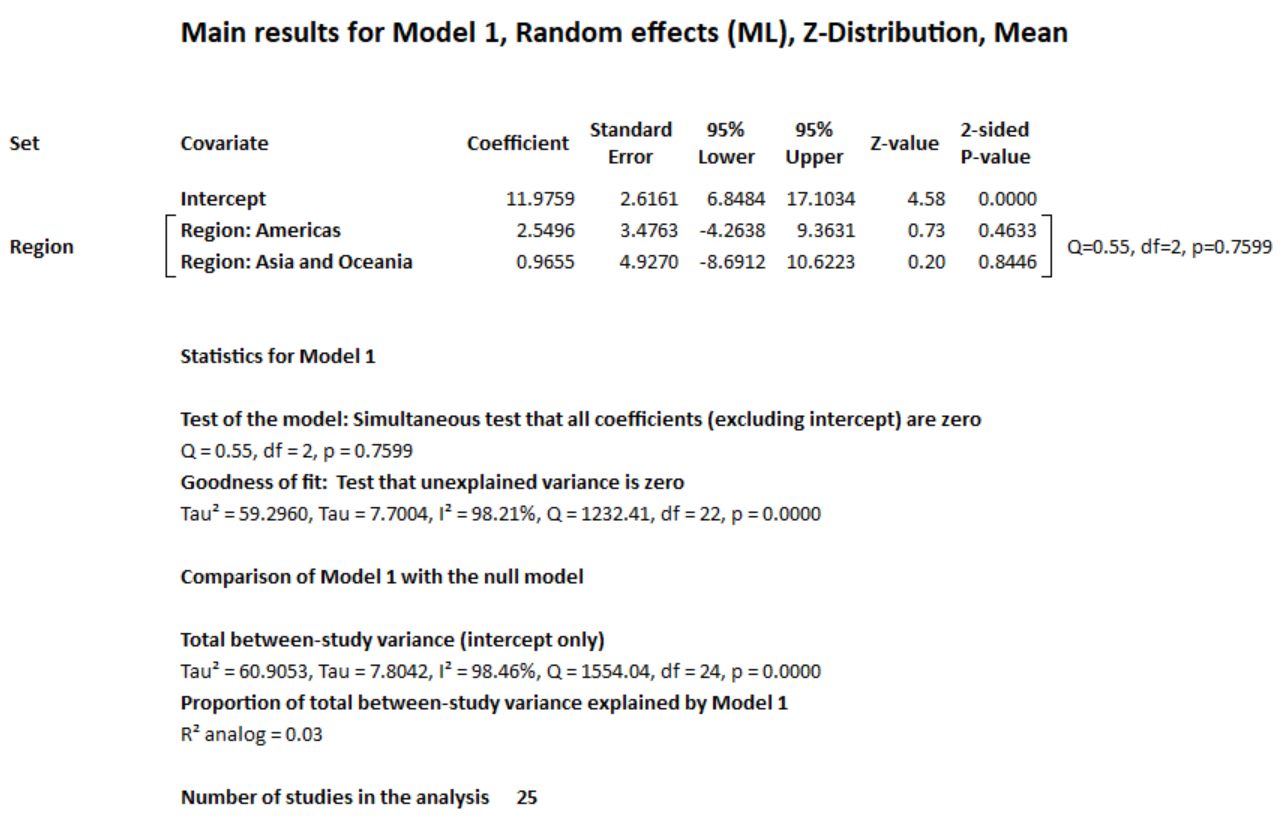


2.17 Income status of study country (high vs. not-high) with two outlying studies removed


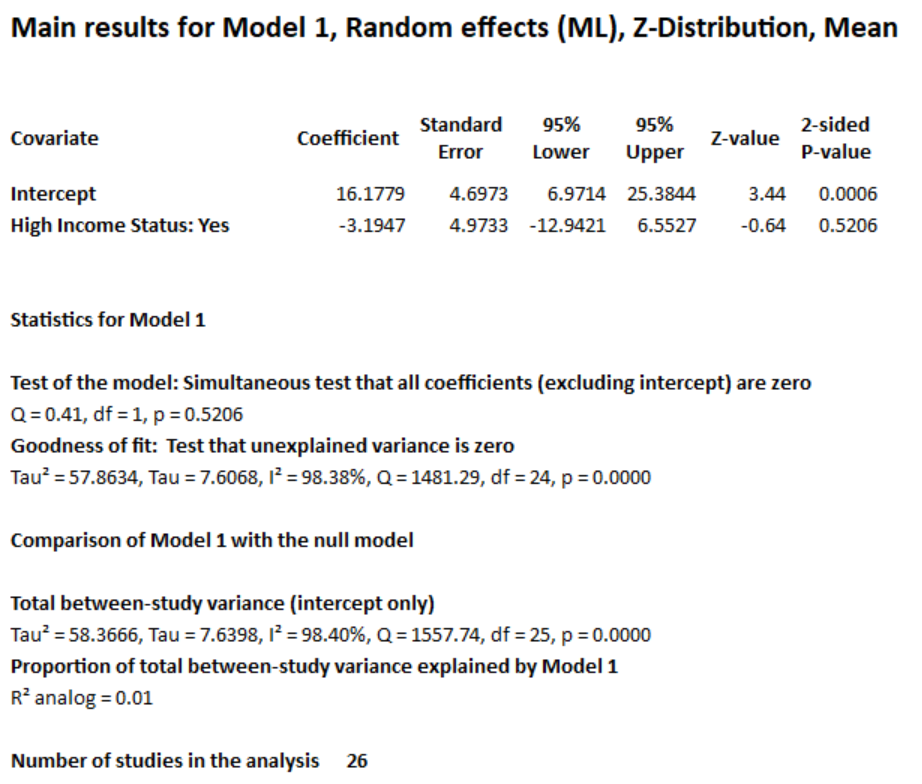


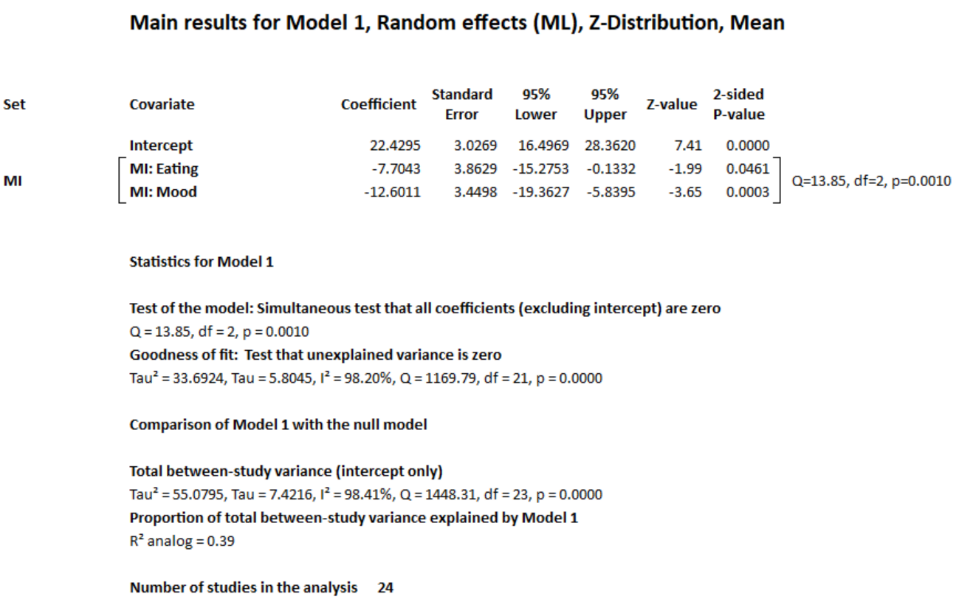
2.18 Category of mental illness (eating disorder vs. mood disorder vs. schizophrenia spectrum disorder (SSD)) with two outlying studies removed

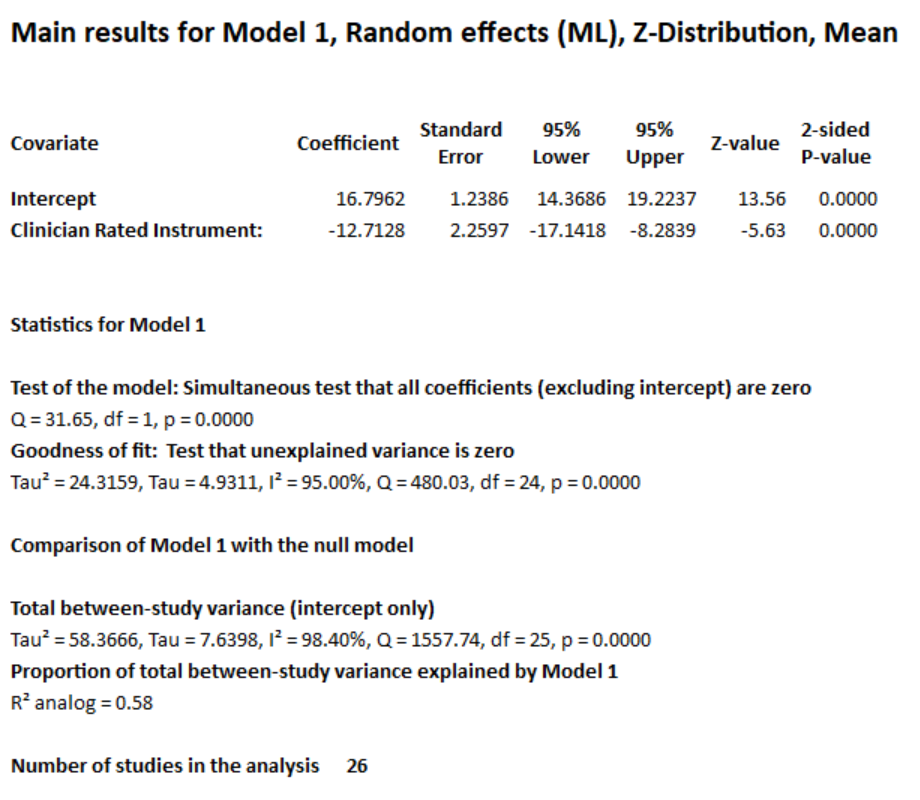
2.19 Instrument rater (clinician vs. self-rated) with two outlying studies removed

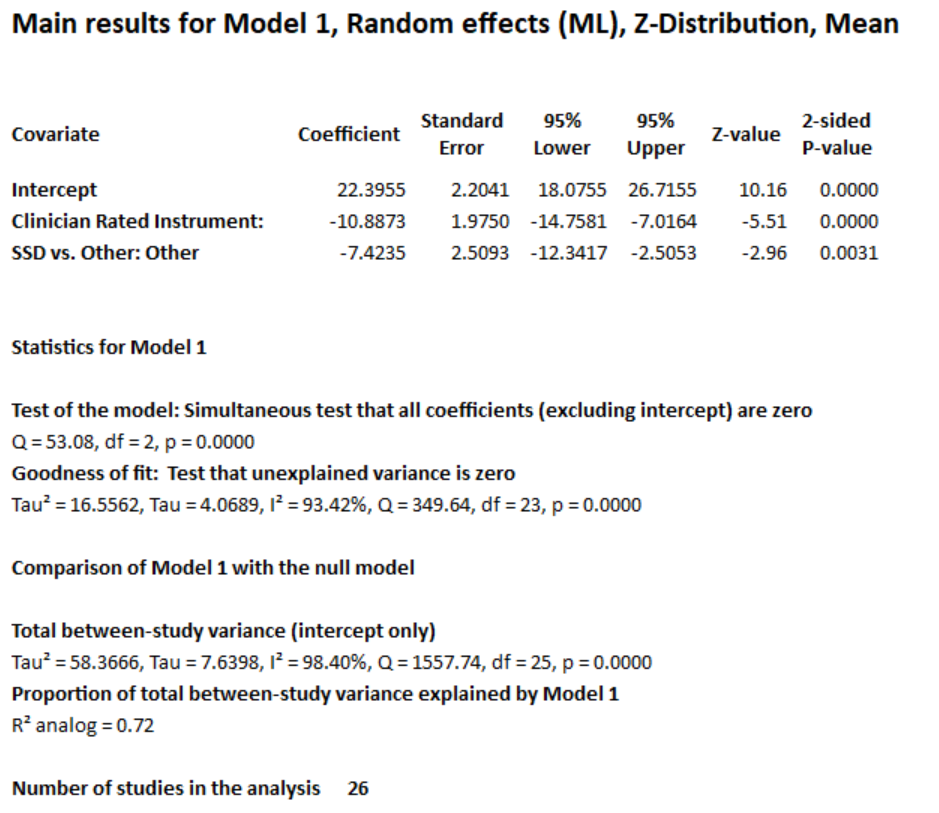
2.20 Category of mental illness (SSD vs other) and instrument rater with two outlying studies removed

# Section Three: Quantitative Results for Anxiety Symptoms

3.1 Main analysis


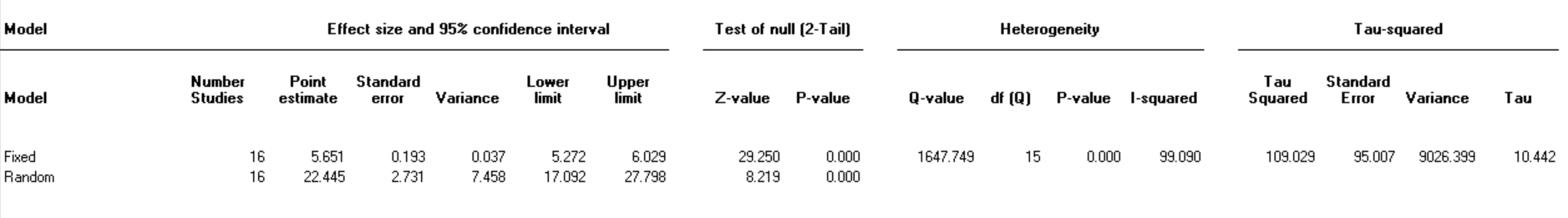


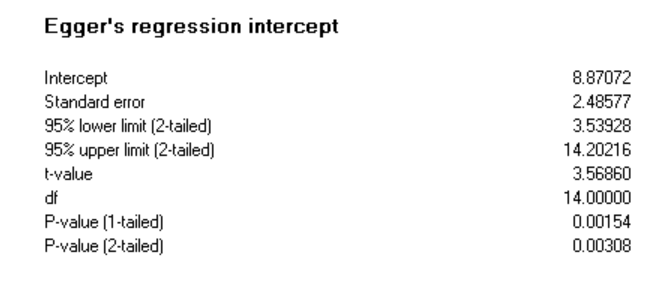


3.2 Sensitivity analysis 1: subscales and unvalidated measures removed


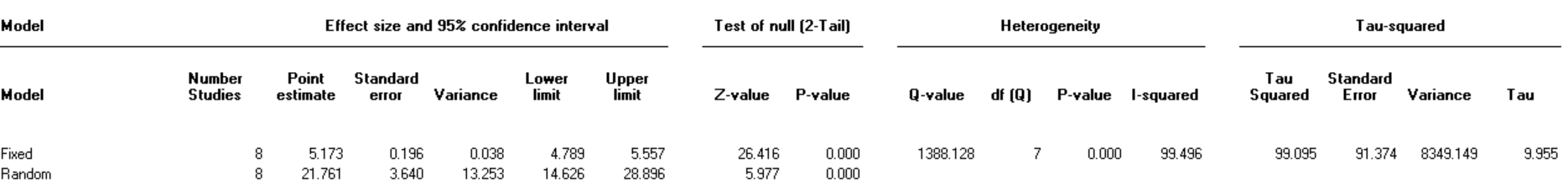


3.3 Sensitivity analysis 2: studies evaluated as ‘Low’ quality removed


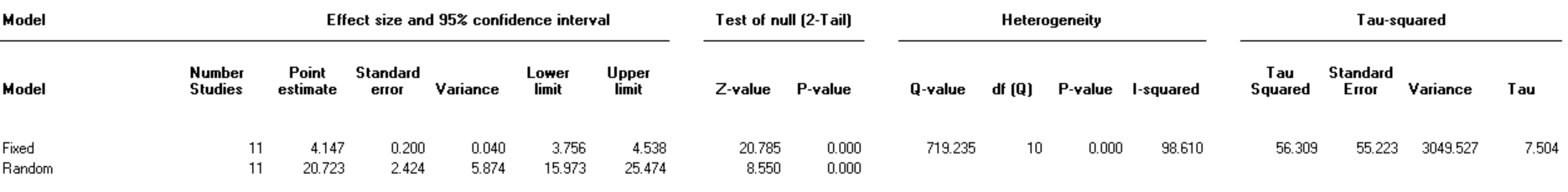


3.4 Age


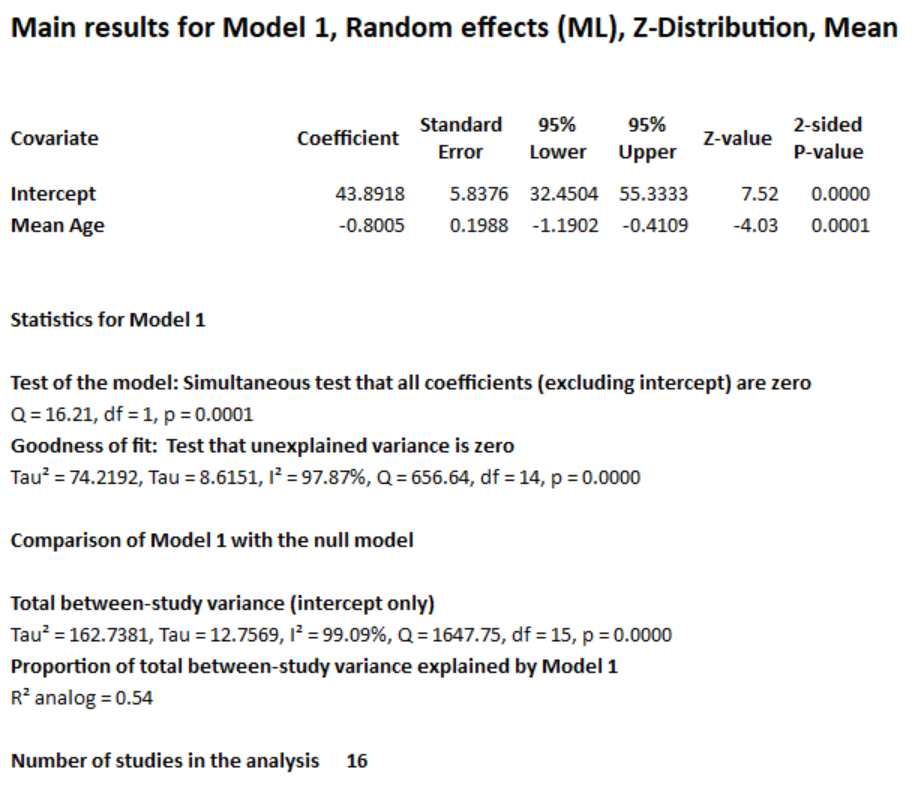


3.5 Percentage of female participants


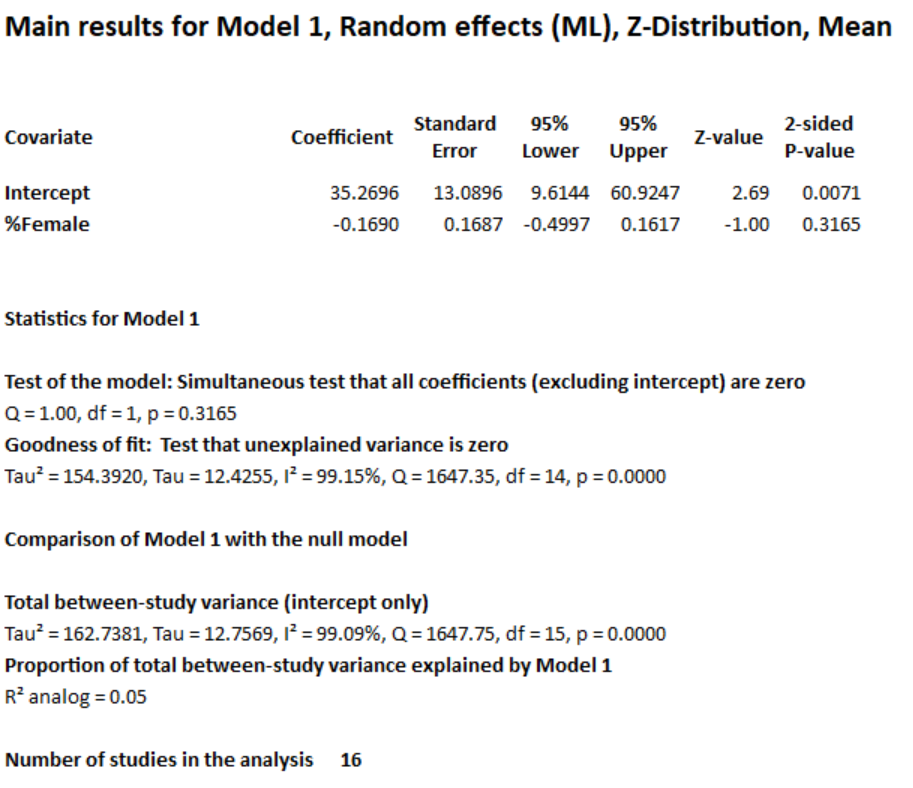


3.6 Region of study: Americas vs. Asia and Oceania vs. Europe


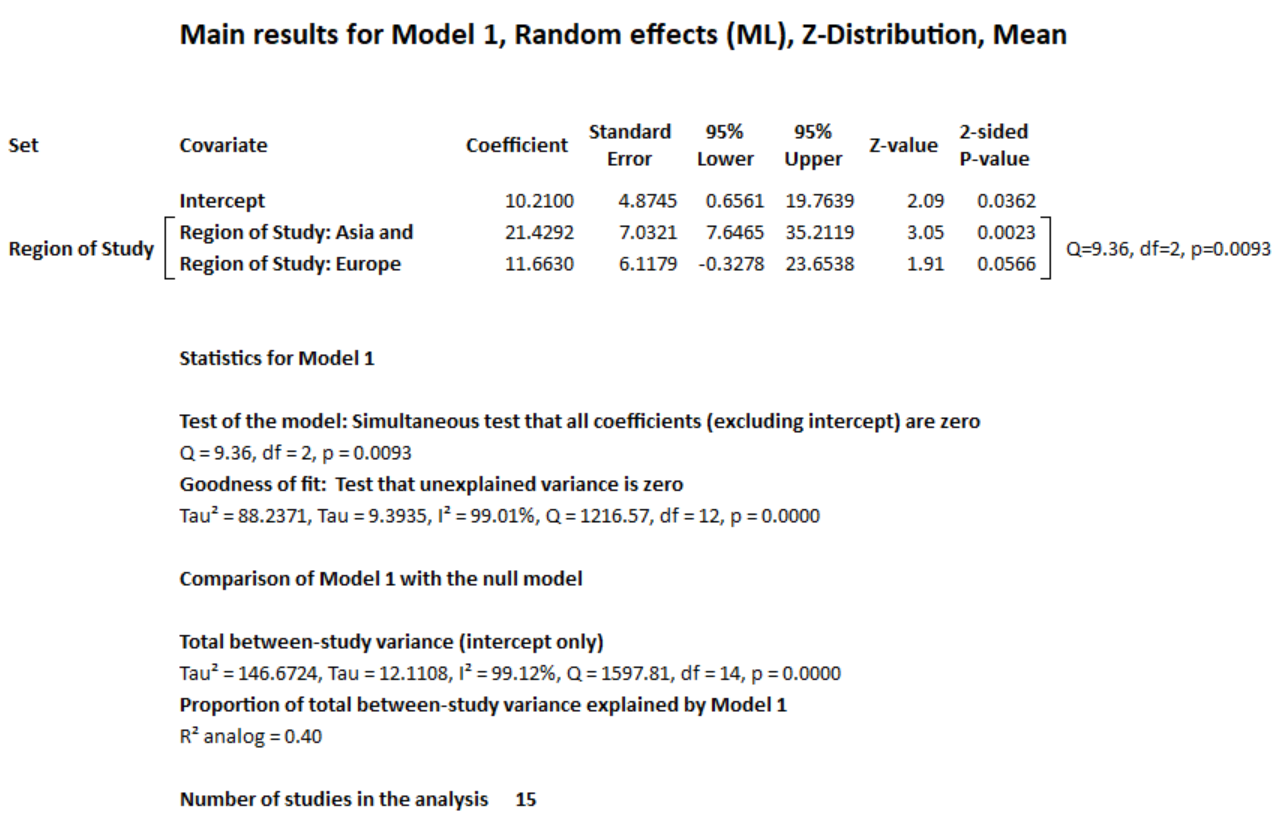

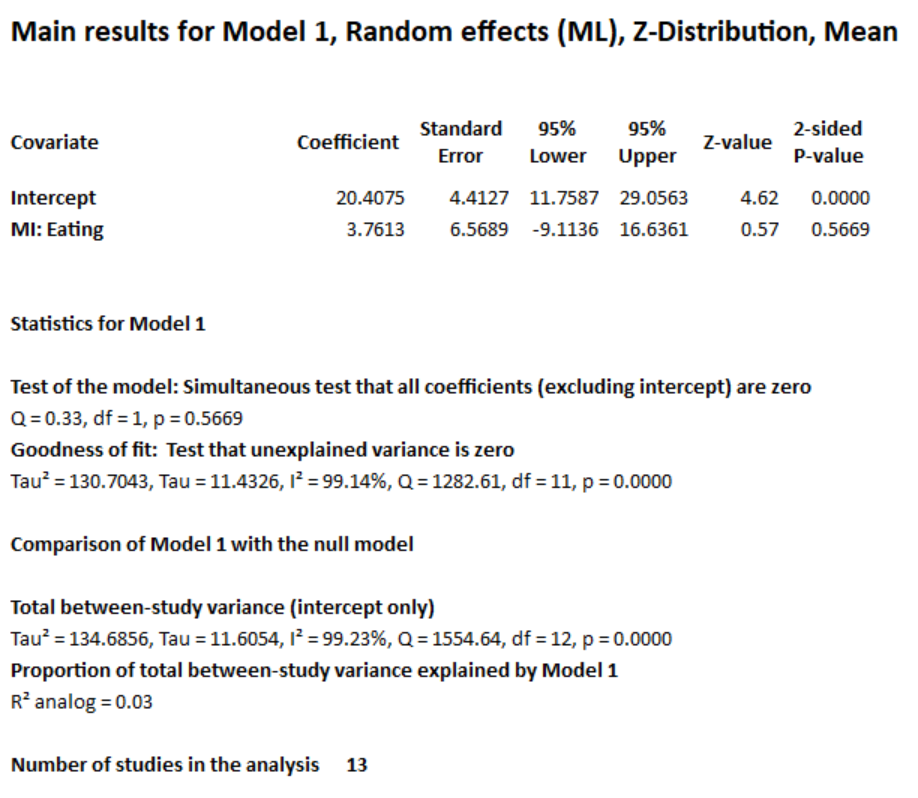
3.7 Category of mental illness: eating vs. mood disorder

# Section Four: Quantitative Results for Burden

4.1 Overall burden main analysis


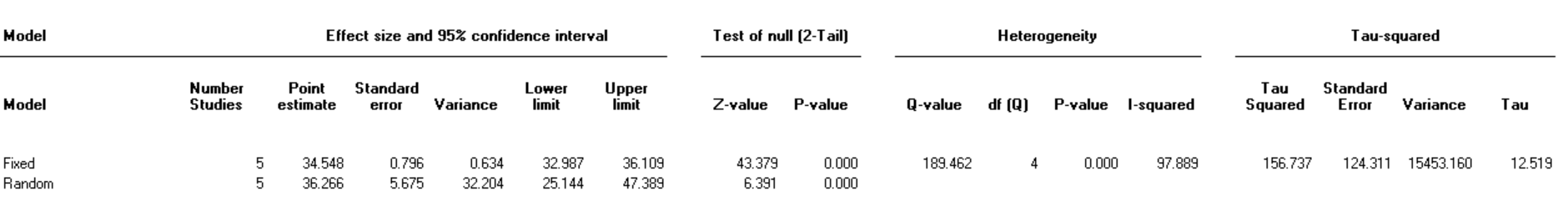


4.2 Overall burden sensitivity analysis 1: subscales and unvalidated measures removed


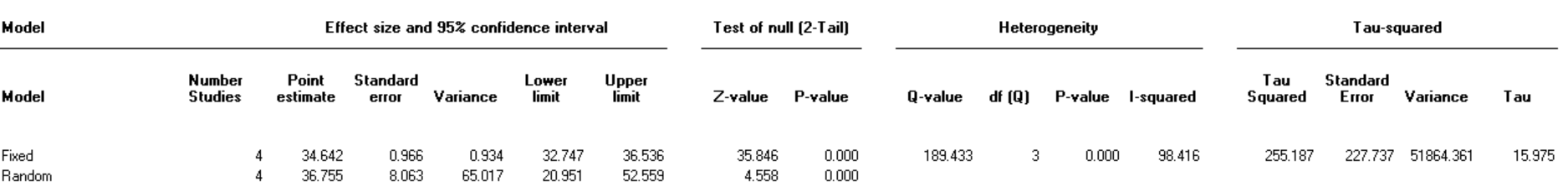


4.3 Overall burden sensitivity analysis 2: studies evaluated as ‘Low’ quality removed

*Not applicable as only one study was deemed to be of ‘high’ quality.*

4.4 Negative aspects of burden main analysis


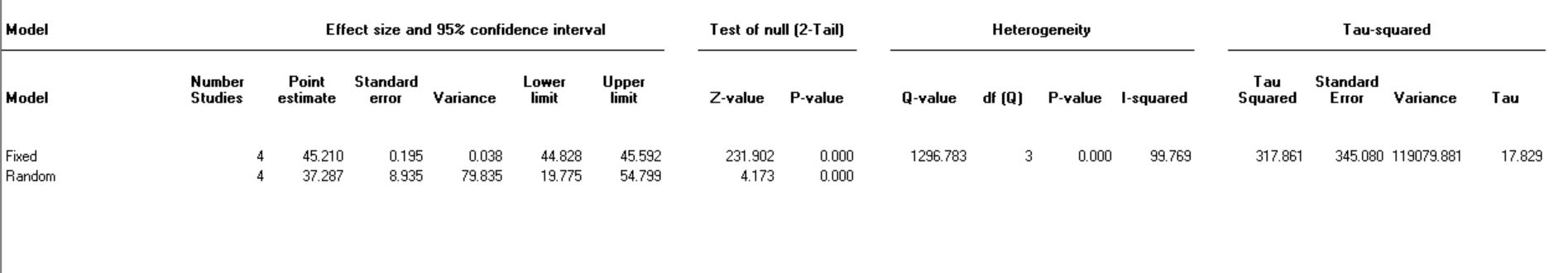

4.5 Negative aspects of burden sensitivity analysis 1: subscales and unvalidated measures removed


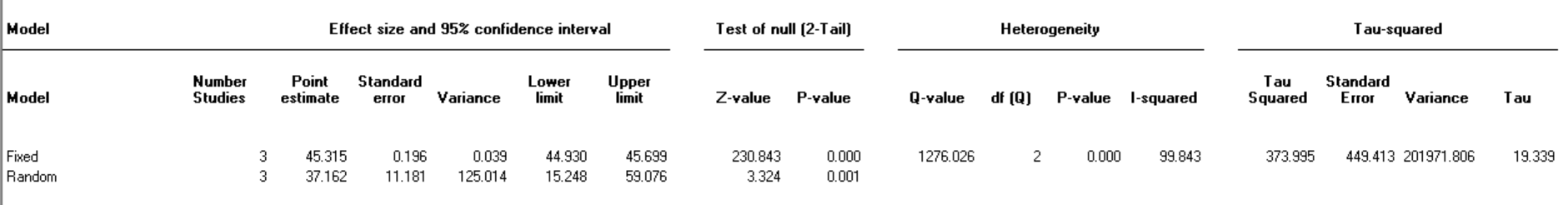


4.6 Negative aspects of burden sensitivity analysis 2: studies evaluated as ‘Low’ quality removed


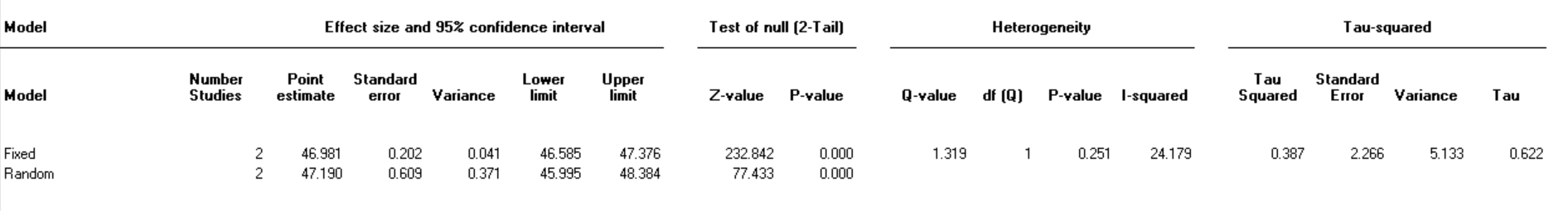


4.7 Positive aspects of burden main analysis


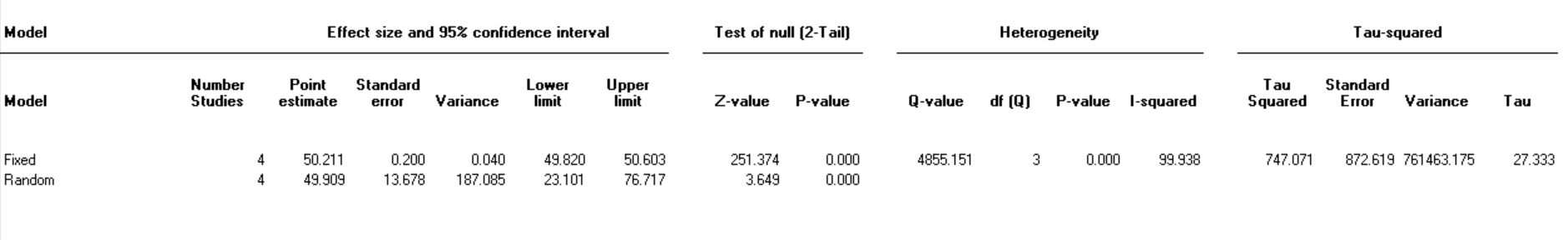

4.8 Positive aspects of burden sensitivity analysis 1: subscales and unvalidated measures removed


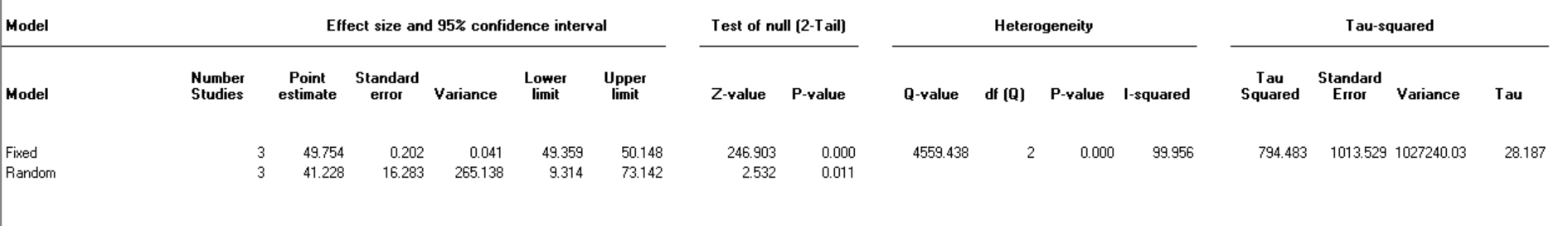


4.9 Positive aspects of burden sensitivity analysis 2: studies evaluated as ‘Low’ quality removed


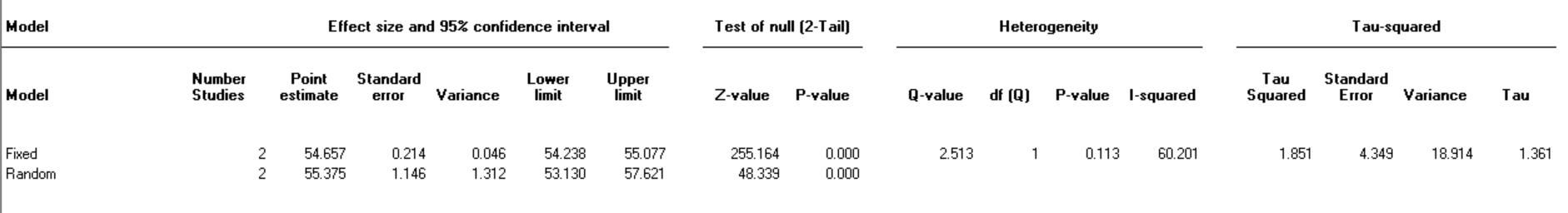


4.10 Objective burden main analysis


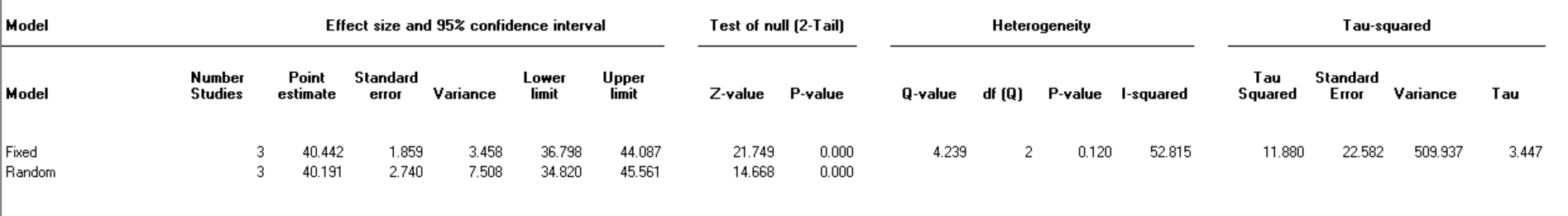

4.11 Objective burden sensitivity analysis 1: subscales and unvalidated measures removed


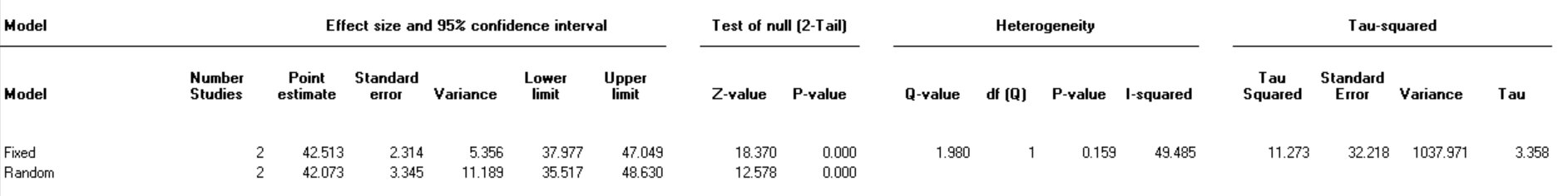


4.12 Objective burden sensitivity analysis 2: studies evaluated as ‘Low’ quality removed


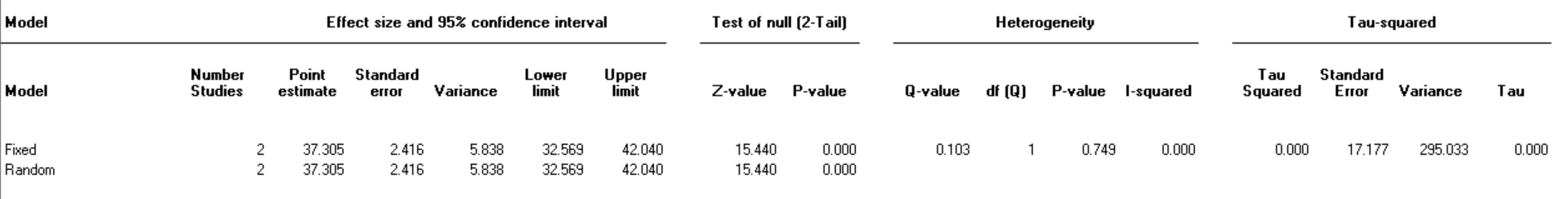


4.13 Subjective burden main analysis


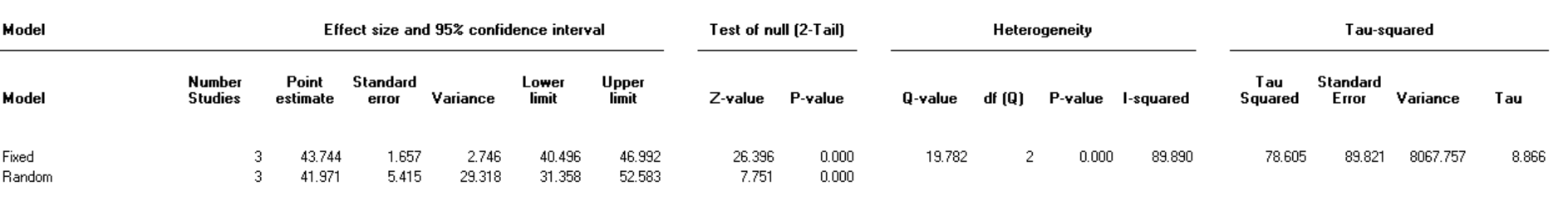

4.14 Subjective burden sensitivity analysis 1: subscales and unvalidated measures removed


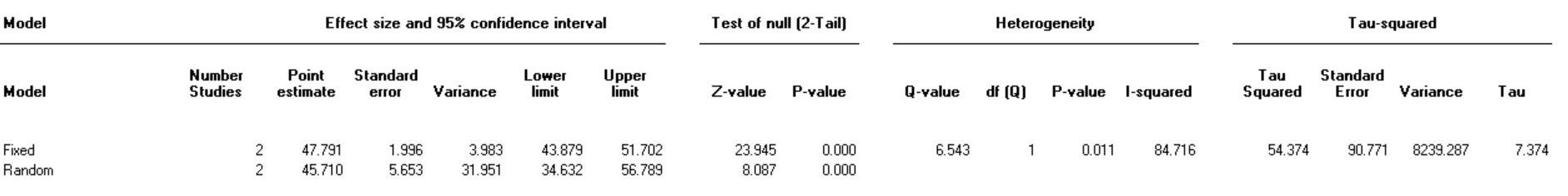


4.15 Subjective burden sensitivity analysis 2: studies evaluated as ‘Low’ quality removed


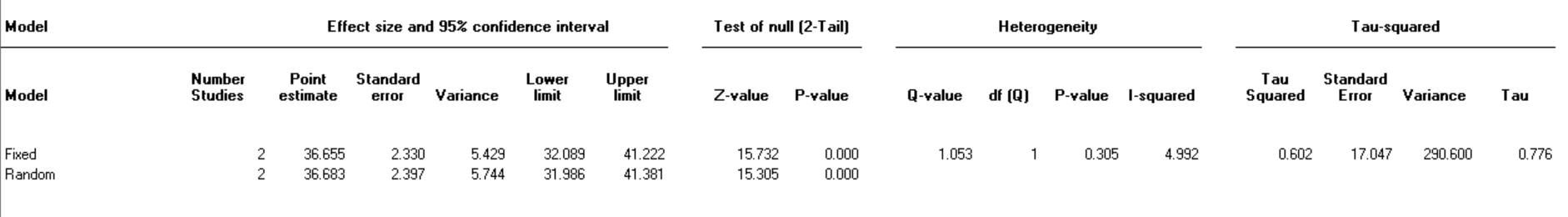


# Section Five: Quantitative Results for Wellbeing

4.1 Wellbeing (negative affect) main analysis


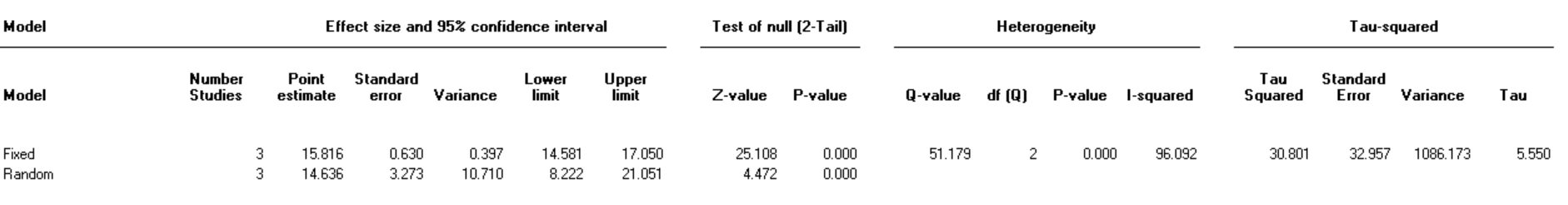

4.2 Wellbeing (negative affect) sensitivity analysis 1: subscales and unvalidated measures removed


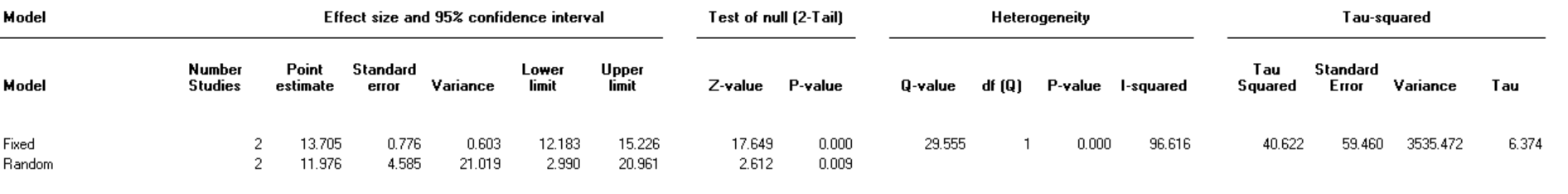


4.3 Wellbeing (negative affect) sensitivity analysis 2: studies evaluated as ‘Low’ quality removed

*Not applicable as all studies were deemed to be of ‘high’ quality.*

4.4 Wellbeing (positive affect) main analysis


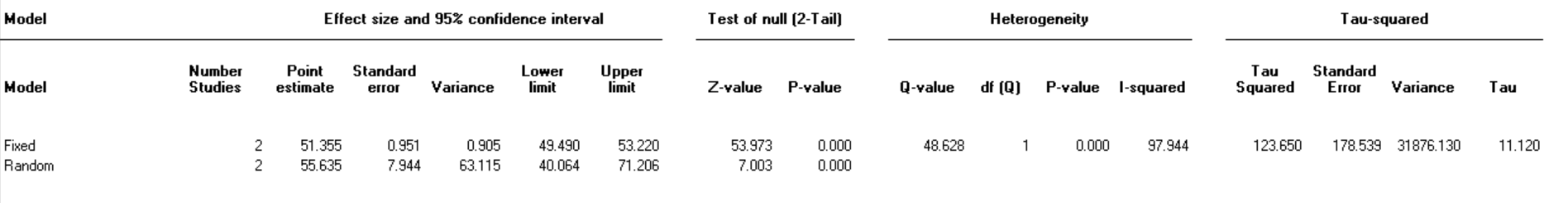

4.5 Wellbeing (positive affect) sensitivity analysis 1: subscales and unvalidated measures removed

*Not applicable as only two studies were included in analyses.*

4.6 Wellbeing (positive affect) sensitivity analysis 2: studies evaluated as ‘Low’ quality removed

*Not applicable as only two studies were included in analyses.*

4.7 Wellbeing (eudemonic) main analysis


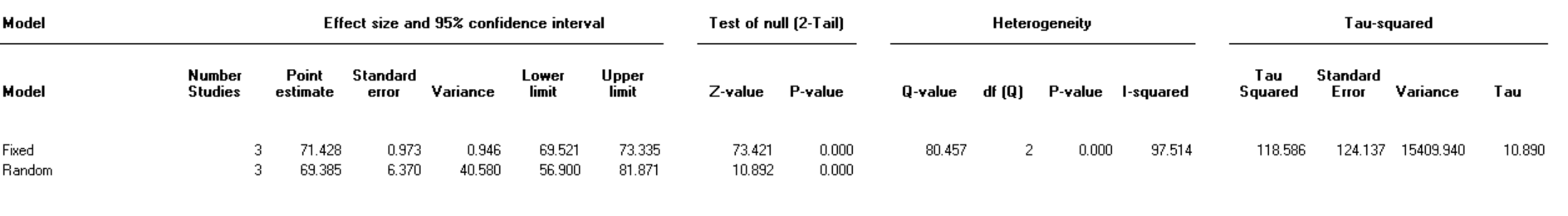


4.8 Wellbeing (eudemonic) sensitivity analysis 1: subscales and unvalidated measures removed

*Not applicable as all studies employed a validated measure.*

4.9 Wellbeing (eudemonic) sensitivity analysis 2: studies evaluated as ‘Low’ quality removed


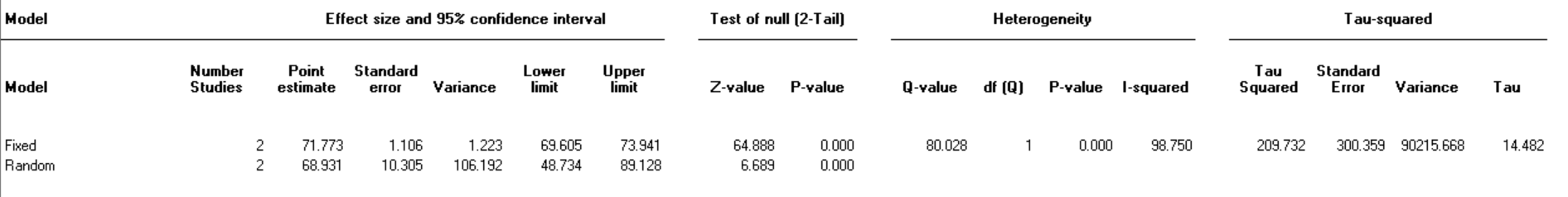


# Section Six: References

APA. (2013). *Diagnostic and Statistical Manual of Mental Disorders, Fifth Edition*. Arlington, VA: American Psychiatric Assocation.

Australian Bureau of Statistics. (2016, May 19). Standard Australian Classification of Countries, 2016. Retrieved from <https://www.abs.gov.au/AUSSTATS/abs@.nsf/Lookup/1269.0Main+Features1Second%20Edition?OpenDocument>

Avcıoğlu, M. M., Karanci, A. N., & Soygur, H. (2019). What Is Related to the Well-Being of the Siblings of Patients with Schizophrenia: An Evaluation within the Lazarus and Folkman's Transactional Stress and Coping Model. *The International journal of social psychiatry, 65*(3), 252-261.

Barnable, A., Gaudine, A., Bennett, L., & Meadus, R. (2006). Having a Sibling with Schizophrenia: A Phenomenological Study. *Research and theory for nursing practice, 20*(3), 247-264.

Bath, P. A., Deeg, D., & Poppelaars, J. (2010). The Harmonisation of Longitudinal Data: A Case Study Using Data from Cohort Studies in the Netherlands and the United Kingdom. *Ageing and Society, 30*(8), 1419-1437.

Begovic, E., Panaite, V., Bylsma, L. M., George, C., Kovacs, M., Yaroslavsky, I., . . . Rottenberg, J. (2017). Positive Autobiographical Memory Deficits in Youth with Depression Histories and Their Never-Depressed Siblings. *British Journal of Clinical Psychology, 56*(3), 329-346.

Bo, Q., Dong, F., Li, X., Li, F., Li, P., Yu, H., . . . Ma, X. (2019). Comparison of Cognitive Performance in Bipolar Disorder, Major Depressive Disorder, Unaffected First‐Degree Relatives, and Healthy Controls. *Psychiatry and clinical neurosciences, 73*(2), 70-76.

Borenstein, M., Hedges, L., Higgins, J., & Rothstein, H. (2013). Comprehensive Meta-Analysis, 3rd Ed. Englewood, NJ: Biostat.

Boyette, L. L., Korver-Nieberg, N., Verweij, K., Meijer, C., Dingemans, P., Cahn, W., . . . Myin-Germeys, I. (2013). Associations between the Five-Factor Model Personality Traits and Psychotic Experiences in Patients with Psychotic Disorders, Their Siblings and Controls. *Psychiatry Research, 210*(2), 491-497.

Callio, C., & Gustafsson, S. A. (2016). Living with a Sibling Who Suffers from an Eating Disorder: A Pilot Interview Study. *Journal of Multidisciplinary Healthcare, 9*, 615-622.

Christensen, M. V., Kyvik, K. O., & Kessing, L. V. (2007). Subclinical Psychopathology and Socio-Economic Status in Unaffected Twins Discordant for Affective Disorder. *Journal of psychiatric research, 41*(3-4), 229-238.

de Crom, S. A. M., Haan, L. d., & Schirmbeck, F. (2021). The Association between Sleep Disturbances and Negative Symptom Severity in Patients with Non-Affective Psychotic Disorders, Unaffected Siblings and Healthy Controls. *Psychiatry Research, 297*(113728). doi:10.1016/j.psychres.2021.113728

de Kluiver, H., Milaneschi, Y., Jansen, R., van Sprang, E. D., Giltay, E. J., Hartman, C. A., & Penninx, B. W. J. H. (2021). Associations between Depressive Symptom Profiles and Immunometabolic Characteristics in Individuals with Depression and Their Siblings. *The World Journal of Biological Psychiatry, 22*(2), 128-138. doi:10.1080/15622975.2020.1761562

Di Sarno, E., Napolitano, I., & Louzã, M. R. (2021). Burden on Caregivers of Schizophrenia Outpatients in Brazil: Relationship to Symptomatology and Functioning. *The International journal of social psychiatry, 68*(8), 1552-1560.

Diaz, A. P., Fernandes, B. S., Teixeira, A. L., Mwangi, B., Hasan, K. M., Wu, M., . . . Brunoni, A. R. (2021). White Matter Microstructure Associated with Anhedonia among Individuals with Bipolar Disorders and High-Risk for Bipolar Disorders. *Journal of Affective Disorders, 300*, 91-98.

Farmer, A., Redman, K., Harris, T., Webb, R., Mahmood, A., Sadler, S., & McGuffin, P. (2001). The Cardiff Sib-Pair Study. *Crisis: The Journal of Crisis Intervention and Suicide Prevention, 22*(2), 71.

Fekih-Romdhane, F., Nsibi, T., Sassi, H., & Cheour, M. (2020). Link between Childhood Trauma and Psychotic-Like Experiences in Non-Affected Siblings of Schizophrenia Patients: A Case-Control Study. *Early Intervention in Psychiatry, 15*(5), 1154-1166.

Fox, T. L., Barrett, P. M., & Shortt, A. L. (2002). Sibling Relationships of Anxious Children: A Preliminary Investigation. *Journal of Clinical Child and Adolescent Psychology, 31*(3), 375-383.

Garley, D., & Johnson, B. (1994). Siblings and Eating Disorders: A Phenomenological Perspective. *Journal of psychiatric and mental health nursing, 1*(3), 157-164.

Geller, J., Srikameswaran, S., Iyar, M., Zelichowska, J., Thibodeau, M., Brown, K. E., & Dunn, E. C. (2017). Support Stance in Carers of Adults with Eating Disorders: Factors Associated with Collaborative Versus Directive Approaches. *International Journal of Eating Disorders, 50*(5), 498-505.

Greenberg, J. S., Kim, H. W., & Greenley, J. R. (1997). Factors Associated with Subjective Burden in Siblings of Adults with Severe Mental Illness. *The American journal of orthopsychiatry, 67*(2), 231-241.

Higgins, J., Thomas, J., Chandler, J., Cumpston, M., Li, T., Page, M., & Welch, V. (2022, August 4). *Cochrane Handbook for Systematic Reviews of Interventions*. Retrieved from <www.training.cochrane.org/handbook>

Horwitz, A. V., & Reinhard, S. C. (1995). Ethnic Differences in Caregiving Duties and Burdens among Parents and Siblings of Persons with Severe Mental Illnesses. *Journal of health and social behavior, 36*(2), 138-150.

Hsiao, C. Y., & Tsai, Y. F. (2014). Caregiver Burden and Satisfaction in Families of Individuals with Schizophrenia. *Nursing research, 63*(4), 260-269. doi:10.1097/NNR.0000000000000047

Hsiao, C. Y., & Tsai, Y. F. (2015). Factors of Caregiver Burden and Family Functioning among Taiwanese Family Caregivers Living with Schizophrenia. *Journal of clinical nursing, 24*(11-12), 1546-1556. doi:10.1111/jocn.12745

Hutchison, S., House, J., McDermott, B., Simic, M., Baudinet, J., & Eisler, I. (2022). Silent Witnesses: The Experience of Having a Sibling with Anorexia Nervosa. *Journal of eating disorders, 10*(1). doi:10.1186/s40337-022-00655-1

IBM Corp. (2020). IBM SPSS Statistics for Windows, Version 27.0. Armonk, NY: IBM Corp.

Kovacs, M., Bylsma, L. M., Yaroslavsky, I., Rottenberg, J., George, C. J., Kiss, E., . . . Kapornai, K. (2016). Positive Affectivity Is Dampened in Youths with Histories of Major Depression and Their Never-Depressed Adolescent Siblings. *Clinical Psychological Science, 4*(4), 661-674.

Laporte, L., Paris, J., Guttman, H., & Russell, J. (2011). Psychopathology, Childhood Trauma, and Personality Traits in Patients with Borderline Personality Disorder and Their Sisters. *Journal of personality disorders, 25*(4), 448-462.

Latzer, Y., Ben-Ari, A., & Galimidi, N. (2002). Anorexia Nervosa and the Family: Effects on Younger Sisters to Anorexia Nervosa Patients. *International journal of adolescent medicine and health, 14*(4), 275-281.

Latzer, Y., Katz, R., & Berger, K. (2015). Psychological Distress among Sisters of Young Females with Eating Disorders: The Role of Negative Sibling Relationships and Sense of Coherence. *Journal of Family Issues, 36*(5), 626-646.

Leith, J. E., Jewell, T. C., & Stein, C. H. (2018). Caregiving Attitudes, Personal Loss, and Stress-Related Growth among Siblings of Adults with Mental Illness. *Journal of Child and Family Studies, 27*(4), 1193-1206. doi:10.1007/s10826-017-0965-4

Li, L. Z., Bian, J. Y., & Wang, S. (2021). Moving Beyond Family: Unequal Burden across Mental Health Patients’ Social Networks. *Quality of Life Research, 30*, 1873-1879.

Liegghio, M. (2017). ‘Not a Good Person’: Family Stigma of Mental Illness from the Perspectives of Young Siblings. *Child and Family Social Work, 22*(3), 1237-1245. doi:10.1111/cfs.12340

Mannarini, S., & Kleinbub, J. R. (2022). Parental-Bonding and Alexithymia in Adolescents with Anorexia Nervosa, Their Parents, and Siblings. *Behavioral Sciences, 12*(123). doi:<https://doi.org/10.3390/bs12050123>

Matthews, A., Peterson, C. M., Lenz, K., Kramer, R. A., Mara, C., Copps, E., & Mitan, L. (2021). Modifiable Factors Associated with Mental Health Symptoms in Siblings of Adolescents with Anorexia Nervosa. *Eating and weight disorders, 26*(6), 1757-1765.

Meneguzzo, P., Sala, A., Merlino, L., Ceccato, E., & Santonastaso, P. (2022). One Year of Covid-19 Pandemic on Patients with Eating Disorders, Healthy Sisters, and Community Women: Evidence of Psychological Vulnerabilities. *Eating and weight disorders, 27*, 3429-3438.

Newman, S., Simonds, L. M., & Billings, J. (2011). A Narrative Analysis Investigating the Impact of First Episode Psychosis on Siblings’ Identity. *Psychosis, 3*(3), 216-225. doi:10.1080/17522439.2010.542588

Oshodi, Y. O., Adeyemi, J. D., Aina, O. F., Suleiman, T. F., Erinfolami, A. R., & Umeh, C. (2012). Burden and Psychological Effects: Caregiver Experiences in a Psychiatric Outpatient Unit in Lagos, Nigeria. *African Journal of Psychiatry, 15*(2), 99-105.

Panaite, V., Bylsma, L. M., Kovacs, M., O'Leary, K., George, C. J., Baji, I., . . . Vetró, Á. (2019). Dysregulated Behavioral Responses to Hedonic Probes among Youth with Depression Histories and Their High-Risk Siblings. *Emotion, 19*(1), 171-177.

Park, M., & Lee, K. J. (2017). Korean Sibling Caregivers of Individuals Diagnosed with Schizophrenia. *Asian Pac Isl Nurs J, 2*(3), 97-102. doi:10.9741/23736658.1066

Paswan, U. (2022). A Prospective Assessment of the Psychological Distress and Caregiver Burden among Care Givers of Schizophrenia Patients. *International Journal of Pharmaceutical and Clinical Research, 14*(2), 112-119.

Phoeun, B., Chanthorn, L., Schulhofer, L., Khann, S., Soung, T., Conroy, K., & Nguyen, A. J. (2022). ‘I Feel Hopeless’: Exploring the Psychosocial Impacts of Caring for Mentally Ill Relatives in Cambodia. *International Journal of Social Psychiatry, 69*(2), 438-446.

Rachamim, L., Nacasch, N., & Sinay, I. (2021). Complicated Grief, Depression, Health and Attachment Style in First Degree Relatives of Individuals with a Chronic Psychotic Disorders. *Community Mental Health Journal*. doi:10.1007/s10597-021-00848-z

Reinhard, S. C., & Horwitz, A. V. (1995). Caregiver Burden: Differentiating the Content and Consequences of Family Caregiving. *Journal of Marriage and the Family, 57*(3), 741-750.

Ribé, J. M., Salamero, M., Pérez-Testor, C., Mercadal, J., Aguilera, C., & Cleris, M. (2018). Quality of Life in Family Caregivers of Schizophrenia Patients in Spain: Caregiver Characteristics, Caregiving Burden, Family Functioning, and Social and Professional Support. *International Journal of Psychiatry in Clinical Practice, 22*(1), 25-33.

Scheirs, J. G., & Bok, S. (2007). Psychological Distress in Caretakers or Relatives of Patients with Borderline Personality Disorder. *International Journal of Social Psychiatry, 53*(3), 195-203. doi:10.1177/0020764006074554

Scutt, E., Langdon-Daly, J., & Smithson, J. (2022). Experiences of Eating Difficulties in Siblings of People with Anorexia Nervosa: A Reflexive Thematic Analysis. *Journal of eating disorders, 10*(123). doi:<https://doi.org/10.1186/s40337-022-00646-2>

Seltzer, M. M., Greenberg, J. S., Krauss, M. W., Gordon, R. M., & Judge, K. (1997). Siblings of Adults with Mental Retardation or Mental Illness: Effects on Lifestyle and Psychological Well-Being. *Family Relations: An Interdisciplinary Journal of Applied Family Studies, 46*(4), 395-405. doi:10.2307/585099

Shah, S. T. H., Sultan, S. M., Faisal, M., & Irfan, M. (2013). Psychological Distress among Caregivers of Patients with Schizophrenia. *Journal of Ayub Medical College, 25*(3-4), 27-30.

Shivers, C., Russon, J., Benson, M. J., King, A., & Textoris, S. (2022). Siblings’ Role Positions and Perceptions of Mental Illness. *Contemporary Family Therapy*. doi:<https://doi.org/10.1007/s10591-022-09637-6>

Sletved, K. S. O., Coello, K., Stanislaus, S., Kjærstad, H. L., Melbye, S. A., Faurholt-Jepsen, M., . . . Kessing, L. V. (2022). Socio-Economic Status and Functioning in Patients Newly Diagnosed with Bipolar Disorder and Their Unaffected Siblings: Results from a Cross-Sectional Clinical Study. *Journal of Affective Disorders, 310*, 404-411.

Tanaka, K. (2008). Resilience among Siblings of Persons with Serious Mental Illness: A Cross-National Comparison. *The International Journal of Interdisciplinary Social Sciences, 4*(12), 61-74.

Taylor, J. L., Greenberg, J. S., Seltzer, M. M., & Floyd, F. J. (2008). Siblings of Adults with Mild Intellectual Deficits or Mental Illness: Differential Life Course Outcomes. *Journal of Family Psychology, 22*(6), 905-914.

The World Bank. (n.d.). Country Classification by Income. Retrieved from <https://datahelpdesk.worldbank.org/knowledgebase/articles/906519>

Titelman, D., & Psyk, L. (1991). Grief, Guilt, and Identification in Siblings of Schizophrenic Individuals. *Bulletin of the Menninger Clinic, 55*(1), 72-84.

van Sprang, E. D., Maciejewski, D. F., Milaneschi, Y., Kullberg, M.-L., Hu, M. X., Elzinga, B. M., . . . Penninx, B. W. J. H. (2021). Familial Resemblance in Mental Health Symptoms, Social and Cognitive Vulnerability, and Personality: A Study of Patients with Depressive and Anxiety Disorders and Their Siblings. *Journal of Affective Disorders, 294*, 420-429.

Vermeulen, J., Schirmbeck, F., Blankers, M., van Tricht, M., van den Brink, W., de Haan, L., . . . van Winkel, R. (2019). Smoking, Symptoms, and Quality of Life in Patients with Psychosis, Siblings, and Healthy Controls: A Prospective, Longitudinal Cohort Study. *The Lancet Psychiatry, 6*(1), 25-34.

Vinberg, M., Bech, P., Kyvik, K. O., & Kessing, L. V. (2007). Quality of Life in Unaffected Twins Discordant for Affective Disorder. *Journal of Affective Disorders, 99*(1-3), 133-138. doi:10.1016/j.jad.2006.08.033

Vothknecht, S., Meijer, C., Zwinderman, A., Kikkert, M., Dekker, J., van Beveren, N., . . . For, G. (2013). Psychometric Evaluation of the Subjective Well-Being under Neuroleptic Treatment Scale (Swn) in Patients with Schizophrenia, Their Relatives and Controls. *Psychiatry Research, 206*(1), 62-67. doi:10.1016/j.psychres.2012.09.004

World Health Organization. (2018). *Standards for Improving the Quality of Care for Children and Young Adolescents in Health Facilities*. Geneva, Switzerland: World Health Organization.

Zauszniewski, J. A., & Bekhet, A. K. (2014). Factors Associated with the Emotional Distress of Women Family Members of Adults with Serious Mental Illness. *Archives of psychiatric nursing, 28*(2), 102-107.

Ziermans, T. B., Schirmbeck, F., Oosterwijk, F., Geurts, H. M., de Haan, L., Risk, G., & Investigators, O. o. P. (2021). Autistic Traits in Psychotic Disorders: Prevalence, Familial Risk, and Impact on Social Functioning. *Psychological medicine, 51*(10), 1704-1713.
